# Synthesis and in vitro evaluation of pharmacokinetically optimized ^99m^Tc-labeled cholecystokinin-2 receptor-targeted peptides for improved SPECT imaging

**DOI:** 10.1186/s41181-026-00477-5

**Published:** 2026-07-20

**Authors:** Veronika Felber, Nadine Holzleitner, Thomas Günther

**Affiliations:** 1 TUM School of Natural Sciences, Department of Chemistry, Chair of Pharmaceutical Radiochemistry, Technical University of Munich, Walther-Meißner-Str. 3, 85748 Garching, Germany; 2 Department of Nuclear Medicine, TUM University Hospital and Central Institute for Translational Cancer Research, (TranslaTUM), School of Medicine, Technical University of Munich, Munich, Germany; 3 Molecular Imaging Program at Stanford (MIPS), Department of Radiology, School of Medicine, Stanford University, Stanford, CA USA

**Keywords:** Cholecystokinin-2 receptor, CCK-66, Medullary thyroid carcinoma, Diagnosis, SPECT, Technetium-99 m, Peptide receptor radionuclide therapy

## Abstract

**Background:**

Hitherto, clinically applied ^99m^Tc-based cholecystokinin-2 receptor (CCK-2R) ligands suffer from limited in vivo stability. Therefore, we focused on the development of pharmacokinetically optimized ^99m^Tc-labeled CCK-2R ligands for improved imaging of CCK-2R-overexpressing malignancies via single-photon emission computed tomography (SPECT). The novel CCK-2R ligands were designed based on the recently published compounds CCK-66, rhCCK-18 and rhCCK-84.

**Results:**

CCK-2R ligands were prepared by solid-phase peptide synthesis (SPPS) and ^99m^Tc-labeled via the tetraamine (N_4_) chelator (95 °C, 15 min) with final radiochemical purities ≥ 95% (radiochemical yields > 99%, n = 39), as determined by radio-RP-HPLC. Affinity (IC_50,inverse_) and internalization experiments were performed using CCK-2R^+^ AR42J cells. Lipophilicity (logD_7.4_), human serum albumin (HSA) binding and stability in human serum (4 h, 37 °C) was determined as well. Four of the seven novel CCK-2R ligands, i.e. [^99m^Tc]Tc-N_4_-CCK-66 and [^99m^Tc]Tc-N_4_-CCK-103 to -105, exhibited similar CCK-2R affinities compared to the reference ligands [^177^Lu]Lu-DOTA-PP-F11N and [^177^Lu]Lu-DOTA-CCK-66 (IC_50,inverse_: 28.2–40.3 nM). [^99m^Tc]Tc-N_4_-CCK-66 and [^99m^Tc]Tc-N_4_-CCK-104 demonstrated high CCK-2R-mediated internalization (223.8 ± 0.1% and 145.2 ± 0.1% compared to [^177^Lu]Lu-DOTA-PP-F11N, respectively), high stability in human serum (> 95%) and similar lipophilicity (log*D*_7.4_: − 2.09 to − 2.42) and HSA binding (47.5–53.2%) compared with [^177^Lu]Lu-DOTA-CCK-66. 4-(Di-*tert*-butyl(hydroxy)silyl)benzoic acid (SiOH-BA)-bearing compounds [^99m^Tc]Tc-N_4_-CCK-100 and [^99m^Tc]Tc-N_4_-CCK-103 showed an elevated lipophilicity (logD_7.4_: − 1.89 to − 0.95) and HSA binding (78.1–89.1%) as well as a reduced stability in human serum (87.0–93.0%), but higher internalization than [^177^Lu]Lu-DOTA-PP-F11N (145.0 ± 0.1% and 219.5 ± 0.1%, respectively).

**Conclusions:**

Several ^99m^Tc-labeled CCK-2R ligands with very favorable in vitro characteristics were successfully developed in this study. In particular, [^99m^Tc]Tc-N_4_-CCK-66 and [^99m^Tc]Tc-N_4_-CCK-104 are highly recommended for further evaluation in in vivo studies to examine their potential as ^99m^Tc-based SPECT agents for improved imaging of CCK-2R-expressing malignancies. Furthermore, a possible radiotheranostic use of these N_4_-conjugated compounds could be explored in future studies using ^186/188^Re.

**Supplementary Information:**

The online version contains supplementary material available at 10.1186/s41181-026-00477-5.

## Introduction

**Fig. 1:**
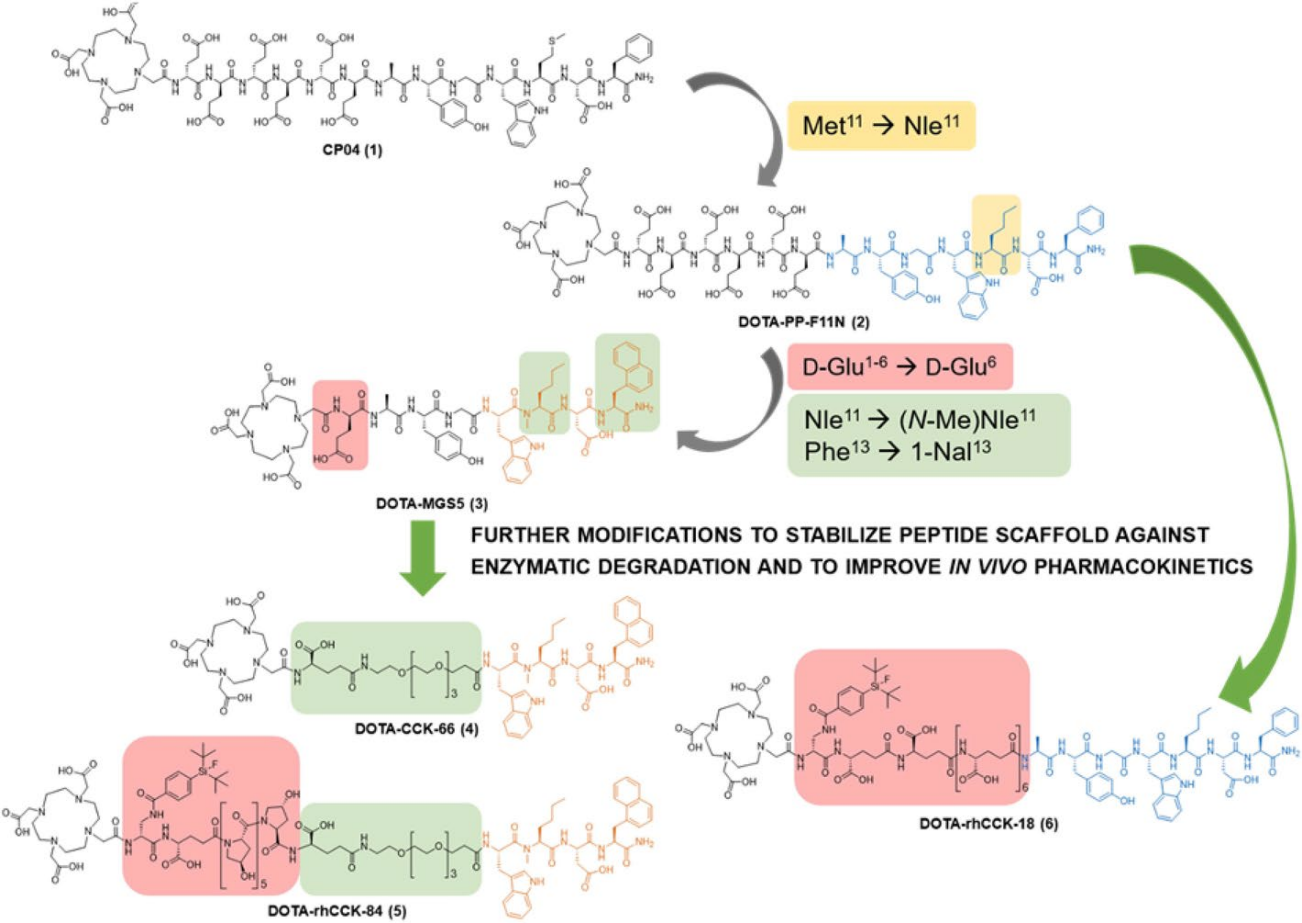
Chemical structures of CP04 (**1**), DOTA-PP-F11N (**2**), DOTA-MGS5 (**3**), DOTA-CCK-66 (**4**), DOTA-rhCCK-84 (**5**) and DOTA-rhCCK-18 (**6**) and modifications which improved their in vivo stability and pharmacokinetics. Compounds **1** and **2** were developed by Béhé and Schibli (2015) in 2015 and compound **3** by Klingler et al. (2019a) in 2019. Further modifications, conducted by Günther and Holzleitner et al. (2024, 2023; Holzleitner 2023) since 2023 (green arrows), resulted in compounds **4**,** 5** and** 6**. Common CCK-2R binding motifs are displayed in orange or blue. Sites that have been modified to stabilize the peptide against metabolic degradation (oxidation of methionine) are highlighted in yellow, sites that have been modified to stabilize the peptide against enzymatic cleavage are highlighted in green and sites that have been modified to improve in vivo pharmacokinetics are highlighted in red

In recent years, tremendous progress has been made in the development of pharmacokinetically optimized lead structures targeting the CCK-2 receptor. Ligands such as [^111^In]In-CP04 (NCT03246659) (https:, , clinicaltrials.gov 2025. 2025; Lezaic et al. 2023), [^177^Lu]Lu-DOTA-PP-F11N (NCT03647657, NCT02088645) (https:, , clinicaltrials.gov 2025. 2025; Rottenburger et al. 2020) and [^68^Ga]Ga-DOTA-MGS5 (NCT06155994, NCT06520319) (https:, clinicaltrials.gov 2025. 2025; Guggenberg et al. 2023) have entered clinical phase I and/or II studies. The metabolically more stable DOTA-MGS5, radiolabeled with different radiometals such as ^111^In for SPECT, ^68^Ga for PET or β^−^-emitting radionuclides such as ^177^Lu has been developed for improved diagnosis and endoradiotherapy of patients with progressive or metastatic medullary thyroid carcinoma (MTC), as well as other advanced-stage CCK-2R-expressing malignancies.(Klingler et al. 2019a) Very recently, we have developed modified CCK-2R ligands such as DOTA-CCK-66 (Fig. 1), which revealed similar stability in murine serum compared with [^177^Lu]Lu-DOTA-MGS5 (78.5 ± 3.1% versus (vs.) 82.0 ± 0.1% intact at 30 min *post injectionem* (p.i.), respectively), while it was cleared less metabolized (77.8 ± 2.3% vs. 23.7 ± 9.2% intact in murine urine at 30 min p.i., respectively) (Günther et al. 2024) Both [^68^Ga]Ga-DOTA-MGS5 and [^68^Ga]Ga-DOTA-CCK-66 have shown clinical value in small patient cohorts suffering from MTC or small cell lung cancer.(Viering et al. 2024, 2025; Viering et al. xxxx; Uprimny et al. 2025; Di Santo et al. 2024).

Despite this progress and a certain predominance of SPECT in the global nuclear medicine equipment market over positron emission tomography (PET) and other imaging modalities, no ^99m^Tc-labeled CCK-2R ligand has yet entered clinical trials (https:, , www.grandviewresearch.com, industry-analysis, nuclear-medicine-equipment-market, accessed on 08. August 2025. 2025) Due to lower acquisition costs for SPECT devices and the good availability of the generator-produced radionuclide ^99m^Tc at low cost, SPECT remains particularly attractive for smaller nuclear medicine facilities.(Duatti 2021; Liu and Edwards 1999) ^99m^Tc is still the most extensively used diagnostic isotope in SPECT applications, accounting for approximately 80% of all nuclear medicine examinations worldwide, with an estimated annual increase of 3–8% (Liu and Edwards 1999; Nawar and Türler 2022) Hence, ^99m^Tc-labeled CCK-2R ligands could be an attractive alternative if PET is not available.

**Fig. 2:**
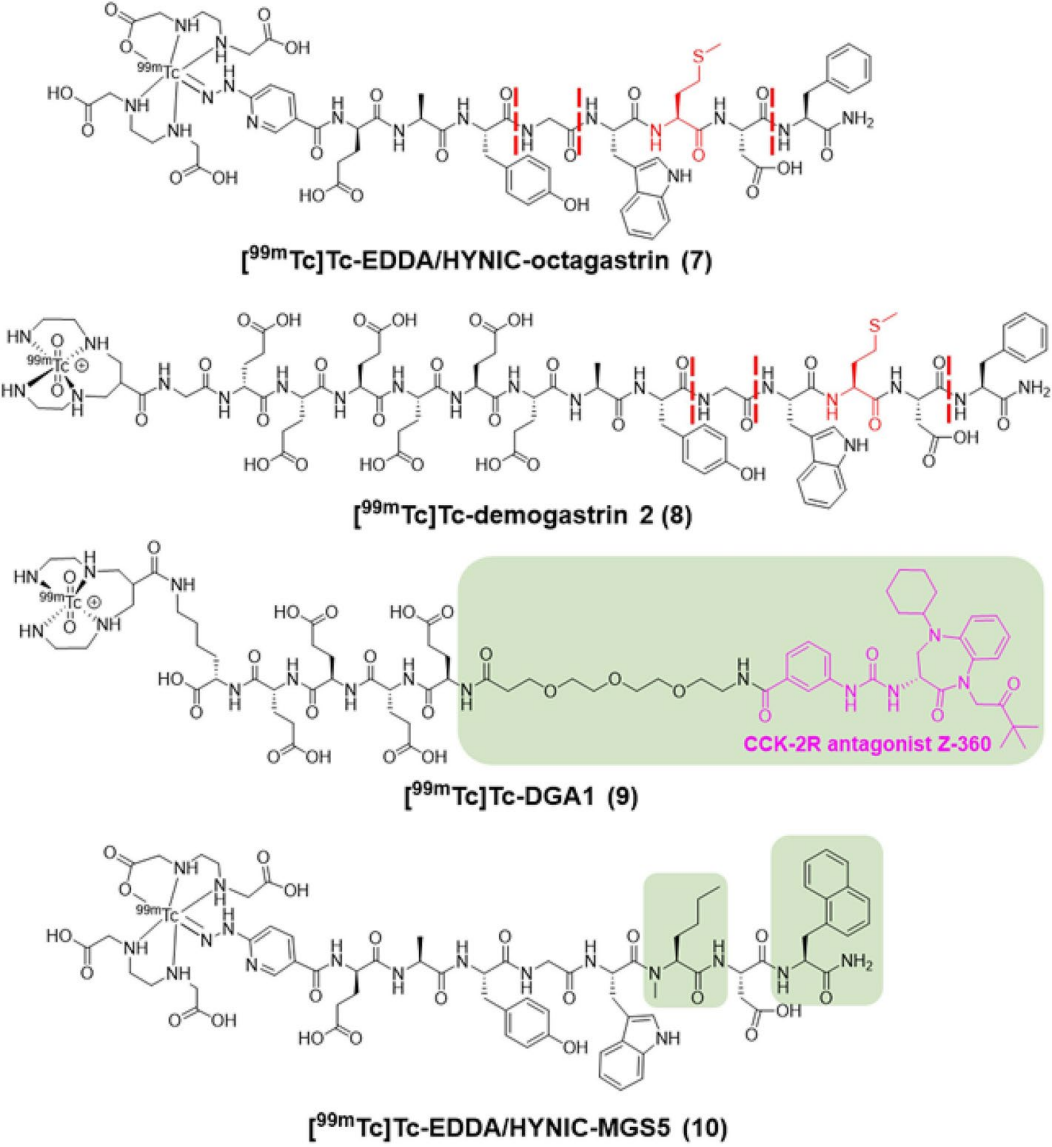
Chemical structures of ^99m^Tc-labeled CCK-2R ligands, either already applied in patients ([^99m^Tc]Tc-EDDA/HYNIC-octagastrin (**7**) and [^99m^Tc]Tc-demogastrin 2 (**8**)), or comprising a N_4_ chelator system for ^99m^Tc-complexation ([^99m^Tc]Tc-demogastrin 2 (**8**) and [^99m^Tc]Tc-DGA1 (**9**)), or stabilized towards enzymatic degradation ([^99m^Tc]Tc-DGA1 (**9**) and [^99m^Tc]Tc-EDDA/HYNIC-MGS5 (**10**)). Of these ligands, ^99m^Tc-demogastrin 2 (**8**) represents the clinically most advanced ^99m^Tc-labeled CCK-2R ligand. Sites of enzymatic cleavage in human serum and of other metabolic degradation (oxidation of methionine) are depicted in red.(Ocak et al. 2011) Sites that have been modified to stabilize the peptide against enzymatic cleavage are highlighted in green. The non-peptidic CCK-2R antagonist Z-360 in DGA1 is displayed in magenta

Hitherto, CCK-2R ligands for ^99m^Tc-based SPECT are mainly based on minigastrin (MG) derivatives equipped with the N_4_, EDDA/HYNIC or (N_α_-His)acetic acid chelator system for ^99m^Tc complexation (Guggenberg et al. 2004, 2007, 2009; Nock et al. 2005; Kosowicz et al. 2007; Klingler et al. 2019b) So far, only [^99m^Tc]Tc-EDDA/HYNIC-octagastrin ([^99m^Tc]Tc-EDDA/HYNIC-D-Glu-Ala-Tyr-Gly-Trp-Met-Asp-Phe-NH_2_) and [^99m^Tc]Tc-demogastrin 2 ([^99m^Tc]Tc-N_4_-Gly-D-Glu-(Glu)_5_-Ala-Tyr-Gly-Trp-Met-Asp-Phe-NH_2_) were administered to MTC patients (Fig. 2).

While [^99m^Tc]Tc-EDDA/HYNIC-octagastrin, revealed insufficient sensitivity, [^99m^Tc]Tc-demogastrin 2 provided notably good delineation of tumor lesions in a patient with metastatic MTC.(Nock et al. 2005; Kosowicz et al. 2007) More recent studies on [^99m^Tc]Tc-EDDA/HYNIC-MGS5 ([^99m^Tc]Tc-EDDA/HYNIC-D-Glu-Ala-Tyr-Gly-Trp-(*N*-Me)Nle-Asp-1-Nal-NH_2_) and [^99m^Tc]Tc-DGA1 (Fig. 2) demonstrated high *in* *vivo* stabilities at early sampling time points (~ 65% vs. 97.0 ± 0.5% intact, respectively, in the blood of BALB/c mice at 10 min p.i. or in the blood of Swiss Albino mice at 5 min p.i. for [^99m^Tc]Tc-DGA1).(Klingler et al. 2019b; Kaloudi et al. 2020) However, none of them were applied in patients so far.

**Fig. 3:**
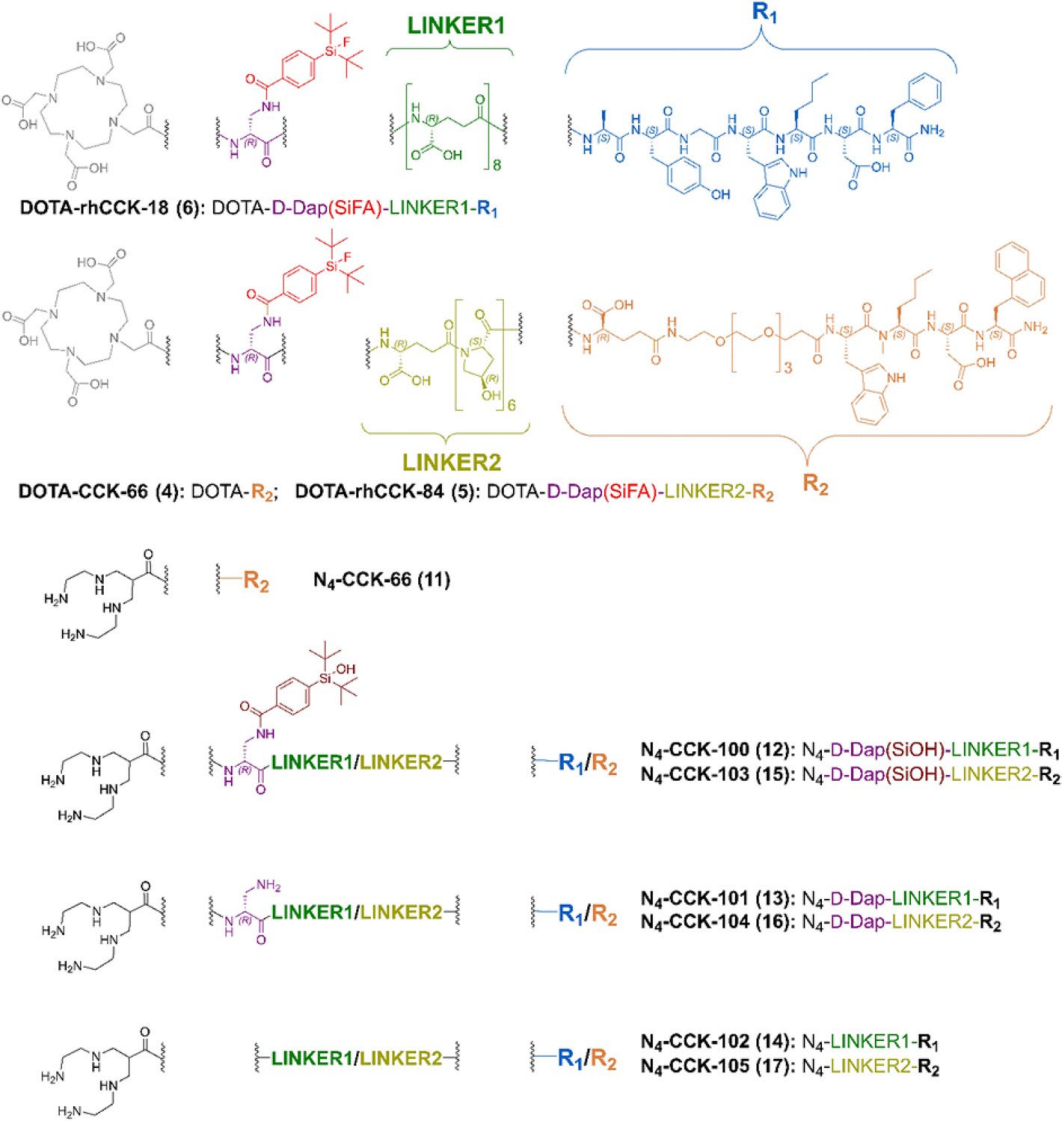
Chemical structures of novel CCK-2R-targeted precursor compounds for ^99m^Tc labeling. All of these peptides are either based on CCK-66, rhCCK-18 or rhCCK-84 (structures shown in the top two rows), which in turn are based on the literature-known CCK-2R-targeted compounds PP-F11N (Behe and Schibli 2015) or MGS5(Klingler et al. 2019a). The derivatives either contain the CCK-2R binding motif based on rhCCK-18 (**R**_**1**_, blue) or CCK-66/rhCCK-84 (**R**_**2**_, orange). Different linker sequences are displayed in dark green ((D-γ-Glu)_8_)) or light green (D-γ-Glu-(Hyp)_6_). The novel compounds (N_4_-CCK-66 and -100 to -105 (**11**–**17**)) are differing in the presence or absence of D-2,3-diaminopropionic acid (D-Dap, purple) to which a hydrolyzed silicon-based fluoride acceptor moiety (SiOH, dark red, **12** and **15**) is attached or omitted. The non-hydrolyzed silicon-based fluoride acceptor (SiFA) is displayed in compounds **5** and **6** (red). All ^99m^Tc-precursors are equipped with a N_4_ chelator (black)

Here we report on the development of novel ^99m^Tc-labeled CCK-2R ligands with improved in vivo stability and pharmacokinetic profile, with their design being based on previously developed compounds, i.e. CCK-66, rhCCK-18 and rhCCK-84. These CCK-2R ligands were either already applied in patients (CCK-66) or revealed very favorable preclinical data (rhCCK-18, rhCCK-84) (Günther et al. 2024, 2023; Holzleitner 2023) In particular, CCK-66- and rhCCK-84-based compounds are characterized by a peptide scaffold, in which distinct enzymatic cleavage sites have been eradicated (e.g. by introduction of a polyethylene glycol (PEG) linker) and/or pharmacokinetic modifiers (e.g. a silicon-based fluoride acceptor (SiFA) unit or a poly-hydroxyproline (Hyp) linker) have been introduced to increase in vivo stability and tumor uptake. Thus, a notably enhanced stability at 30 min p.i. could be obtained for ^nat^F-[^177^Lu]Lu-DOTA-rhCCK-84 (93.5 ± 2.1% intact in serum and 55.4 ± 9.0% intact in urine of healthy CB17-SCID mice). Also ^nat^F-[^177^Lu]Lu-DOTA-rhCCK-18 remained intact in a lower but still considerable amount (64.7 ± 14.6% intact in serum and 15.9 ± 5.6% intact in urine of healthy CB17-SCID mice) at 30 min p.i. (Holzleitner 2023) Under the same experimental conditions, 82.0 ± 0.1% intact [^177^Lu]Lu-DOTA-MGS5 and 78.5 ± 3.1% intact [^177^Lu]Lu-DOTA-CCK-66 could be found in murine serum. In murine urine, 23.7 ± 9.2% of [^177^Lu]Lu-DOTA-MGS5 and 77.8 ± 2.3% of [^177^Lu]Lu-DOTA-CCK-66 remained intact (Günther et al. 2024) Moreover, these compounds showed substantial activity uptake in AR42J tumor xenografts at 24 h p.i. in CB17-SCID mice (25.4 ± 4.7% ID/g for ^nat^F-[^177^Lu]Lu-DOTA-rhCCK-18, 18.8 ± 1.4% ID/g for ^nat^F-[^177^Lu]Lu-DOTA-rhCCK-84 and 8.56 ± 0.0% ID/g for [^177^Lu]Lu-DOTA-CCK-66). Notably, [^177^Lu]Lu-DOTA-CCK-66 reached similar tumor-to-kidney ratios as obtained for [^177^Lu]Lu-DOTA-MGS5 (7.82 ± 0.70 and 9.35 ± 3.28 at 24 h p.i., respectively) (Günther et al. 2024; Holzleitner 2023) Therefore, we focused on the development and preclinical evaluation of pharmacokinetically optimized CCK-2R ligands, based on the recently published compounds CCK-66, rhCCK-18 and rhCCK-84 and which can be labeled with ^99m^Tc (Fig. 3).

The open-chain tetraamine (N_4_) chelator enables fast, efficient, coligand-free ^99m^Tc-labeling with high in vivo stability, as shown for multiple clinically applied radioligands (Mather et al. 2014; Rinscheid et al. 2024; Konrad et al. 2023; Decristoforo et al. 2003; Novak et al. 2023) Besides, the formation of isostructural technetium and rhenium complexes with this chelator offers the possibility to eventually obtain the respective ^186/188^Re-based therapeutic agents (theranostic concept) (Melis et al. 2022; Prakash et al. 1996).

Adjacent to the N_4_ chelator we introduced either a neutral, lipophilic residue (D-Dap(SiOH)), a positively charged unit (D-Dap) or a negatively charged residue (D-γ-Glu) to investigate potential effects on in vitro/in vivo behavior of the resulting derivatives.

A hydrolyzed silicon-based fluoride acceptor (SiOH) was installed as pharmacokinetic modifier instead of the original, non-hydrolyzed SiFA, to partly compensate for the highly lipophilic character of the N_4_ chelator.

## Materials and methods

Detailed information on all methods for synthesis and analysis as well as on the instruments used, is available in the supporting information (available on https://ejnmmipharmchem.springeropen.com). Analytical data (Figures S1–S55) and methods for in vitro characterization are also given in the supporting information (SI).

### Chemical synthesis

The CCK-2R ligands were prepared according to optimized protocols via manual or automated solid-phase peptide synthesis (SPPS). N_4_Boc_4_ (= N_4_ chelator precursor) as well as SiOH-BA were prepared separately in solution and purified by flash chromatography or semi-preparative reversed-phase high-performance liquid chromatography (RP-HPLC), respectively, before coupling to the resin-bound peptide. Final purification of the peptides was achieved by semi-preparative RP-HPLC. Characterization of all compounds was conducted via analytical RP-HPLC in combination with electrospray ionization mass spectrometry (ESI–MS). In addition, ^1^H- and ^13^C-NMR spectra were acquired for analysis of the purified N_4_ chelator, whereas ^1^H-, ^19^F- and ^19^F{^1^H}-NMR spectra were acquired for SiOH-BA and its non-hydrolyzed precursor 4-(di-*tert*-butylfluorosilyl)benzoic acid (SiFA-BA).

### ^nat^Lu-complexation

Quantitative ^nat^Lu-complexation was conducted by adding a solution of ^nat^LuCl_3_ (20 mM in Tracepur®-H_2_O, 2.5 eq.) to the DOTA-bearing peptide precursor (1.0 mM or 2.0 mM in DMSO, 1.0 eq.). Tracepur®-H_2_O was added to reach a final peptide concentration of 0.5 mM or 1.0 mM and the reaction mixture was incubated at 95 °C for 30 min. Chemical purity and identity were analyzed via analytical RP-HPLC and ESI–MS, and the ^nat^Lu-complexed compounds (0.5 or 1.0 mM in DMSO/Tracepur®-H_2_O = 1/1) were directly used as stock solutions for affinity determinations (IC_50,inverse_), internalization studies or for HPLC co-injections with their ^177^Lu-labeled analogs (confirmation of identity), as chemical purity was always > 98% (n = 5) and remaining free Lu^3+^ was shown to have no impact on IC_50_ determinations (Günther et al. 2022a).

### Radiolabeling

^*177*^*Lu-labeling.* The ^177^Lu-labeled reference ligands [^177^Lu]Lu-DOTA-PP-F11N ([^177^Lu]Lu-**2**) and [^177^Lu]Lu-DOTA-CCK-66 ([^177^Lu]Lu-**4**) were prepared according to established procedures for CCK-2R-targeted ligands(Günther et al. 2024, 2023), with some minor modifications. Briefly, 1.0 µL of the precursor (**2** or** 4**) (1.0 mM in DMSO, 1.0 nmol) was added to 10 µL of 1 M NaOAc buffer (aq.) (pH = 5.5). Approximately 50 MBq [^177^Lu]LuCl_3_ (no-carrier-added (n.c.a.), A_s_ > 3000 GBq/mg, 740 MBq/mL, 0.04 M HCl, ITM Pharma Solutions GmbH, Garching, Germany) were added and the mixture was adjusted to a total volume of 100 µL using 0.04 M HCl (in Tracepur®-H_2_O). After radiolabeling at 90 °C for 15 min, 10 µL of 0.1 M sodium L-ascorbate (in PBS) was added. Radiochemical purity was determined using radio-RP-HPLC and radio-TLC (more details are given in the SI). The pH of the final solution was 5 and it was used without any further purification.

^*99m*^*Tc-labeling.* At the day of experiment, [^99m^Tc]TcO_4_^−^ was freshly eluted from a ^99^Mo/^99m^Tc generator using isotonic saline solution (0.9% NaCl in Tracepur®-H_2_O). The radionuclide generator (Ultra-Technekow FM, 2.15–43.00 GBq, Curium, Petten, Netherlands) was provided by the *Klinikum rechts der Isar* (Technical University of Munich, Germany) and was regularly eluted at 12- to 24-h intervals. ^99m^Tc-Labeling of N_4_-conjugated CCK-2R ligands was conducted by adding 1.0 µL precursor (1.0 mM in DMSO, 1.0 nmol) to a mixture of 12.5 µL of Na_2_HPO_4_ (0.05 M in Tracepur®-H_2_O, pH = 9.5), 1.5 µL of disodium citrate sesquihydrate (0.1 M in Tracepur®-H_2_O, pH = 5.5) and 2.5 µL of SnCl_2_ (1 mg/mL in ethanol, freshly prepared on the day of the experiment). After the addition of approximately 50 MBq [^99m^Tc]TcO_4_^−^ in isotonic saline, further 0.9% NaCl in Tracepur®-H_2_O was added to reach a total volume of 250 µL. The labeling solution was incubated at 95 °C for 15 min. Subsequently, 10 µL of 0.1 M sodium L-ascorbate (in PBS) was added and the radiochemical purity was determined using radio-RP-HPLC and radio-TLC (more details are given in the SI). The pH of the final solution was 6–7 and it was used without any further purification.

### In vitro experiments

A detailed description of all in vitro experiments is provided in the SI. Lipophilicity determinations as well as stability studies in human serum were conducted as previously described. (Günther et al. 2024, 2023; Holzleitner et al. 2022) For affinity determinations, internalization studies and binding to human serum albumin (HSA), the previously published procedures had to be slightly modified. The novel CCK-2R ligands to be investigated in this study are labeled with ^99m^Tc, for which non-radioactive analogs were not available (no stable, non-radioactive isotope of technetium existing).

As a consequence, the standard competitor ^nat^Lu-DOTA-PP-F11N was applied in its non-radiolabeled form in increasing concentrations (10^–5^–10^–11^ M/well, n = 3 each), whereas the novel CCK-2R-binding compounds of interest were applied as ^99m^Tc-labeled radioligands (1.2 nM/well) for affinity determinations using AR42J cells. In this inversed experimental approach, higher values correspond to higher affinities and are referred to as IC_50,inverse_. [^177^Lu]Lu-DOTA-PP-F11N as well as [^177^Lu]Lu-DOTA-CCK-66, both for which regular IC_50_ values are available (for the respective ^nat^Lu-labeled derivatives), were also evaluated in this assay to generate reference IC_50,inverse_ values and to compare regular IC_50_ with IC_50,inverse_ data.

To determine the cellular uptake of the ^99m^Tc-labeled peptides in AR42J cells by means of CCK-2R-mediated internalization at 1 h, a previously reported protocol(Günther et al. 2023) was used with concentrations of 1.2 nM/well for novel ^99m^Tc-labeled ligands and [^177^Lu]Lu-DOTA-PP-F11N (reference compound). Data were corrected for non-specific binding (10 µM/well of ^nat^Lu-DOTA-PP-F11N for ‘blocking’ conditions) and normalized to the specific internalization of the reference [^177^Lu]Lu-DOTA-PP-F11N.

In contrast to the previously published procedures, HSA binding was not determined by high-performance affinity chromatography (HPAC), as no non-radioactive analogs of the peptides of interest were available and HPAC data of the precursor compounds in their free-N_4_ chelator form were considered as less conclusive. Instead, binding to HSA was determined via ultrafiltration. Therefore, radioligands were incubated in a solution of HSA in PBS (700 µM) at 37 °C for 30 min and subsequent centrifugation (250 µL aliquots, 3200 rpm, 40 min, r.t.) in Centrifree® ultrafiltration devices. The fraction bound to HSA was calculated as the ratio of non-filtered, membrane-bound activity to the total activity in the ultrafiltration device. All values were corrected for non-specific binding (control experiments in PBS).

## Results

### Synthesis and radiolabeling

Fmoc-based SPPS with subsequent purification via RP-HPLC provided the peptide precursors (N_4_-CCK-66 (**11**), N_4_-CCK-100 to -105 (**12–17**), as well as DOTA-CCK-66 (**4**) and DOTA-PP-F11N (**2**)) in chemical yields ranging from 0.9 to 30.8% and in a chemical purity ≥ 95% (as determined by RP-HPLC at λ = 220 nm).

^nat^Lu-Complexation of DOTA-PP-F11N (**2**) and DOTA-CCK-66 (**4**) resulted in > 98% chemical purity (> 99% chemical yield, n = 5), as determined by RP-HPLC (λ = 220 nm).

^177^Lu-Labeling afforded the reference compounds [^177^Lu]Lu-DOTA-PP-F11N ([^177^Lu]Lu-**2**) and [^177^Lu]Lu-DOTA-CCK-66 ([^177^Lu]Lu-**4**) in quantitative radiochemical yields (RCY > 99%), RCPs ≥ 95% (as determined by radio-RP-HPLC and radio-TLC) and apparent molar activities (A_m_) of 49.6 ± 2.0 GBq/µmol (n = 6) at end of synthesis (EOS). For [^177^Lu]Lu-DOTA-PP-F11N, the radioligand stock solution was stored at -4 °C and used to a maximum of 2 days after EOS (RCP still > 95% as determined by radio-RP-HPLC, Table 1) for IC_50,inverse_ studies.

^99m^Tc-Labeling afforded [^99m^Tc]Tc-N_4_-CCK-66 and [^99m^Tc]Tc-N_4_-CCK-100 to -105 ([^99m^Tc]Tc-**11** to -**17**) in quantitative radiochemical yields (RCY > 99%), RCPs ≥ 95% (as determined by radio-RP-HPLC and radio-TLC for [^99m^Tc]Tc-**11**, − **12** and − **13**; for − **14** to − **17** radio-TLC-based RCPs partially reached only 87.7%-94.0%, *R*_f_(^99m^Tc-labeled peptide) = 0.9–1.0, Sect. 3.2 and 3.3 in the SI) and apparent A_m_ of 45.2 ± 4.8 GBq/µmol (n = 39) at EOS.

Analytical data on non-radioactive and radiolabeled compounds are provided in the SI.

### In vitro experiments

**Fig. 4:**
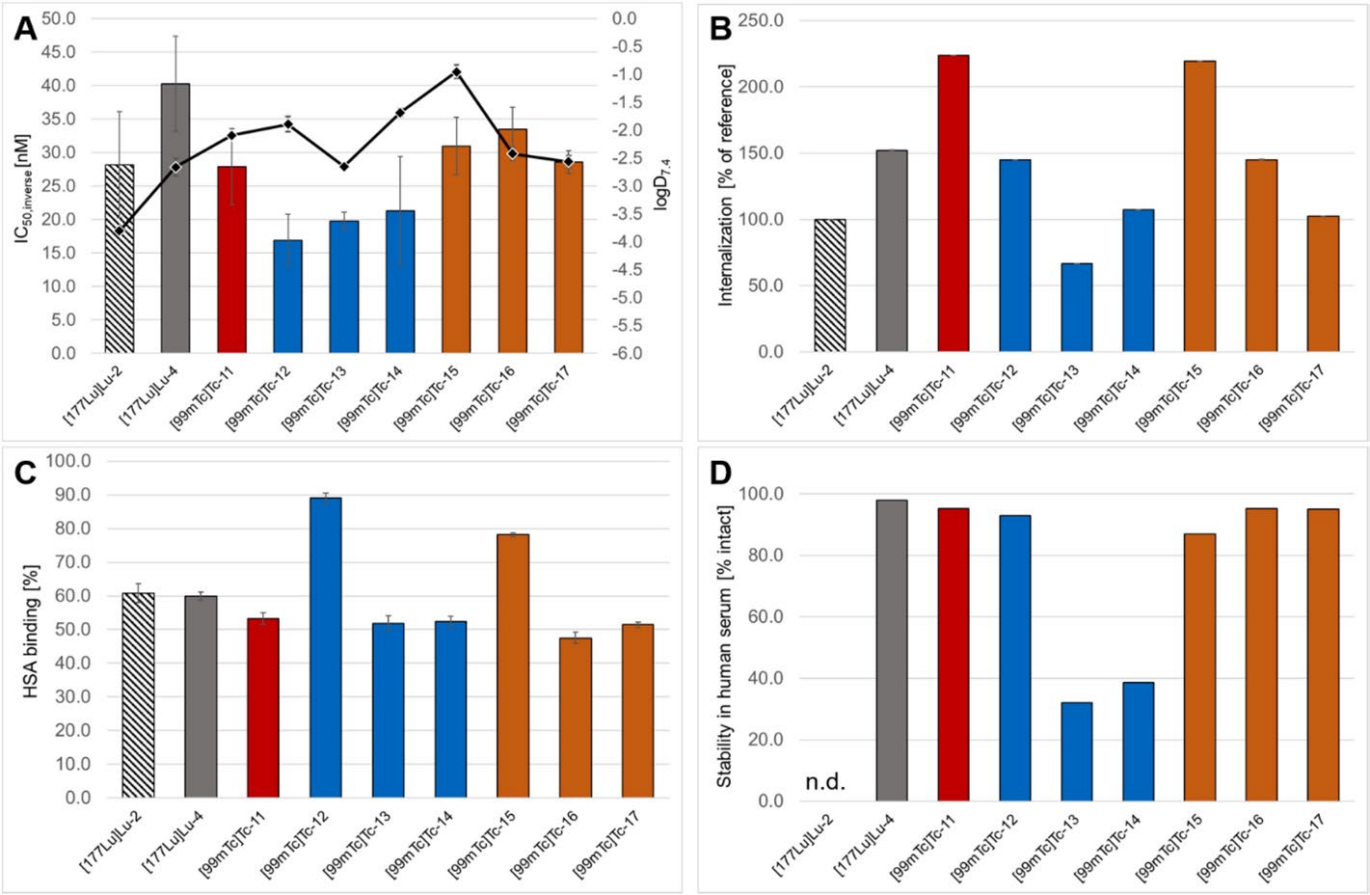
In vitro results of ^177^Lu-labeled reference compounds (black hatched and gray) and novel ^99m^Tc-labeled CCK-2R derivatives (red, blue and orange) with (**A**) CCK-2R-binding affinities (IC_50,inverse_) and lipophilicity (logD_7.4_), (**B**) internalization (%), (**C**) HSA binding (%) and (**D**) stability in human serum (% intact). Internalization values (**B**) were corrected for unspecific binding and normalized to the external reference [^177^Lu]Lu-**2** (12.6 ± 0.4% of the applied activity as CCK-2 receptor-mediated internalization, which corresponds to 100%). Stability in human serum of [^177^Lu]Lu-**2** (**D**) could be not determined (n.d.). All in vitro data used for preparing Fig. 4 are listed in Table S2 in the SI

In vitro data of the compounds evaluated in this study are depicted in Fig. 4, Table 1 and Table S2.

**Table 1:** RCP of radioligand in stock solution after distinct time points (%) and radio-RP-HPLC retention times (t_*R*_) of the investigated CCK-2R-binding compounds

CCK-2R ligand	RCP of radioligand in labeling solution [%] (time after EOS, storage temperature)	t_*R*_ [HPLC]^a^
[^177^Lu]Lu-**2**	97.2 (2 d, 4 °C)	6.12
[^177^Lu]Lu-**4**	96.1 (2 d, 4 °C)	11.8
[^99m^Tc]Tc-**11**	99.0 (7 h, r.t.)	9.45
[^99m^Tc]Tc-**12**	97.1 (5 h, r.t.)	9.75
[^99m^Tc]Tc-**13**	n.d	7.61
[^99m^Tc]Tc-**14**	95.4 (6 h, r.t.)	7.78
[^99m^Tc]Tc-**15**	96.8 (6 h, r.t.)	10.4
[^99m^Tc]Tc-**16**	98.4 (6 h, r.t.)	8.93
[^99m^Tc]Tc-**17**	98.2 (5.5 h, r.t.)	9.15

^a^30-50% MeCN (2% H_2_O, 0.1% TFA) in H_2_O (0.1% TFA) in 20 min, MultoKrom 100–5 C18 column for ^177^Lu-labeled peptides; 20–80% MeCN (2% H_2_O, 0.1% TFA) in H_2_O (0.1% TFA) in 15 min, MultoHigh Bio 300–5 C4 column for ^99m^Tc-labeled peptides

*CCK-2R affinity.* In this inversed experimental approach, ^nat^Lu-DOTA-PP-F11N was used as non-radioactive standard competitor and higher IC_50,inverse_ values correspond to higher affinities. The reference compounds [^177^Lu]Lu**-2** and [^177^Lu]Lu**-4** revealed an IC_50,inverse_ of 28.2 ± 7.9 nM and 40.3 ± 7.1 nM, respectively, with the latter exhibiting the highest CCK-2R affinity of all investigated compounds. The novel ^99m^Tc-CCK-2R ligands showed affinities in the range of [^177^Lu]Lu-**2**, with higher affinities (27.9–33.5 nM) obtained for derivatives equipped with the CCK-66/rhCCK-84 binding motif (**R**_**2**_, Fig. 4). ^99m^Tc-labeled compounds based on the rhCCK-18 binding motif (**R**_**1**_, Fig. 4) demonstrated lower IC_50,inverse_ values (16.9–21.3 nM), with [^99m^Tc]Tc-**12** revealing the lowest CCK 2R affinity (16.9 ± 3.9 nM).

*Internalization.* With the exception of [^99m^Tc]Tc-**13** (66.8 ± 0.04%), a similar ([^99m^Tc]Tc-**14** and [^99m^Tc]Tc-**17**) or notably higher internalization (145.0—223.8% for [^99m^Tc]Tc-**11**, -**12**, -**15** and -**16**) was obtained for the novel ^99m^Tc-CCK-2R ligands when compared to [^177^Lu]Lu**-2**. Thereof, [^99m^Tc]Tc-N_4_-CCK-66 ([^99m^Tc]Tc-**11**) displaying the highest CCK-2R-mediated internalization (223.8 ± 0.1%) among all compounds tested.

*Lipophilicity.* With the exception of [^99m^Tc]Tc-**12**, -**14** and -**15**, all compounds exhibited a logD_7.4_ below -2. In general, incorporation of the N_4_ chelator or the SiOH-BA moiety led to an increase in lipophilicity. The LINKER1**-R**_**1**_ system in [^99m^Tc]Tc-**12** appears to compensate more effectively for this increased lipophilicity than the LINKER2-**R**_**2**_ system in [^99m^Tc]Tc-**15**. Omission of the D-Dap unit directly adjacent to the N_4_ chelator induced an increased lipophilicity for [^99m^Tc]Tc-**14** (logD_7.4_: −1.68 ± 0.07 vs. −2.65 ± 0.06 for [^99m^Tc]Tc-**13**), while [^99m^Tc]Tc-**17** was not affected (logD_7.4_: −2.56 ± 0.11 vs. −2.42 ± 0.11 for [^99m^Tc]Tc-**16**). As expected, reference compound [^177^Lu]Lu-**2** showed the highest hydrophilic character (logD_7.4_: -3.80 ± 0.33).

*HSA binding.* Binding to human serum albumin revealed to be strongest for SiOH-BA-bearing peptides (89.1 ± 1.4% for [^99m^Tc]Tc-**12** and 78.1 ± 0.7% for [^99m^Tc]Tc-**15**). All other ^99m^Tc-labeled compounds showed a slightly reduced HSA binding (47.5 ± 1.7% for [^99m^Tc]Tc-**16** up to 53.2 ± 1.8% for [^99m^Tc]Tc-**11**) when compared to reference compounds [^177^Lu]Lu-**2** and [^177^Lu]Lu-**4** (60.8 ± 2.8% and 59.9 ± 1.2%, respectively). 

*Stability in human serum.* The reference compound [^177^Lu]Lu-**4** as well as [^99m^Tc]Tc-**11**, − **16** and − **17** displayed high stability in human serum after incubation for 4 h at 37 °C, with > 95% of intact peptide. [^99m^Tc]Tc-**12** to − **15** revealed serum stabilities < 95%, with [^99m^Tc]Tc-**13** as the least stable peptide (32.1% intact after 4 h at 37 °C). The stability of the second reference compound [^177^Lu]Lu-**2** could not be determined in this assay (Table 1), because the majority of the radioactivity was found to be irreversibly bound to the precipitate after ultrafiltration and thus, the amount of activity in the supernatant was insufficient for analysis via radio-RP-HPLC.

*Stability of radioligands in radiolabeling solution*. Reference compounds [^177^Lu]Lu-**2** and [^177^Lu]Lu-**4** showed > 96% intact peptide when stored for 2 d at 4 °C. Radiolabeling solutions of [^99m^Tc]Tc-**11**, − **12**, − **14**, − **15**, − **16** and − **17** were examined again 5 to 7 h after EOS and revealed > 95% of intact peptide when stored at r.t., which represented an important prerequisite for further use in *in* *vitro* and in vivo studies.

## Discussion

Despite considerable progress in the development of radiolabeled CCK-2R ligands in recent years and the high prevalence of SPECT devices worldwide, ^99m^Tc-labeled compounds targeting the CCK-2R are not established in clinical routine. With the aim to address this shortcoming, we developed novel ^99m^Tc-labeled CCK-2R ligands, based on our previously reported modified peptide sequences CCK-66, rhCCK-18 and rhCCK-84.(Günther et al. 2024; Holzleitner 2023).

The precursor compounds were readily accessible via SPPS after preparation of the SiOH-BA building block and some optimizations required for the N_4_ chelator synthesis. ^177^Lu- and ^99m^Tc-labeling proceeded quantitatively, however, analysis via radio-RP-HPLC and radio-TLC needed to be adjusted for ^99m^Tc-labeled CCK-2R ligands. Typically used reversed-phase C18 columns were found to be unsuitable for analysis, as RCP could not be properly determined due to extensive tailing of the compounds. Thus, a MultoHigh Bio 300–5 C4 column was used for quality control via radio-RP-HPLC. Furthermore, the TLC method established for DOTA-equipped and ^177^Lu-/^67^Ga/^64^Cu-labeled CCK-2R ligands (Günther et al. 2024, 2023; Holzleitner 2023) (0.1 M sodium citrate*1.5 H_2_O on ITLC-SG chromatography paper) for detection of uncomplexed radionuclide proved unsuitable. Likewise, proper quantification of colloidal ^99m^Tc was not possible by the TLC method, established for other [^99m^Tc]Tc-N_4_ chelator-bearing peptides (Günther et al. 2022b; Kunert et al. 2023) (ITLC-SG strips using NH_4_OAc (1 M, aq.)/DMF = 1/1 (*v/v*), Table S1). TLC methods developed for clinically applied ^99m^Tc-labeled tracers for bone scintigraphy, such as [^99m^Tc]Tc-MDP or [^99m^Tc]Tc-HMDP (International atomic energy agency 2008) (MEK on Whatman 1 chromatography paper for quantification of free [^99m^Tc]TcO_4_^−^ and saline on ITLC-SG strips for detection of colloidal ^99m^Tc) could be partially used for the analysis of the novel ^99m^Tc-labeled CCK-2R ligands. But in particular, the formation of ^99m^Tc-colloid could not be properly assessed with the methods described so far, since the peptides did not move or moved only very poorly with the solvent front. Therefore, several stationary and mobile phases in different combinations were examined on their ability to properly identify ^99m^Tc-labeled peptide, uncomplexed [^99m^Tc]TcO_4_^−^ and ^99m^Tc-colloid formation (Table S1). Whatman 1 chromatography paper proved to be most suitable as stationary phase for quantification of free [^99m^Tc]TcO_4_^−^, with methyl ethyl ketone (MEK) as mobile phase (*R*_f_([^99m^Tc]TcO_4_^−^) = 0.9–1.0, *R*_f_(^99m^Tc-labeled peptide) = 0.0–0.1), as well as for the detection of ^99m^Tc-colloid, with MeCN/H_2_O (8/2, 5% TFA) as mobile phase (*R*_f_(^99m^Tc-colloid) = 0.0, *R*_f_(^99m^Tc-labeled peptide) = 0.9–1.0). Using this protocol, RCPs of ≥ 95% could be determined for [^99m^Tc]Tc-**11**, − **12** and − **13**. However, further improvements are needed for the detection of ^99m^Tc-colloid for [^99m^Tc]Tc-**14**, -**15**, -**16** and -**17** (and structurally similar compounds), since for these compounds only maximum RCPs of 94.0%, 93.5%, 91.7% and 87.8% could be reached, respectively. Insufficient movement of the radiopeptides with the solvent front impeded proper quantification of RCP (TLC chromatograms in Sect. 3.2 in the SI) and currently restrains the translational potential of [^99m^Tc]Tc-**14**, -**15**, -**16** and -**17**. It remains to be proven, if better results can be obtained by using NH_4_OAc (1 M, aq.)/MeOH (1/1, *v/v*) on Whatman 3-mm paper strips, as applied for the detection of colloidal ^99m^Tc after the synthesis of [^99m^Tc]Tc-demogastrin 2 and [^99m^Tc]Tc-demotate 1.(Nock et al. 2005; Maina et al. 2002) Alternatively, and based on our TLC results (Table S1), it might also be thinkable that slight modifications of the TLC mobile phase, e.g. MeCN/H_2_O (5/5 up to 10/0, 1–5% TFA) could lead to an improved TLC-based analysis of these compounds. However, radio-RP-HPLCs and MEK-based radio-TLCs clearly showed RCPs ≥ 95% for all ^99m^Tc-labeled CCK-2R ligands that were subjected to in vitro studies.

For ^177^Lu-labeled peptides, 0.1 M sodium citrate*1.5 H_2_O on ITLC-SG chromatography paper was used to detect free ^177^Lu^3+^ ((*R*_f_ (^177^Lu^3+^) = 0.9–1.0, *R*_f_ (^177^Lu-labeled peptide) = 0.0–0.1). The detection of ^177^Lu-colloid was conducted by using MeCN/H_2_O (8/2, 5% TFA) as mobile phase (*R*_f_ (^177^Lu-colloid) = 0.0, *R*_f_ (^177^Lu-labeled peptide) = 0.9–1.0) and Whatman 1 strips for [^177^Lu]Lu-DOTA-CCK-66, while Whatman 31ET strips showed best results for [^177^Lu]Lu-DOTA-PP-F11N. Radio-TLC and radio-RP-HPLC analyses confirmed RCPs > 95% for all ^177^Lu-labeled CCK-2R ligands used for in vitro studies.

The solutions of ^177^Lu- and ^99m^Tc-labeled peptides could be used for in vivo experiments within a time frame of 5 to 7 h after EOS with no further adjustment of the pH, since RCP was > 95% and they already provided pH values of 5–7 after EOS (pH range for i.v. injection in mice: 5–8).

For a valid assessment of the novel ^99m^Tc-labeled CCK-2R ligands, their in vitro data needed to be compared with the data obtained for the ^177^Lu-labeled reference compounds and also interpreted with regard to previously published data (i.e. IC_50,inverse_ data of this study vs. IC_50_ data of previous studies). Data published by Holzleitner *et al*. and Günther et al. (obtained under very similar experimental conditions) showed IC_50_ values of 12.8 ± 2.8 nM (Holzleitner et al. 2022) for ^nat^Lu-**2** and 5.12 ± 0.51 nM (Günther et al. 2024) for ^nat^Lu-**4**, corresponding to IC_50,inverse_ values of 28.2 ± 7.9 nM and 40.3 ± 7.1 nM, respectively, obtained in this study. In consequence, IC_50,inverse_ values of > 30 nM can be equated to IC_50_ values of < 10 nM. Since both reference compounds were already clinically applied, we considered ^99m^Tc-labeled compounds with IC_50,inverse_ values > 28 nM as promising candidates. Moreover, internalization, lipophilicity, HSA binding and stability in human serum were taken into account when assessing which compounds should be subjected to in vivo studies. [^99m^Tc]Tc-**11** provided the highest internalization value of all ligands (223.8 ± 0.1% compared to the external reference [^177^Lu]Lu-**2**), which was 1.5-fold higher than the internalization of [^177^Lu]Lu-**4**. In addition, [^99m^Tc]Tc-**11** showed a favorable affinity (IC_50,inverse_ = 27.9 ± 5.7 nM), high stability in human serum (> 95%), similar HSA binding and only a slightly higher lipophilicity than [^177^Lu]Lu-**4**. The same applied to [^99m^Tc]Tc-**16** and [^99m^Tc]Tc-**17**, but with lower internalization values, particularly observed for [^99m^Tc]Tc-**17** (102.4 ± 0.1%). SiOH-bearing compounds [^99m^Tc]Tc-**12** and [^99m^Tc]Tc-**15** generally showed higher lipophilicity and HSA binding, but also lower stability in human serum (87.0 - 93.0% after 4 h at 37 °C).

Certain conclusions could also be drawn by comparison to other, ^99m^Tc-labeled CCK-2R ligands (Fig. 2), such as [^99m^Tc]Tc-demogastrin 2 (N_4_ chelator-bearing, clinically most advanced ^99m^Tc-labeled CCK-2R ligand) or [^99m^Tc]Tc-EDDA/HYNIC-MGS5 (favorable preclinical data, stabilized towards enzymatic degradation), although in vitro experiments were set up differently. [^99 m/99g^Tc]Tc-demogastrin 2 displayed high CCK-2R affinity (dissociation constant K_d_ of 1.02 ± 0.07 nM obtained by receptor binding studies on AR42J cell membranes) and high receptor-mediated internalization in AR42J cells (~ 80% of applied activity, 1 h incubation at 37 °C, not corrected for unspecific binding or normalized to an external reference).(Nock et al. 2005) No trace of intact [^99 m/99g^Tc]Tc-demogastrin 2 could be detected in murine urine at 30 min p.i. (healthy Swiss albino mice), while 77.8 ± 2.3% intact [^177^Lu]Lu-DOTA-CCK-66 was found in murine urine at the same time point after injection (healthy CB17-SCID mice).(Günther et al. 2024; Nock et al. 2005) This in turn suggests for a high metabolic stability of [^99m^Tc]Tc-N_4_-CCK-66, since it is based on the peptide structure of DOTA-CCK-66 with only the chelator moiety differing (Fig. 3).

[^99m^Tc]Tc-EDDA/HYNIC-MGS5 revealed a K_d_ value of 13.7 ± 1.1 nM and an internalization of ~ 38% in A431‐CCK2R cells after incubation for 1 h (not corrected for unspecific binding and not normalized to an external reference). Protein binding in human serum was assessed by Sephadex G-50 size-exclusion chromatography with 36.8 ± 0.1% for [^99m^Tc]Tc‐HYNIC‐MGS5 after 24 h incubation and thus lower than any HSA binding observed for ^177^Lu- or ^99m^Tc-labeled ligands in this study (47.5 ± 1.7% up to 89.1 ± 1.4%), determined by ultrafiltration. Besides, [^99m^Tc]Tc‐HYNIC‐MGS5 provided a logD_7.4_ value of -2.91 ± 0.06 and high stability in human serum (> 95% intact after 24 h, no incubation temperature given). (Klingler et al. 2019b) Thus, some of our novel ^99m^Tc-labeled CCK-2R ligands may show slightly decelerated clearance from the blood compared to [^99m^Tc]Tc-EDDA/HYNIC-MGS5 and all of them offer the possibility of coligand-free ^99m^Tc-labeling by using the open-chain N_4_ chelator.

For [^99m^Tc]Tc-DGA1, there was not enough in in vitro data available to allow for a valid comparison. DGA1 exhibited an IC_50_ value of 1.62 ± 0.17 nM (displacement of [^125^I]I-Tyr^12^,Leu^15^]gastrin from CCK_2i4sv_R sites on HEK293-CCK_2i4sv_R cell membranes). Hence, these data do not accurately express the affinity of [^99m^Tc]Tc-DGA1, but of the unlabeled precursor. At 1 h of incubation at 37 °C, 10.0 ± 1.1% of the applied [^99m^Tc]Tc-DGA1 activity was internalized into HEK293-CCK_2i4sv_R cells (not corrected for unspecific binding and not normalized to an external reference). No data for lipophilicity, HSA binding or stability in human serum were collected by Kaloudi et al.(2020) [^99m^Tc]Tc-DGA1 demonstrated high in vivo stability, with 97.0 ± 0.5% intact in the blood of Swiss Albino mice at 5 min p.i. However, no probes were taken at 30 min p.i. and hence, a comparison to peptide scaffolds used in this study (e.g. [^177^Lu]Lu-DOTA-CCK-66: 78.5 ± 3.1% intact in murine serum at 30 min p.i.) was not possible. 

Our approach not only focused on the development of CCK-2R radioligands with superior pharmacokinetics in comparison to other ligands such as [^99m^Tc]Tc-DGA1, [^99m^Tc]Tc-MGS5 or [^99m^Tc]Tc-demogastrin 2, but we also intended to provide compounds for which radiolabeling can be easily implemented into clinical practice. Following previously conducted studies, we decided to use the open-chain N_4_ chelator, which enables fast, kit-like, efficient and coligand-free ^99m^Tc-labeling with high in vivo stability, as shown for multiple clinically applied radioligands (Mather et al. 2014; Rinscheid et al. 2024; Konrad et al. 2023; Decristoforo et al. 2003; Novak et al. 2023) Therefore, we are convinced that our novel CCK-2R-targeted compounds offer great potential for successful transfer into human application, depending on which ligand proves to be the most suitable based on preclinical in vivo examinations.

**Fig. 5:**
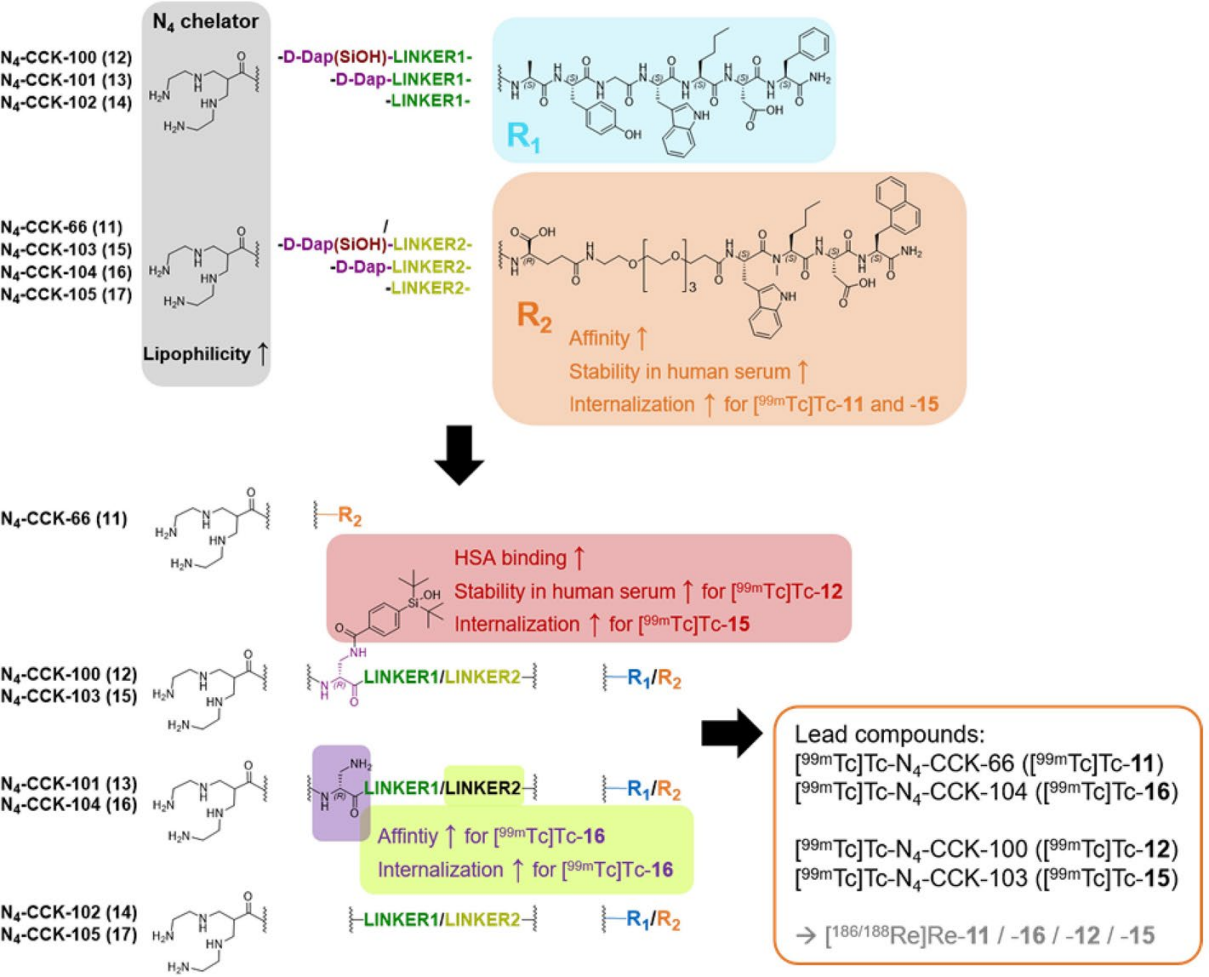
Graphical depiction of CCK-2R ligands investigated in this study, highlighting specific structural modifications to explicitly visualize the structure–activity-pharmacokinetics relationships underlying the design strategy and to illustrate the rationale for selecting specific candidates as lead compounds

Overall, [^99m^Tc]Tc-N_4_-CCK-66 ([^99m^Tc]Tc-**11**) and [^99m^Tc]Tc-N_4_-CCK-104 ([^99m^Tc]Tc-**16**) showed the best performance and thus, are recommended for further evaluation in in vivo studies. Imaging and biodistribution time points at 1 h and 4 h p.i. are suggested to provide the most valuable data for comparative preclinical analyses. Furthermore, in vivo evaluation of SiOH-bearing agents N_4_-CCK-100 and N_4_-CCK-103 in their ^99m^Tc-labeled form ([^99m^Tc]Tc-**12** and [^99m^Tc]Tc-**15**) at later time points (12–24 h p.i.) could give valuable insights into their eligibility as agents for radioguided surgery. A future evaluation of ^186/188^Re-labeled derivatives in vivo might also be conceivable in order to investigate their potential as radiotheranostic agents (Fig. 5).

Kit-like preparations for all these peptides were not yet conducted, since in vivo studies are intended to first provide insight into which compounds would be most suitable for administration to patients. However, we presume that a freeze-dried formulation (i.e. precursor + Na_2_HPO_4_ + disodium citrate sesquihydrate + SnCl_2_ + sodium-L-ascorbate), stored under an inert atmosphere (Nitrogen or Argon) and at sub-zero temperatures can be readily used for addition of ^99m^Tc-generator eluate ([^99m^Tc]TcO_4_^−^ in isotonic saline) and successful ^99m^Tc-labeling upon heating of the reaction mixture for 15 min to 95 °C. Such a kit-like approach has already been published by our group for ^99m^Tc-labeled N_4_ chelator-equipped compounds targeting the C–X–C motif chemokine receptor 4. (Konrad et al. 2023) Alternatively, the already established labeling procedure could also be used for patient applications following some slight adjustments, as our group has already demonstrated for ^99m^Tc-labeled N_4_ chelator-equipped peptides, targeting the gastrin-releasing peptide receptor (Günther et al. 2022b) Considering a potential future clinical translation of compounds [^99m^Tc]Tc-**15** and [^99m^Tc]Tc-**16**, TLC-based methods for RCP determination and in particular for assessment of ^99m^Tc-colloid formation need to be further improved in order to comply with good radiopharmacy practice.

## Conclusions

In sum, several novel ^99m^Tc-labeled CCK-2R ligands revealed favorable in vitro characteristics in this study, which emphasizes their potential as SPECT tracers for improved ^99m^Tc-based diagnosis of CCK-2R-overexpressing malignancies. In particular, [^99m^Tc]Tc-N_4_-CCK-66 ([^99m^Tc]Tc-**11**) and [^99m^Tc]Tc-N_4_-CCK-104 ([^99m^Tc]Tc-**16**) are highly recommended for further evaluation in in vivo studies to examine their potential as ^99m^Tc-based SPECT agents. In vivo evaluation of the SiOH-bearing agents N_4_-CCK-100 (**12**) and N_4_-CCK-103 (**15**) may provide valuable insights into their eligibility as agents for radioguided surgery, given their higher binding to human serum albumin and thus, projected decelerated clearance kinetics. A possible radiotheranostic use of all these N_4_-conjugated compounds could be explored in future in vivo studies using their ^186/188^Re-labeled derivatives.

## Supplementary Information

Supplementary Material 1.

## Data Availability

The datasets supporting the conclusions of this article are included within this article and its additional file.

## Abbreviations

A_m_: Molar activity
aq.: Aqueous
CCK: Cholecystokinin
CCK-2R: Cholecystokinin-2 receptor
Dap: 2,3-Diaminopropionic acid
DMF: Dimethylformamide
DMSO: Dimethyl sulfoxide
DOTA: 1,4,7,10-Tetraazacyclododecane-1,4,7,10-tetraacetic acid
EDDA: Ethylenediamine-*N,N*′-diacetic acid
ESI: Electrospray ionization
Fmoc: Fluorenylmethyloxycarbonyl
HSA: Human serum albumin
HYNIC: 6-Hydrazinonicotinamide
Hyp: L-hydroxyproline
IC_50,inverse_: Inverse half maximal inhibitory concentration
ITLC-SG: Instant thin-layer chromatography paper impregnated with silica gel
K_d_: Dissociation constant
MEK: Methyl ethyl ketone
MS: Mass spectrometry
MTC: Medullary thyroid carcinoma
N_4_: Tetraamine
NMR: Nuclear magnetic resonance
PBS: Phosphate-buffered saline
PEG: Polyethylene glycol
PET: Positron emission tomography
p.i.: *post injectionem*
RCP: Radiochemical purity
RCY: Radiochemical yield
rh: Radiohybrid
RP-HPLC: Reversed-phase high-performance liquid chromatography
r.t.: Room temperature
SCID: Severe combined immunodeficiency
SI: Supporting information
SiFA: Silicon-based fluoride acceptor
SiFA-BA: Silicon-based fluoride acceptor bound to benzoic acid; in this study SiFA-BA refers to 4-(di-tert-butylfluorosilyl)benzoic acid
SiOH: Hydrolyzed silicon-based fluoride acceptor
SiOH-BA: 4-(Di-*tert*-butyl(hydroxy)silyl)benzoic acid
SPECT: Single-photon emission computed tomography
SPPS: Solid-phase peptide synthesis
TFA: Trifluoroacetic acid
vs.: Versus
