# Supplementary material for: Synthesis and in vitro evaluation of pharmacokinetically optimized 99mTc-labeled cholecystokinin-2 receptor-targeted peptides for improved SPECT imaging
Source: EJNMMI Radiopharm Chem. 2026 Jul 20;11:48. doi: 10.1186/s41181-026-00477-5 (PMC13388586; doi:10.1186/s41181-026-00477-5)
Supplement: Supplementary file 1 — Supplementary Material 1. [file 41181_2026_477_MOESM1_ESM.docx]

**Synthesis and *in vitro* evaluation of pharmacokinetically optimized ^99m^Tc-labeled cholecystokinin‑2 receptor‑targeted peptides for improved SPECT imaging**

**- Supplementary Information -**

Veronika Felber ^1,†,^ *, Nadine Holzleitner ^2^, Thomas Günther ^3^

^1^ TUM School of Natural Sciences, Department of Chemistry, Chair of Pharmaceutical Radiochemistry, Technical University of Munich, Garching, Germany

^2^ Department of Nuclear Medicine, TUM University Hospital and Central Institute for Translational Cancer Research, (TranslaTUM), School of Medicine, Technical University of Munich, Munich, Germany

^3^ Molecular Imaging Program at Stanford (MIPS), Department of Radiology, School of Medicine, Stanford University, Stanford, CA, USA

^†^ First author

* Corresponding author

***First and corresponding author:**

Veronika Felber, PhD

veronika.felber@univie.ac.at

ORCID-ID: 0000-0003-0072-9769

**Co authors:**

Nadine Holzleitner, PhD

[nadine.holzleitner@tum.de](mailto:nadine.holzleitner@tum.de)

ORCID-ID: 0000-0001-8258-3526

Thomas Günther, PhD

[tgunther@stanford.edu](mailto:tgunther@stanford.edu)

ORCID-ID: [0000-0002-7412-0297](https://orcid.org/0000-0002-7412-0297?lang=en)

**Address of first author:**

Technical University of Munich

Chair of Pharmaceutical Radiochemistry

Walther-Meissner-Str. 3, 85748 Garching, GERMANY

Phone: +49.89.289.12203; Fax: +49.89.289.12204

1. **MATERIALS**

The Fmoc- (9-fluorenylmethoxycarbonyl-) and all other protected amino acid analogs were purchased from Bachem Inc. (Bubendorf, Switzerland), Merck KGaA (Darmstadt, Germany), Carbolution (St. Ingbert, Germany) or Iris Biotech GmbH (Marktredwitz, Germany). The *H*-Rink amide ChemMatrix^®^ resin (35-100 mesh particle size, 0.4 - 0.6 mmol/g loading) was purchased from Merck KGaA (Darmstadt, Germany). Fmoc‑Rink amide ProTide resin (LL, 0.15 ‑ 0.25 mmol/g) was purchased from CEM GmbH (Kamp-Lintfort, Germany). CheMatech (Dijon, France) provided the DOTA‑NHS ester and DOTA(*t*Bu)_3_.

All necessary solvents and other organic reagents were purchased from either, Alfa Aesar^TM^ (Karlsruhe, Germany), Merck KGaA (Darmstadt, Germany), VWR International GmbH (Bruchsal, Germany) or CEM GmbH (Kamp-Lintfort, Germany). Unless otherwise stated, H_2_O was taken from a Barnstead™ MicroPure™ System (Thermo Fisher Scientific, Darmstadt, Germany) connected to an upstream DI 1500 ion exchange cartridge (Thermo Fisher Scientific, Darmstadt, Germany).

Manual solid-phase peptide synthesis (SPPS) was carried out by using a Scilogex MX-RL-E Analog Rotisserie Tube Rotator (Scilogex^®^, Rocky Hill, CT, USA) and syringes equipped with plunger and frit (25 μm pore size) were obtained from Carl Roth, GmbH & Co. KG (Karlsruhe, Germany).

Automated solid-phase peptide synthesis (SPPS) was carried out by using the Liberty Blue™ 2.0 microwave peptide synthesizer (CEM GmbH, Kamp-Lintfort, Germany) with *in situ* fiber optic temperature control. The system was operated by the Liberty Blue^TM^ application software.

Analytical and preparative reversed-phase high‑performance liquid chromatography (RP‑HPLC) were performed using Shimadzu gradient systems (Shimadzu Deutschland GmbH, Neufahrn, Germany), each equipped with a SPD-20A UV/Vis detector (220 nm, 254 nm). All systems were operated by the LabSolutions software.

As different eluents and flow rates have been used for several compounds, the used methods are cited in the text and described as follows:

Method A (*analytical & radio-RP-HPLC*): solvent A = water + 0.1% TFA, solvent B = acetonitrile + 2% water + 0.1% TFA

Method B (*preparative RP-HPLC*): solvent A = water + 0.1% TFA, solvent B = acetonitrile + 5% water + 0.1% TFA

Method C (*flash chromatography*): solvent A = water + 0.1% TFA, solvent B: acetonitrile + 0.1% TFA

Analytical RP-HPLC was performed either with a Nucleosil 100-5 C18 (5 μm, 125 mm x 4.6 mm) or a MultoKrom 100-5 C18 (150 × 4.6 mm) column (both purchased from CS Chromatographie Service GmbH, Langerwehe, Germany) applying different linear solvent gradients (Method A) and a constant flow rate of 1 mL/min. Both, specific gradients and the corresponding retention times t*_R_* as well as the capacity factor *k* are cited in the text.

Preparative RP-HPLC was performed using different MultoKrom 100-5 C18 (5 µm, 150 × 10 mm or 250 × 20 mm) columns (CS Chromatographie Service GmbH, Langerwehe, Germany) at different constant flow rates of 5 or 10 mL/min.

Flash chromatography was performed on a Biotage gradient system (Biotage Europe, Uppsala, Sweden), using Biotage^®^ Sfär C18 D cartridges (Duo 100 Å, 30 µm, 12 g). The compounds were eluted applying different solvent gradients (Method C) and a constant flow rate of 12 mL/min.

Analytical radio‑RP-HPLC for ^99m^Tc-labeled compounds was performed using a MultoHigh Bio 300-5 C4 (5 µm, 150 × 4.6 mm) column (CS Chromatographie Service GmbH, Langerwehe, Germany). For ^177^Lu-labeled compounds, a MultoKrom 100-5 C18 (150 × 4.6 mm) column (CS Chromatographie Service GmbH, Langerwehe, Germany) was used. For radioactivity detection, the outlet of the UV detector was connected to a HERM LB 500 NaI detector (Berthold Technologies, Bad Wildbad, Germany) and a FlowStar^2^ LB 514 detector (Berthold Technologies, Bad Wildbad, Germany).

Radio‑thin‑layer chromatography (radio-TLC) for ^99m^Tc-labeled compounds was performed on Whatman 1 chromatography paper (Cytiva Europe GmbH, Freiburg im Breisgau, Germany) using methyl ethyl ketone (MEK) as mobile phase for quantification of free [^99m^Tc][TcO_4_]^−^ and MeCN/H_2_O (80/20, + 5% TFA) as mobile phase for detection of colloidal technetium-99m (^99m^TcO_2_). For ^177^Lu-labeled compounds, instant thin-layer chromatography paper impregnated with silica gel (ITLC‑SG, Agilent Technologies Inc., Folsom, United States) with disodium citrate sesquihydrate (0.1 M, aq.) as mobile phase was used for detection of free ^177^Lu^3+^. Detection of colloidal lutetium‑177 was performed either on Whatman 1 (for [^177^Lu]Lu-DOTA‑CCK‑66) or Whatman 31 ET chromatography paper (for [^177^Lu]Lu-DOTA‑PP‑F11N) using MeCN/H_2_O (80/20, + 5% TFA) as mobile phase. Radio‑TLC strips were analyzed by using a Scan-RAM^TM^ detector operated by the Laura^TM^ software (LabLogic Systems Ltd., Broomhill, Sheffield, United Kingdom).

Activity measurements of the respective probes obtained from competitive binding assays were measured by a 2480 Wizard^2^ automatic ɣ-counter (PerkinElmer, Waltham, USA) and evaluated with GraphPad PRISM 7.

Mass spectra were acquired with an Advion expression^L^ compact mass spectrometer (Advion Ltd., Harlow, UK) with electrospray ionization (positive ion mode) and an orthogonal ion sampling from the heated capillary. The system was operated by the Mass Express software and spectra were processed using the Data Express software.

All ^1^H-, ^13^C-, ^19^F- and ^19^F{^1^H}-NMR spectra were measured at room temperature in either DMSO-d_6_ or CDCl_3_ on a Bruker (Rheinstetten, Germany) instrument (AVHD400, Avance-III). Chemical shifts (δ) are reported in parts per million (ppm) and calibrated on the residual solvent signal (DMSO-d_6_: 2.50 ppm for ^1^H and 39.5 ppm for ^13^C, CDCl_3_: 7.26 ppm for ^1^H and 77.0 ppm for ^13^C). Multiplicities are described as follows: s = singlet, d = doublet, t = triplet, q = quartet, br = broad singlet m = multiplet.

For lyophilization, an Alpha 1-2 lyophilizer (Martin Christ Gefriertrocknungsanlagen GmbH, Osterode am Harz, Germany) connected to an Edwards nXDS10i oil-free scroll pump (Edwards GmbH, Feldkirchen, Germany) was used.

For determination of the lipophilicity (logD_7.4_) and binding to human serum albumin (HSA) a Heraeus Pico 17 microcentrifuge and a Heraeus Megafuge 16 (both Thermo Fisher Scientific Inc., Waltham, United States) were used, respectively.

A Heraeus Pico 17 microcentrifuge (Thermo Fisher Scientific Inc., Waltham, United States) was used to precipitate residual plasma proteins in human serum samples.

1. **METHODS**
   1. Synthesis of building blocks for SPPS
      1. 4-(Di-*tert*-butyl(hydroxy)silyl)benzoic acid (SiOH-BA)

4-(Di-*tert*-butyl(hydroxy)silyl)benzoic acid (SiOH-BA, **S1**) was obtained by base-catalyzed hydrolysis of 4-(di-*tert*-butylfluorosilyl)benzoic acid (SiFA-BA) for which the synthesis has already been described previously.^(1, 2)^ To a stirring solution of SiFA-BA (105 mg, 0.37 mmol, 1.00 eq.) in 3.69 mL DMF, 0.93 mL of a 2 M KOH_(aq.)_ solution (1.86 mmol, 5.00 eq.) was added and stirred for 2 h at room temperature. The reaction mixture was acidified (pH ~ 4) by addition of 1.6 mL 1 M HCl_(aq.)_ (1.60 mmol, 4.32 eq.), diluted with 30 mL H_2_O and extracted with diethyl ether (3 × 30 mL). The combined organic layers were dried over MgSO_4_, filtered and the solvent was removed under reduced pressure. Purification by preparative RP-HPLC (20 - 80% B in 20 min, Method B, 10 mL/min) afforded 36.1 mg (34.6%) of pure product **S1** as colorless powder.

RP-HPLC (40-80% B in 20 min, Method B, 10 mL/min): t*_R_* = 13.8 min; *k* = 1.76. Calculated monoisotopic mass (C_15_H_24_O_3_Si): 280.15, found (*Figure S3*): m/z = 144.9 [M(Fragment 1 (SiOH‑BA))+Na^+^]^+^, 185.9 [M_2_(**S1**)+3H^+^]^3+^, 304.3 [M(**S1**)+Na^+^]^+^.

^1^H-NMR (400 MHz, CDCl_3_): δ [ppm] = 8.08 (d, ^3^*J*_H‑H_ = 8.2 Hz, 2H, H(1, 1‘)), 7.79 (d, ^3^*J*_H‑H_ = 8.2 Hz, 2H, H(2, 2‘)), 1.05 (s, 18H, H(3, 3‘, 3‘‘, 4, 4‘, 4‘‘)).

^19^F-NMR (376 MHz, CDCl_3_): no signal at -188.6 ppm (*Figure S2*)

^19^F{^1^H}-NMR (376 MHz, CDCl_3_): no signal at -188.6 ppm (*Figure S2*)

- - 1. *N,N',N'',N'''*-tetrakis(*tert*-butyloxycarbonyl)-6-carboxy-1,4,8,11-tetraazaundecane (N_4_Boc_4_)

N_4_Boc_4_ (**S2**) was synthesized in analogy to a previously published procedure by Günther *et al*.^(3)^ with some modifications to increase the chemical yield. *N*‑Boc-ethylenediamine (950 µL, 6.00 mmol, 4.00 eq.) was slowly added to a solution of 3-bromo-2-(bromomethyl)propionic acid (369 mg, 1.50 mmol, 1.00 eq.) in 37.5 mL THF (25 mL/mmol) and stirred for at least 24 h at room temperature. The solvent was removed under reduced pressure at room temperature and the residue was dissolved in 30 mL 2‑butanone/acetone (1/2, v/v, 20 mL/mmol). At 0 °C, 1024 µL triethylamine (7.39 mmol, 4.93 eq.) were added to reach a pH of ~11. After warming to 25 °C, di-*tert*-butyl dicarbonate (1.38 mL, 6.00 mmol, 4.00 eq.) was added and the mixture was stirred at 40 °C for 24 h. The reaction was terminated by adding 10 - 20 mL H_2_O. 2‑Butanone as well as acetone were removed *in vacuo* and MeCN was added to the suspension until the crude product dissolved well and it was purified via flash chromatography (35 ‑ 95% B in 15 min, Method C), which afforded 701 mg (77.3%) of product **S2** as a slightly reddish solid.

RP-HPLC (10 ‑ 90% B in 15 min, Method A, 1 mL/min): t*_R_* = 11.3 min; *k* = 6.5. Calculated monoisotopic mass (C_28_H_52_N_4_O_10_): 604.4; found (*Figure S5*): m/z = 505.0 [M(**S2**) - Boc + H^+^]^+^, 605.0 [M(**S2**) + H^+^]^+^, 1230.7 [M_2_(**S2**) + Na^+^]^+^.

^1^H-NMR (400 MHz, CDCl_3_), δ [ppm] = 4.37 (s, 3H, H(1, 2, 3)), 3.51 – 3.14 (m, 12H, H(4, 5, 6, 7, 8, 9)), 3.08 (s, 1H, H(10)), 1.41 (*virt*. d, 36H, H(11, 11’, 11’’, 12, 12’, 12’’, 13, 13’, 13’’, 14, 14’, 14’’); Residual solvent signals at 4.78 ppm (H_2_O) and 1.98 ppm (MeCN).

^13^C-NMR (101 MHz, CDCl_3_), δ [ppm] = 175.93 (s, 1C, C(1)), 156.57 (s, 2C, C(2, 3)), 156.18 (s, 2C, C(4, 5)), 80.73 (s, 2H, C(6, 7)), 79.42 (s, 2C, C(8, 9)), 48.32 (s, 4C, C(10, 11, 12, 13), 39.48 (s, 3C, C(14, 15, 16), 28.48 ‑ 28.41 (m, 12C, C(17, 17’, 17’’, 18, 18’, 18’’, 19, 19’, 19’’, 20, 20’, 20’’); Residual solvent signals at 117.79 ppm and 1.94 ppm (MeCN).

- 1. Peptide synthesis

General remarks on manual solid-phase peptide synthesis

The used equivalents of the reactants for the solid phase synthesis refer to the calculated load after attaching the first amino acid onto the resin. Specific loads are cited in the text. Prior to any reaction, dry resin was swollen in NMP for at least 30 min and then filtered. Unless otherwise indicated, the resin was washed with DMF (6×) after each reaction step. For storage, the resin was washed with DMF (3×) and DCM (3×) and dried in a desiccator.

General procedure for loading the first amino acid onto Rink amide ChemMatrix^®^ resin and on-resin peptide bond formation (GP1)

Rink amide ChemMatrix^®^ resin (average load: 0.50 mmol/g) was obtained as the free *N*-terminal form and hence, loading of the first amino acid and further elongation steps were performed by the same standard procedure. The (first) amino acid (2.00 eq.), TBTU (2.00 eq.) and HOAt (2.00 eq.) were dissolved in DMF (~ 10 mL/g resin) and preactivated by addition of DIPEA (4.50 eq.) for five minutes, prior to incubation with the resin. Unless otherwise noted, the solution was added to the resin and shaken for 2 h at room temperature. Slightly different equivalents were used for coupling of N_4_Boc_4_ and DOTA(*t*Bu)_3_ (both 1.50 eq.), DOTA‑NHS ester (1.10 eq.) as well as SiOH-BA (1.00 eq.). Moreover, for coupling of DOTA-NHS the coupling reagents TBTU and HOAt were not required, but only DIPEA (8.50 eq.). Coupling of DOTA chelator derivatives was conducted overnight (16 ‑ 24 h). Occasionally, the pH value had to be adjusted to 9 ‑ 10 by addition of further DIPEA. *Sym*‑collidine (9.00 eq.) was used as base for coupling of Fmoc‑L‑Dap(Dde)-OH and SiOH‑BA instead of DIPEA.

General procedure for the on-resin Fmoc-removal (GP2)

The resin was shaken 1 × 5 min and subsequently 1 × 15 min in 20% piperidine in DMF (v/v) to remove the Fmoc-protective group and afterwards washed with DMF (7×).

General procedures for the on-resin Dde-removal (GP3 & GP4)

GP3: If no Fmoc-group was present in the resin‑bound peptide, the resin was treated with 2%

hydrazine in DMF (10 mL) for 20 min and afterwards washed with DMF (7×).

GP4: If an Fmoc-group was present in the resin‑bound peptide, the resin was treated with a solution

of imidazole (0.46 g/g resin) and hydroxylamine hydrochloride (0.63 g/g resin) in NMP/DMF

(5/1, v/v) for 2 × 3 h. Afterwards, the resin was washed with DMF (7×).

General procedures for monitoring the reaction progress (GP5)

For a test cleavage with TFA (GP5) a small aliquot of the resin was taken and treated with 100 μL of TFA for 15 min at r.t. in an Eppendorf tube. The respective solution (without beads!) was transferred into another Eppendorf tube and the solvent was evaporated under a stream of nitrogen. The residue was dissolved in a mixture of H_2_O and MeCN (1/1, *v/v*), now ready for RP‑HPLC analysis.

Cleaving the peptide off the resin with simultaneous removal of all acid-labile protective groups (GP6)

The resin was treated with TFA/TIPS/DCM (95/2.5/2.5, 10.0 mL) twice for 30 min at r.t. and washed with DCM afterwards (3×). The solvent was evaporated under N_2_ flow and after lyophilization the crude product was obtained.

General remarks on automated solid-phase peptide synthesis (Liberty Blue™ 2.0 microwave peptide synthesizer)

Peptide syntheses were conducted on a 0.05 ‑ 0.25 mmol scale and amounts of Fmoc‑protected amino acids, calculated by the Liberty Blue^TM^ application software, were diluted in DMF to reach a final concentration of 0.2 M. Thus, amino acid equivalents ranged from 4 (0.25 mmol scale) to 6 (0.05 mmol scale). As reaction solvent DMF was used and for removal of Fmoc-protective groups a solution of 20% piperidine in DMF (*v/v*) was freshly prepared. 0.5 M DIC in DMF (30 mL) served as *activator* and a mixture of 0.5 M Oxyma and 0.1 M DIPEA in DMF (20 mL) was used as *activator base*. ‘Double coupling’ was performed for *N*-methylated amino acids and the following amino acid in order to avoid incomplete peptide elongation.

Full-length peptides were not assembled *via* automated peptide synthesis, but peptide fragments. Chelator attachment (occasionally required prolonged reaction times) as well as coupling of Fmoc‑D‑Dap(Dde)-OH and SiOH-BA (for both *sym*-collidine needs to be used as base) was conducted manually. Hence, GP3 to GP6 were always performed manually and do not have an automated equivalent.

Structures and analytical data of DOTA-PP-F11N (**2**), ^nat^Lu-**2**, DOTA-CCK-66 (**4**), ^nat^Lu‑**4**, N_4_‑CCK-66 (**11**), -100 until -105 (**12** - **17**) are given in the Appendix (*Section 3.1*). Representative radio‑RP‑HPLC chromatograms as well as radio‑TLCs are also displayed in the Appendix (*Section 3.2*).

- 1. *In vitro* experiments

*Cell Culture.* CCK‑2R‑expressing AR42J rat pancreatic cancer cells (CLS GmbH, Eppelheim, Germany) were cultivated in monolayers in CELLSTAR^®^ cell culture flasks purchased from Greiner Bio-One GmbH (Frickenhausen, Germany) at 37 ℃ in a humidified atmosphere (5% CO_2_) using a HERAcell 150i-Incubator (Thermo Fisher Scientific Inc., Waltham, United States). RPMI 1640 medium (Gibco^TM^ REF‑number: 21875-091; Fisher Scientific GmbH, Schwerte, Germany) supplemented with 10% fetal bovine serum (FBS), 5 mL non-essential amino acids (100×) and additional L-glutamine (5 mM) was used as culture medium. A Dulbecco’s phosphate‑buffered saline (PBS) (Merck KGaA, Darmstadt, Germany) solution with 0.1% EDTA (*v/v*) was applied to detach the cells for cell passaging. One day (24 ± 2 h) prior to affinity determinations (IC_50,inverse_) or internalization experiments, the cultivated AR42J cells were harvested using 0.1% EDTA (*v/v*) in PBS and centrifuged at 1300 rpm (ca. 190 × g) for 3 min at room temperature (Heraeus Megafuge 16, Thermo Fisher Scientific Inc., Waltham, United States). After centrifugation, the supernatant was disposed and the cell pellet was resuspended in culture medium. Cells were counted with a Neubauer hemocytometer (Paul Marienfeld GmbH & Co. KG, Lauda‑Königshofen, Germany) and seeded in 24-well plates (Greiner Bio‑One GmbH, Frickenhausen, Germany). IC_50,inverse_ values were determined by seeding 2.0 × 10^5^ cells/well into 24‑well plates, whereas internalization was assessed by seeding 3.0 × 10^5^ cells/well into poly‑L‑lysine (PLL)-coated 24‑well plates (Greiner Bio‑One GmbH, Frickenhausen, Germany). Cells were incubated for 24 ± 2 h in 1 mL of culture medium at 37 °C in a humidified atmosphere (5% CO_2_). All operations under sterile conditions were accomplished using a MSC-Advantage biological safety cabinet (Thermo Fisher Scientific Inc., Waltham, United States).

*Affinity determinations (IC_50,inverse_).* After removal of the culture medium, each well was washed with 500 μL PBS. For the cell-based assay, 200 μL of assay medium (= culture medium containing 5% (*w/v*) bovine serum albumin (BSA; Merck KGaA, Darmstadt, Germany)) was added to each well. 25 µL/well of either assay medium (= control) or of solutions, containing the non‑radiolabeled standard competitor ^nat^Lu-DOTA-PP-F11N in increasing concentrations (10^‑10^- 10^-4^ M in Hank’s buffered salt solution (HBSS) containing 1% (*w/v*) BSA; Merck KGaA, Darmstadt, Germany) were added, followed by the addition of 25 µL of the radiolabeled peptide of interest (^99m^Tc‑ or ^177^Lu‑labeled CCK-2R ligand, 12 nM in assay medium) to each well. Experiments were carried out in triplicates for each concentration. The final concentrations of non‑radiolabeled standard competitor ranged from 10^-11^- 10^-5^ M/well and the final radioligand concentration was 1.2 nM/well in all competitive binding assays. The cells were incubated for 3 h at 37 °C. Incubation was terminated by removal of the supernatant. The cells were washed with 300 µL of PBS and the wash medium was combined with the respective supernatant. This fraction represents the amount of free radioligand. The cells were lysed by addition of 300 µL of 1 M aqueous NaOH. After 10 - 15 min, the lysate of each well was transferred to the respective vial as well as 300 µL of 1 M NaOH used for rinsing the well. Quantification of the amount of free and bound activity was performed in a γ‑counter. The corresponding IC_50,inverse_ values were calculated using the GraphPad PRISM7 software (GraphPad Software Inc., La Jolla, United States).

*Internalization studies.* The culture medium was removed, and each well was washed with fresh culture medium (300 μL). Afterwards, 200 μL of culture medium and either 25 μL of culture medium for internalization studies (n = 3) or 25 μL of ^nat^Lu-DOTA-PP-F11N (100 μM in culture medium, final concentration: 10 µM/well) for blocking CCK‑2R‑specific binding and uptake (n = 3) was added. To each well 25 μL of the ^99m^Tc‑ or ^177^Lu‑labeled peptide (12 nM in culture medium, n = 6) was added and incubated for 1 h at 37 °C in a humidified atmosphere (5% CO_2_). The final concentration of the ^99m^Tc‑ and ^177^Lu‑labeled ligands was 1.2 nM/well in all internalization assays. After incubation, the cells were put on ice (ca. 4 °C) for at least 1 min to stop internalization kinetics and the supernatant was collected. The cells were washed with 300 µL of ice-cold culture medium and the wash medium was combined with the respective supernatant. This fraction represents the amount of free radioligand. 300 µL of ice-cold glycine buffer (aq., 1.00 M, pH = 2.2, adjusted with HCl) were added and the cells were incubated for 15 min on ice. Afterwards, the cells were rinsed again with 300 µL of ice-cold glycine buffer and the wash medium was combined with the respective supernatant. This fraction represents the amount of cell surface-bound ligand. In the last step, the cells were lysed by addition of 300 µL of 1 M aqueous NaOH. After 15 min, the lysate of each well was transferred to the respective vial as well as 300 µL of 1 M NaOH used for rinsing the well. This fraction represents the amount of internalized radioligand. Quantification of the amount of free, cell surface-bound and internalized activity was performed in a γ‑counter. The corresponding internalization values were corrected for non-specific binding and normalized to the specific binding observed for the reference [^177^Lu]Lu-DOTA-PP F11N.

*Lipophilicity*. The log D_7.4_ values (logarithmic *n*-octanol/PBS (pH = 7.4) distribution coefficients) were determined, using the shake-flask method as previously described.^(4)^ The radiolabeled compound (ca. 1 MBq, 2 - 6 μL radioligand stock solution) was dissolved in 1 mL of a 1/1 mixture (*v/v*) of PBS (494 - 498 µL) and *n*-octanol (500 µL) in a 1.5 mL reaction vial (n = 6). After vigorous mixing of the suspension for 3 min at r.t., the vial was centrifuged at 9000 rpm (ca. 7700 × g) for 5 min at r.t. (Heraeus Pico 17 microcentrifuge; Thermo Fisher Scientific Inc., Waltham, MA, United States) and 200 µL aliquots of both layers were measured separately in a ɣ-counter.

*Binding to human serum albumin.* Binding to human serum albumin (HSA) was determined by incubation of the radioligand (ca. 0.5 MBq, 25 µL radioligand stock solution diluted in PBS) in a human serum albumin (fatty acid‑free, globulin-free, ≥ 99%, A3782, Merck KGaA, Darmstadt, Germany) solution (225 µL, 777.8 µM in PBS) to reach a final concentration of 700 µM for human serum albumin and a total volume of 250 µL. Probes (n = 6 for HSA binding and n = 6 for control experiments in PBS) were incubated at 37 °C for 30 min. After transferring the solution to a Centrifree^®^ ultrafiltration device (Merck KGaA, Darmstadt, Germany) and centrifugation at 3200 rpm (ca. 1200 × g) for 40 min at r.t., the amount of free (filtrate) and non-filtered, membrane‑bound activity was measured in a ɣ‑counter. The fraction bound to HSA was calculated as the ratio of HSA‑bound activity (non-filtered, membrane‑bound) to the total activity in the ultrafiltration device. All values were corrected for non‑specific binding (control experiments in PBS).

*Stability studies in human serum*. The ^99m^Tc‑ or ^177^Lu‑labeled CCK-2R ligands (ca. 5 MBq, 14 µL radioligand stock solution) were incubated in human serum (type AB, obtained from human male AB plasma, Cat‑No: HUM-3B; free sample from Capricorn Scientific GmbH, Ebsdorfergrund, Germany) at 37 °C for 4 h or 24 h (total volume: 200 µL). After incubation, ice-cold EtOH (125 µL) and MeCN (375 µL) were added and the suspension was centrifuged (5000 rpm, ca. 2300 × g) for 5 min at r.t. The supernatant was transferred into new vials and centrifuged again (5000 rpm, 5 min, r.t.). The supernatant was separated from the precipitate and used for investigating the stability of the radioligands *via* radio‑RP‑HPLC (for ^99m^Tc-labeled peptides: 20 - 80% B in 15 min, Method A, MultoHigh Bio 300-5 C4 column; for ^177^Lu-labeled peptides: 30 - 50% B in 20 min, Method A, MultoKrom 100-5 C18 column).

1. **APPENDIX**
   1. **Analytical data of non-radioactive compounds**

**
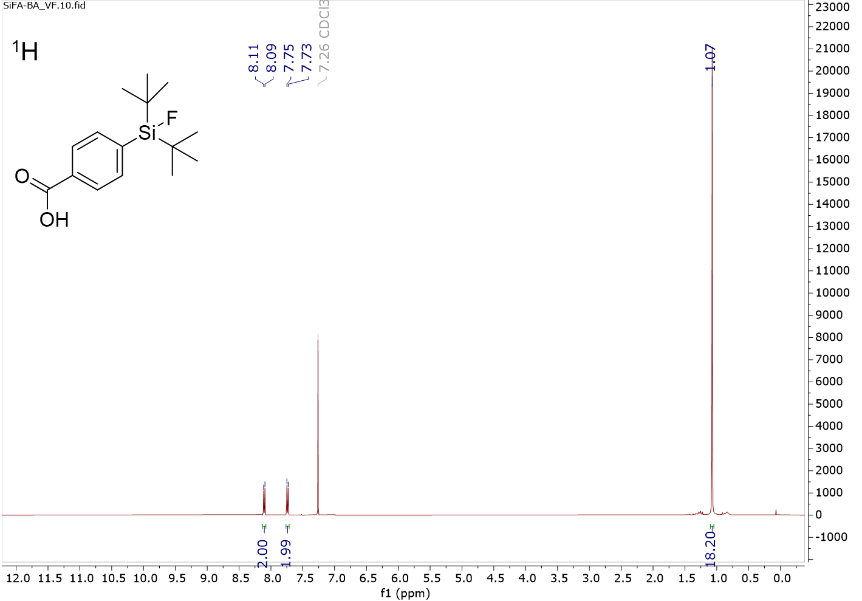
**

**
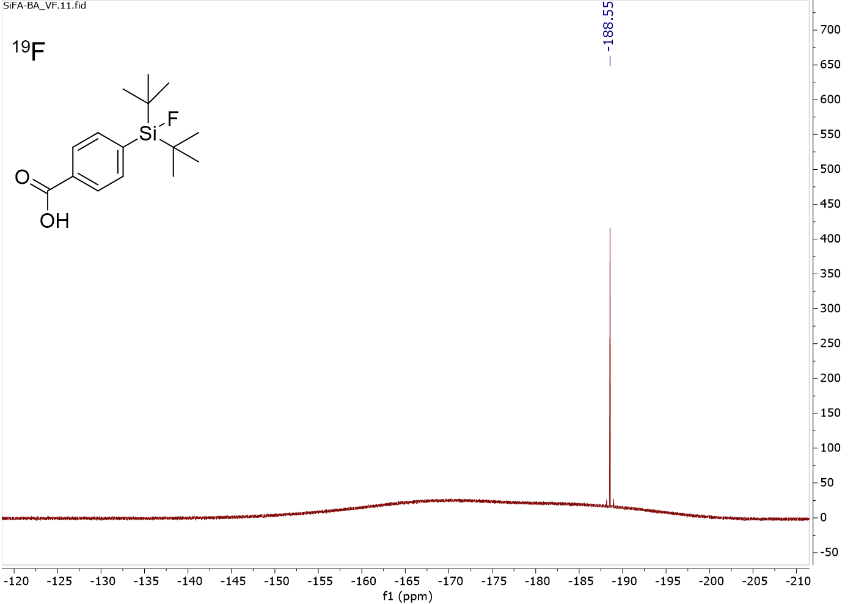
**

**
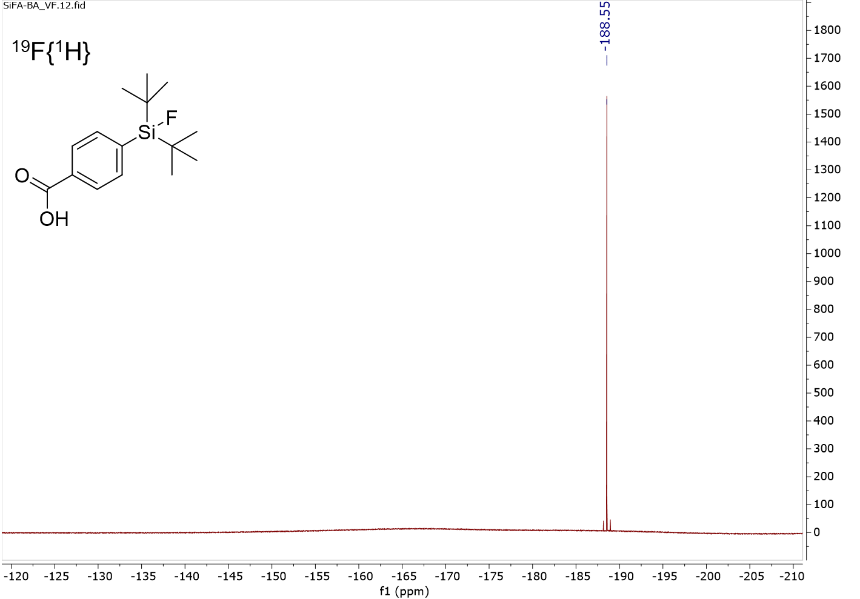
**

**Figure S1**. ^1^H-, ^19^F- and ^19^F{^1^H}-NMR spectra of SiFA-BA for comparison with SiOH-BA (**S1**, Figure S2). The characteristic ^19^F signal at -188.6 ppm indicates the presence of silicon-bound fluorine.

**
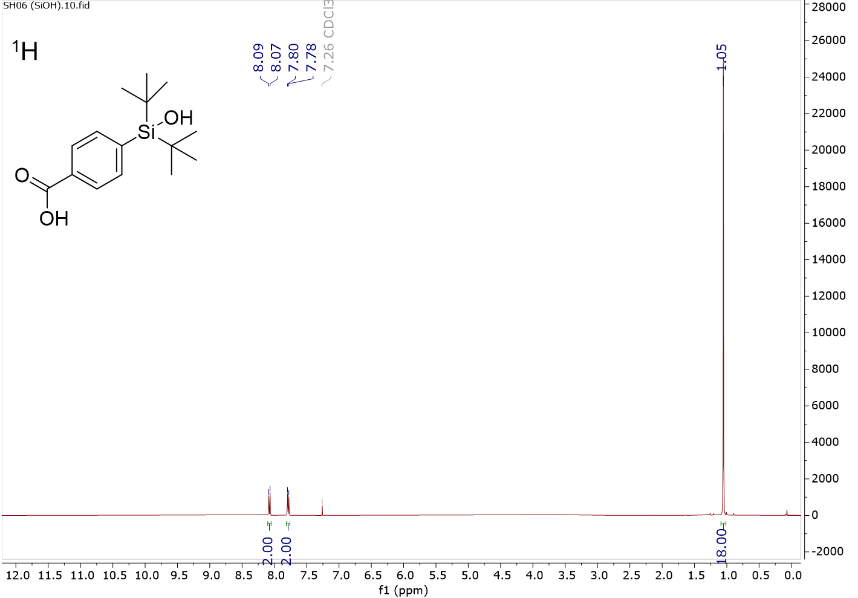
**

**
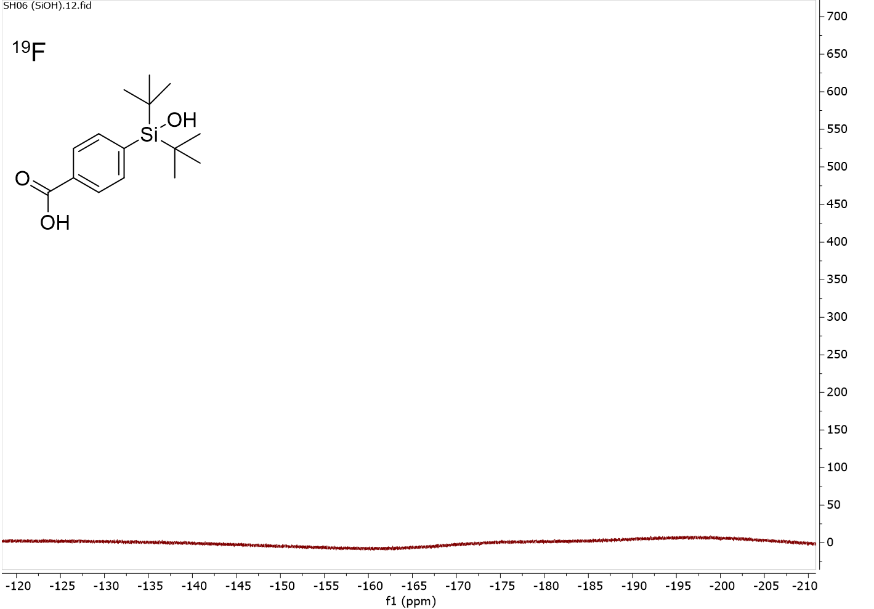
**

**
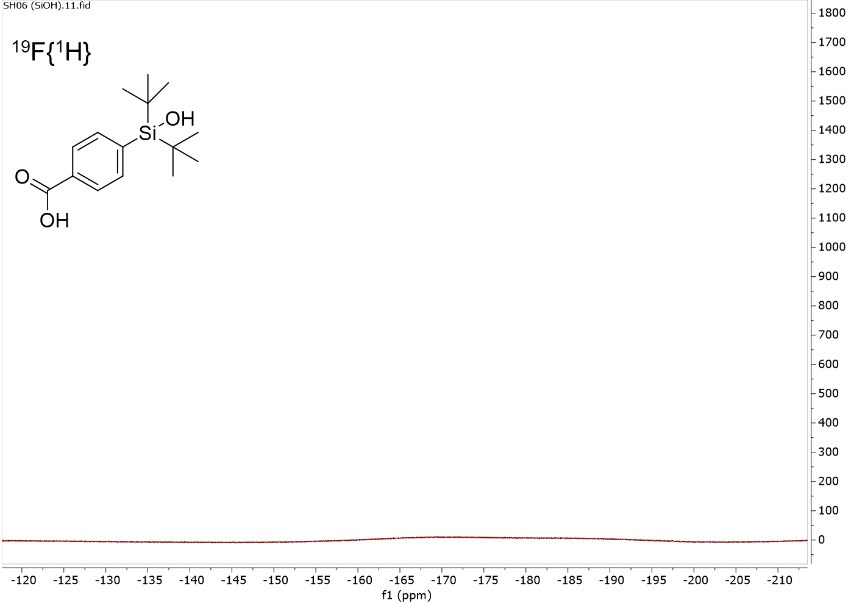
**

**Figure S2.** ^1^H-, ^19^F- and ^19^F{^1^H}-NMR spectra of SiOH-BA. Compared to SiFA-BA (Figure S1), the chemical shifts of ^1^H‑NMR signals almost remained unchanged. Besides, no fluorine signals could be detected in both ^19^F‑ and ^19^F{^1^H}-NMR spectra, indicating for quantitative hydrolysis of the Si-F bond.


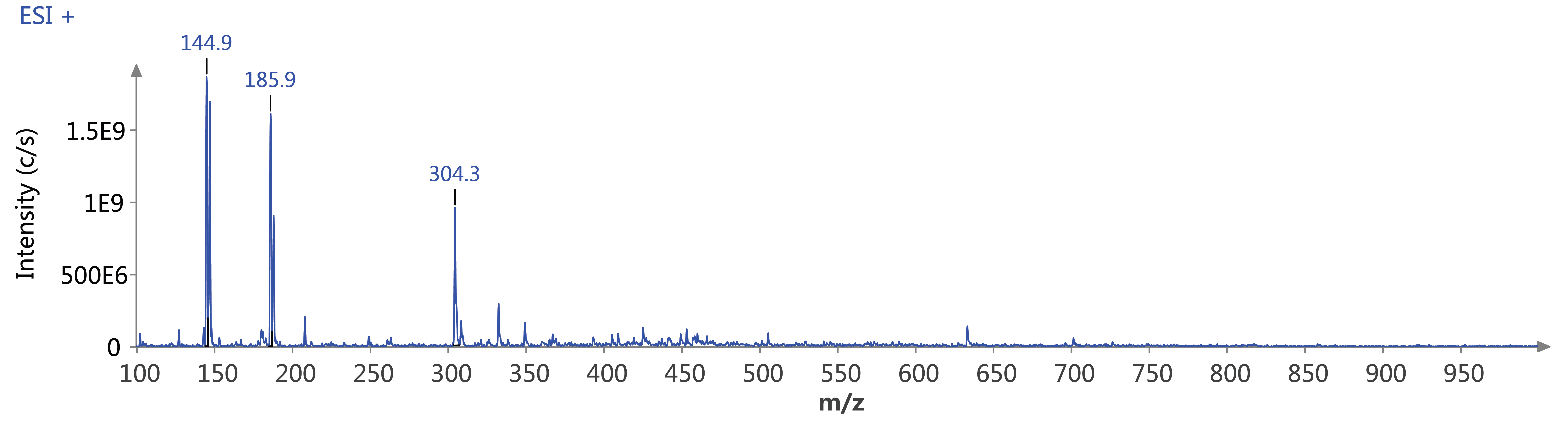


**Figure S3.** Mass spectrum of SiOH‑BA (**S1**) with m/z = 144.9 [M(Fragment 1 (SiOH‑BA))+Na^+^]^+^, 185.9 [M_2_(**S1**)+3H^+^]^3+^ and 304.3 [M(**S1**)+Na^+^]^+^.

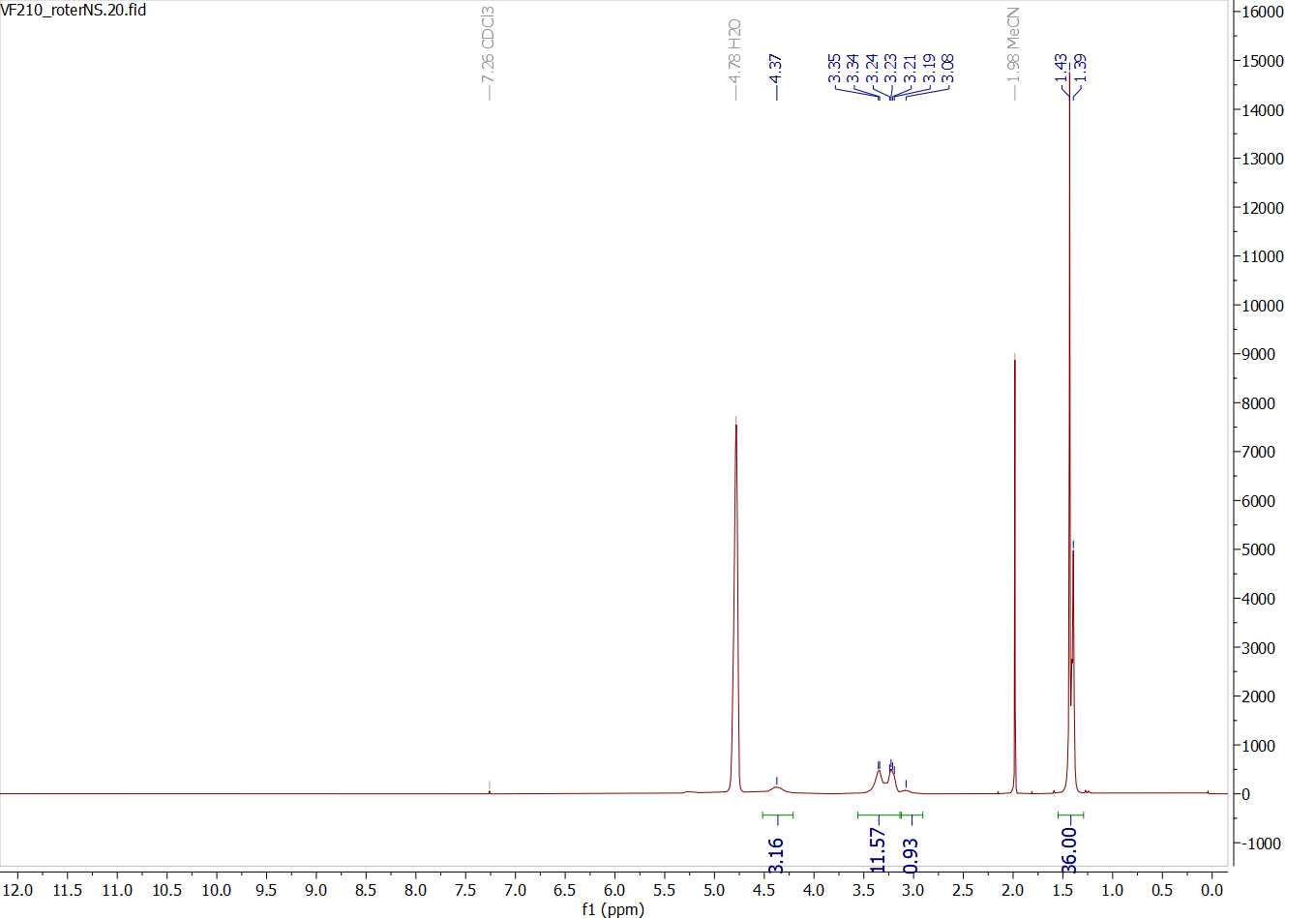


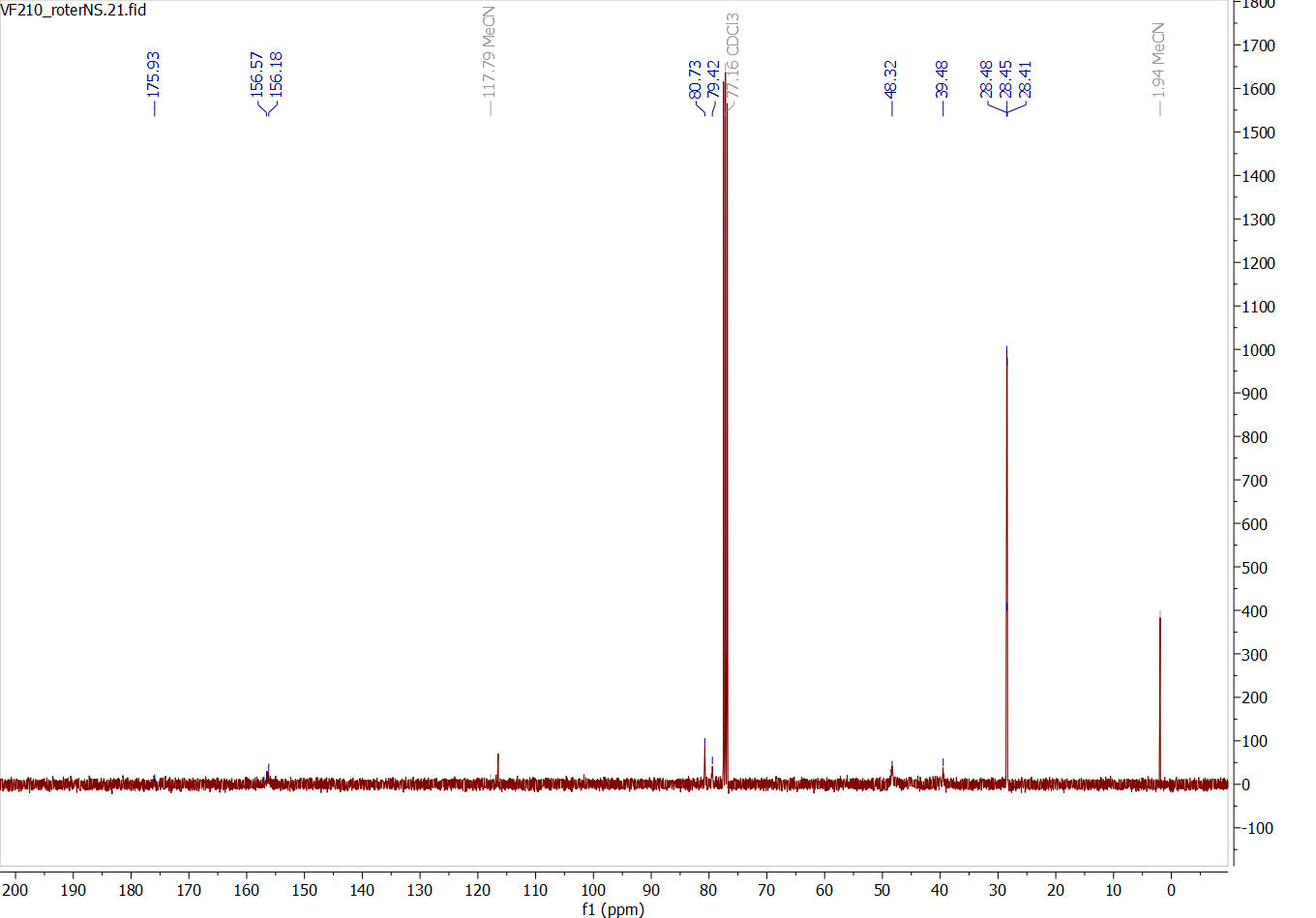


**Figure S4**. ^1^H- and ^13^C‑NMR spectrum of N_4_Boc_4_ (**S2**).


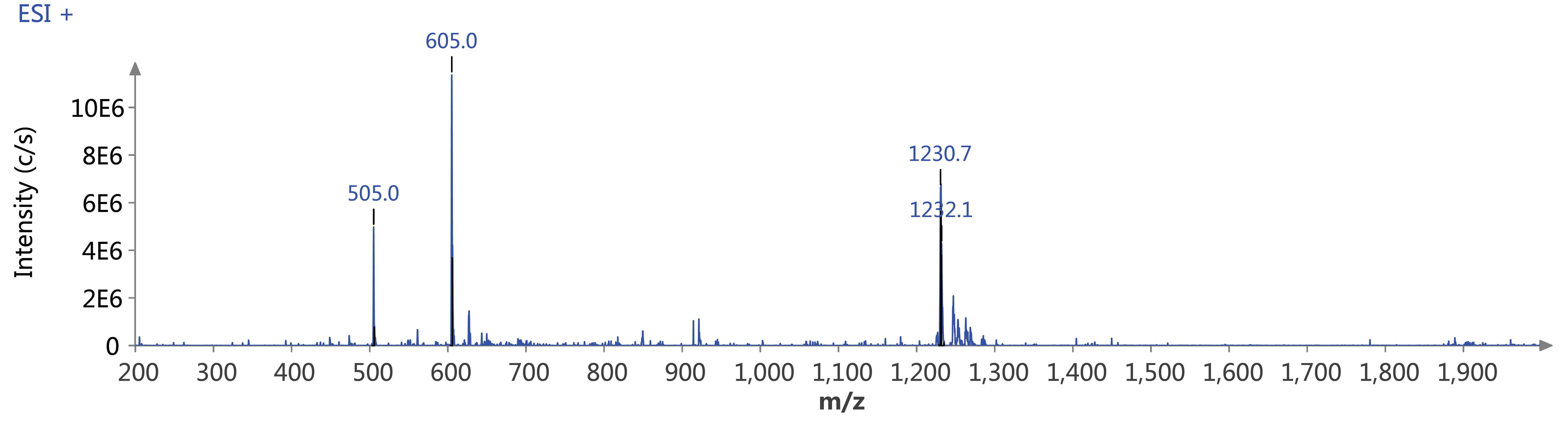


**Figure S5.** Mass spectrum of N_4_Boc_4_ with m/z = 505.0 [M(**S2**)-Boc+H^+^]^+^, 605.0 [M(**S2**)+H^+^]^+^ and 1230.7 [M_2_(**S2**)+Na^+^]^+^.

**DOTA‑PP‑F11N (2)**


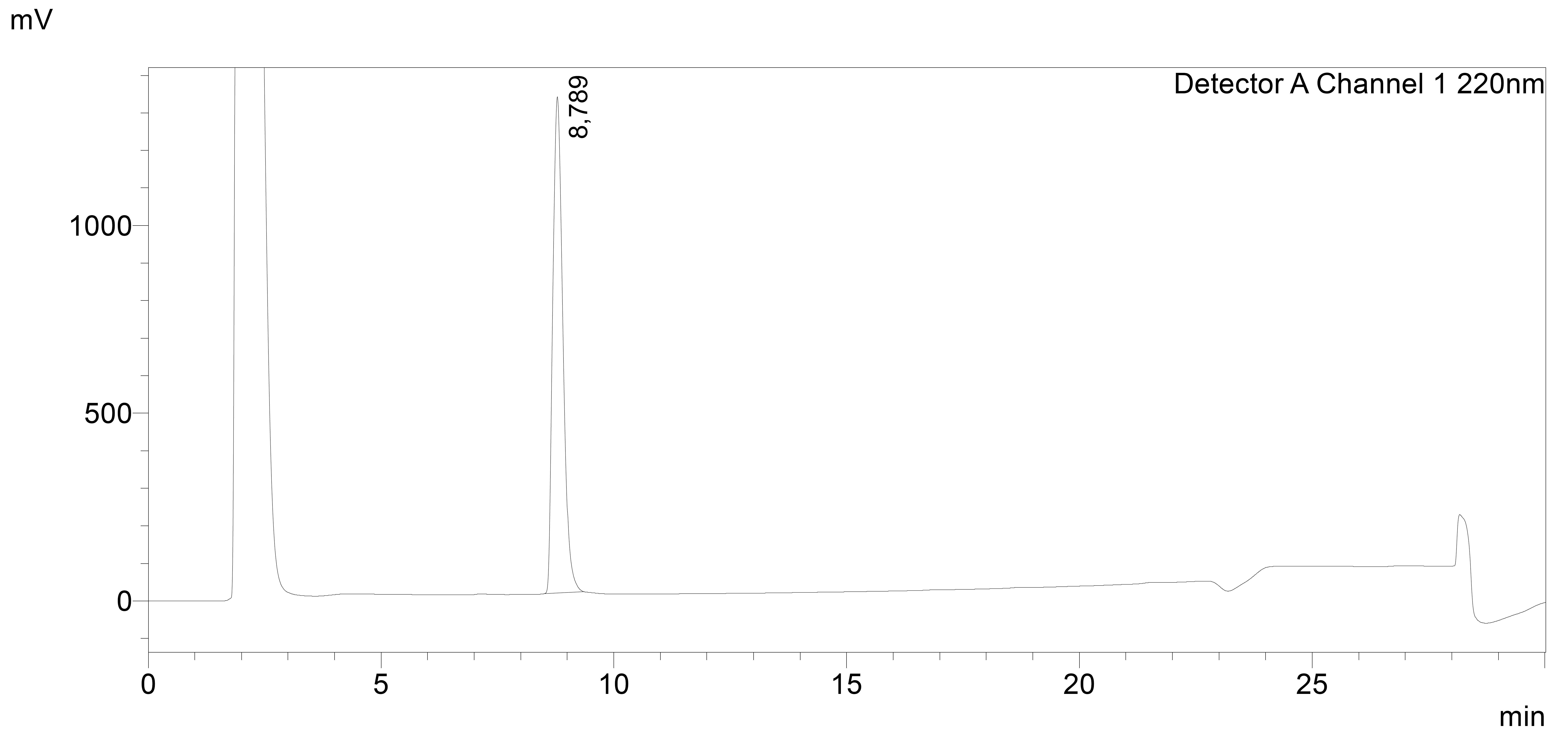


**Figure S6.** HPLC chromatogram of DOTA-PP-F11N (2.00 mM in DMSO), 20-80% B in 20 min (Method A); Chemical purity: >99%.


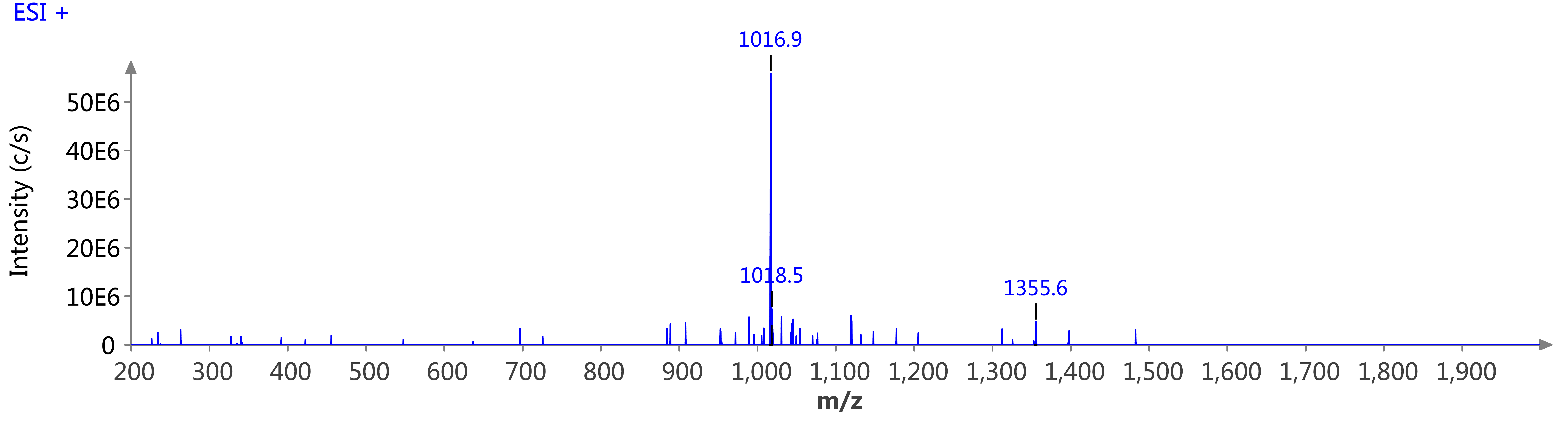


**Figure S7.** Mass spectrum of DOTA-PP-F11N (fraction collected at 8.8 min) with m/z = 1016.9 [M(**2**)+2H^+^]^2+^ and 1355.6 [M_2_(**2**)+3H^+^]^3+^.

**^nat^Lu‑DOTA‑PP‑F11N (^nat^Lu‑2)**


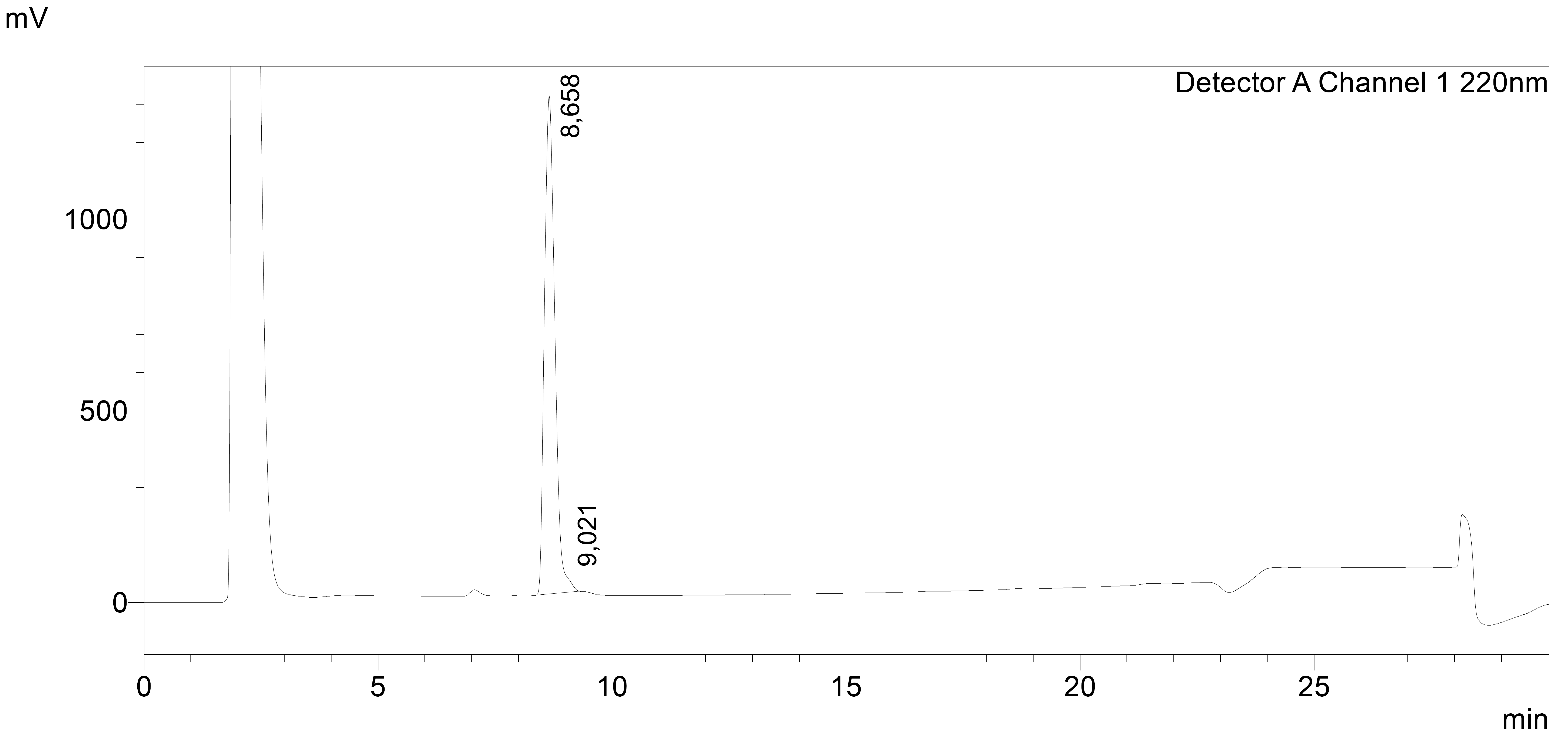


**Figure S8.** HPLC chromatogram of ^nat^Lu-DOTA-PP-F11N (1.00 mM in DMSO/TP-H_2_O = 1/1 and 2.50 eq. ^nat^LuCl_3_), 20-80% B in 20 min (Method A); Chemical purity: 98.4%.


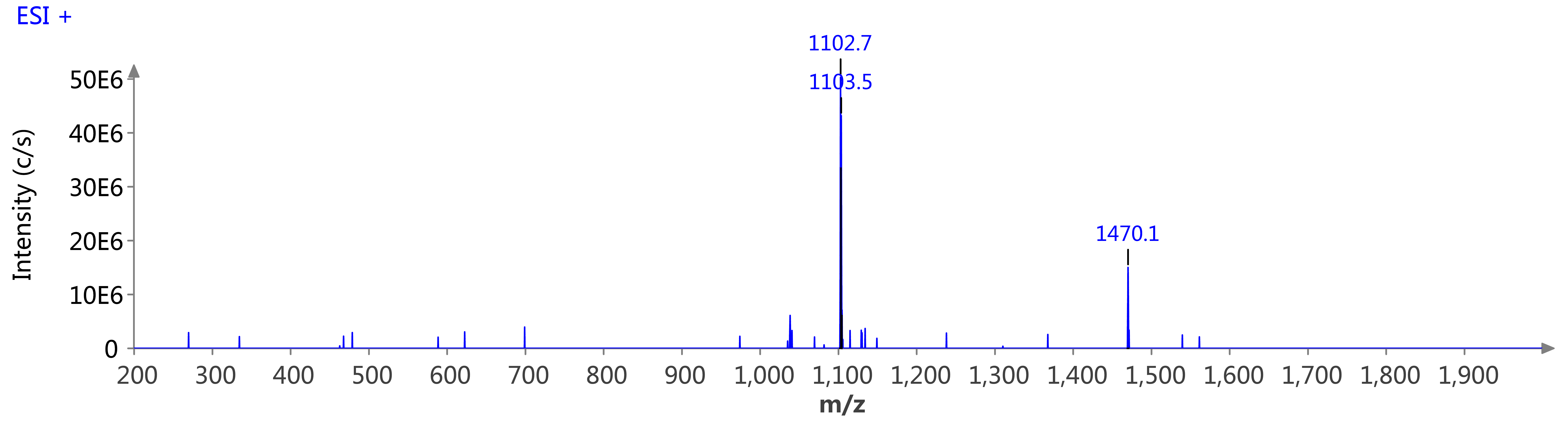


**Figure S9.** Mass spectrum of ^nat^Lu-DOTA-PP-F11N (fraction collected at 8.7 min) with m/z = 1102.7 [M(^nat^Lu‑**2**)+2H^+^]^2+^ and 1470.1 [M_2_(**2**)+3H^+^]^3+^.

**DOTA‑CCK‑66 (4)**


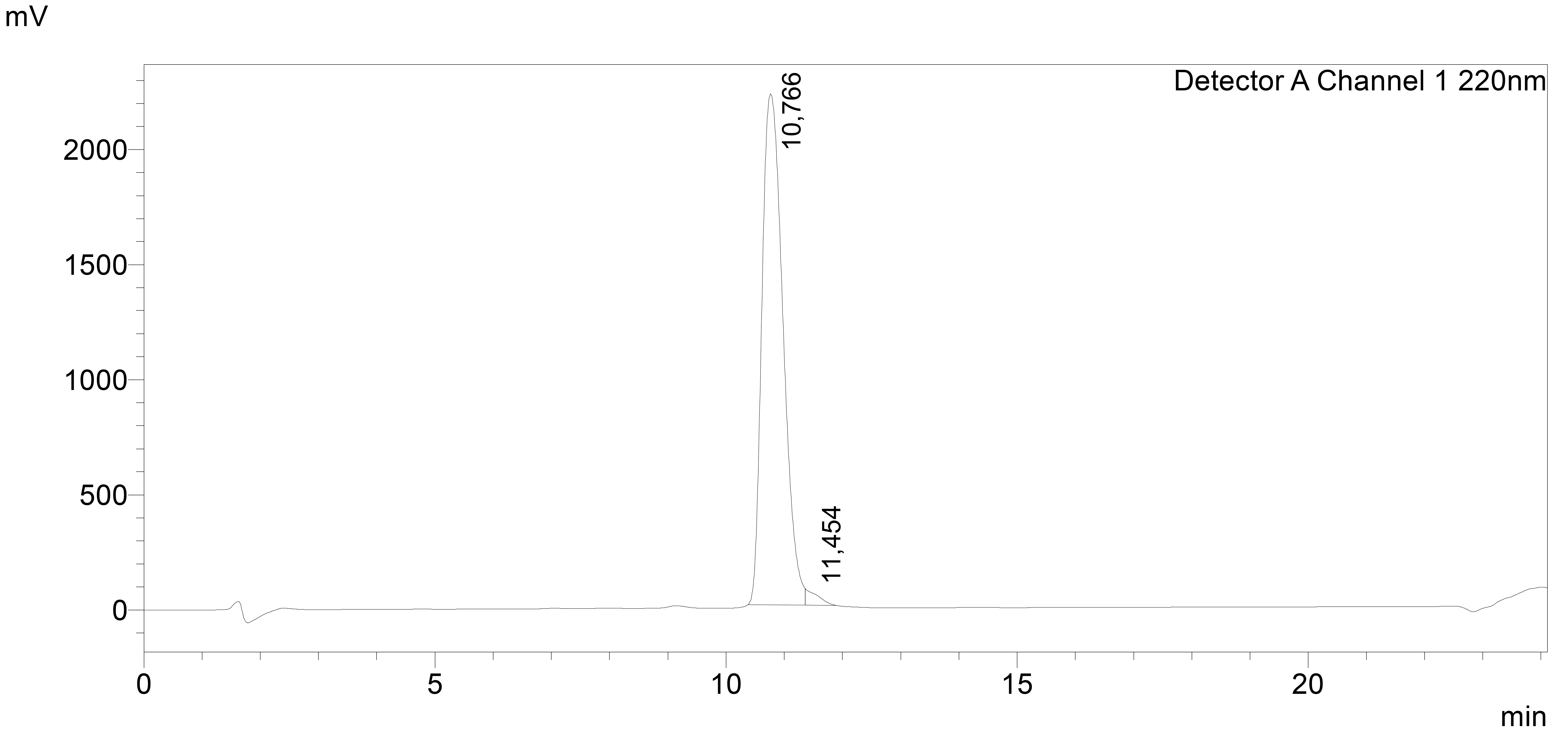


**Figure S10.** HPLC chromatogram of DOTA-CCK-66, 30-50% B in 20 min (Method A); Chemical purity: 98.2%.


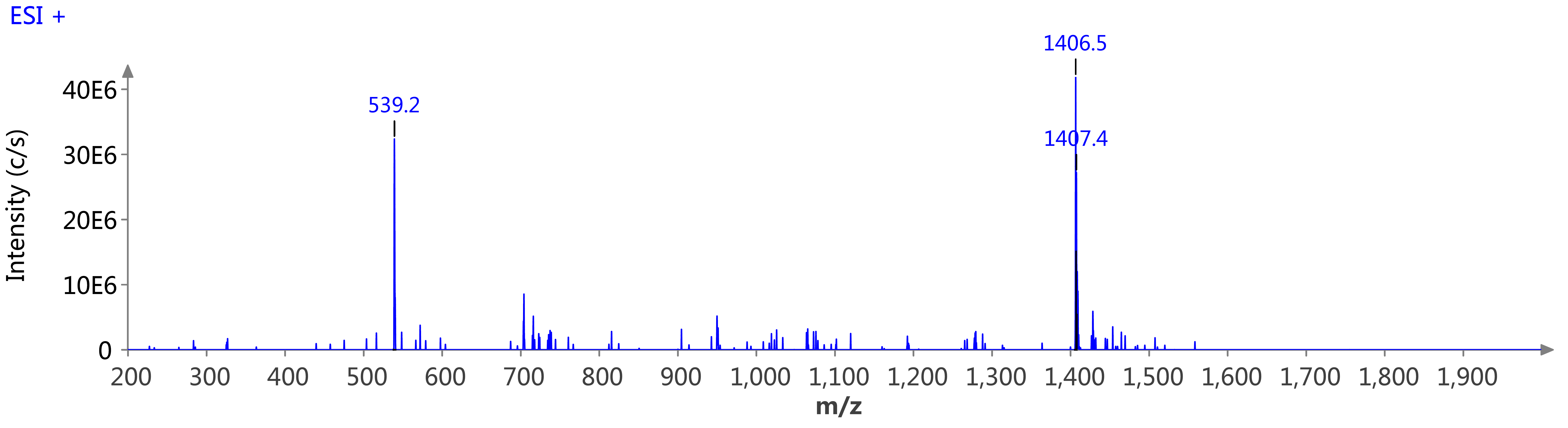


**Figure S11.** Mass spectrum of DOTA-CCK-66 (fraction collected at 10.8 min) with m/z = 539.2 [M(Fragment 1 (DOTA‑CCK‑66))+2H^+^]^2+^ and 1406.5 [M(**4**)+H^+^]^+^.

**^nat^Lu‑DOTA‑CCK‑66 (^nat^Lu‑4)**


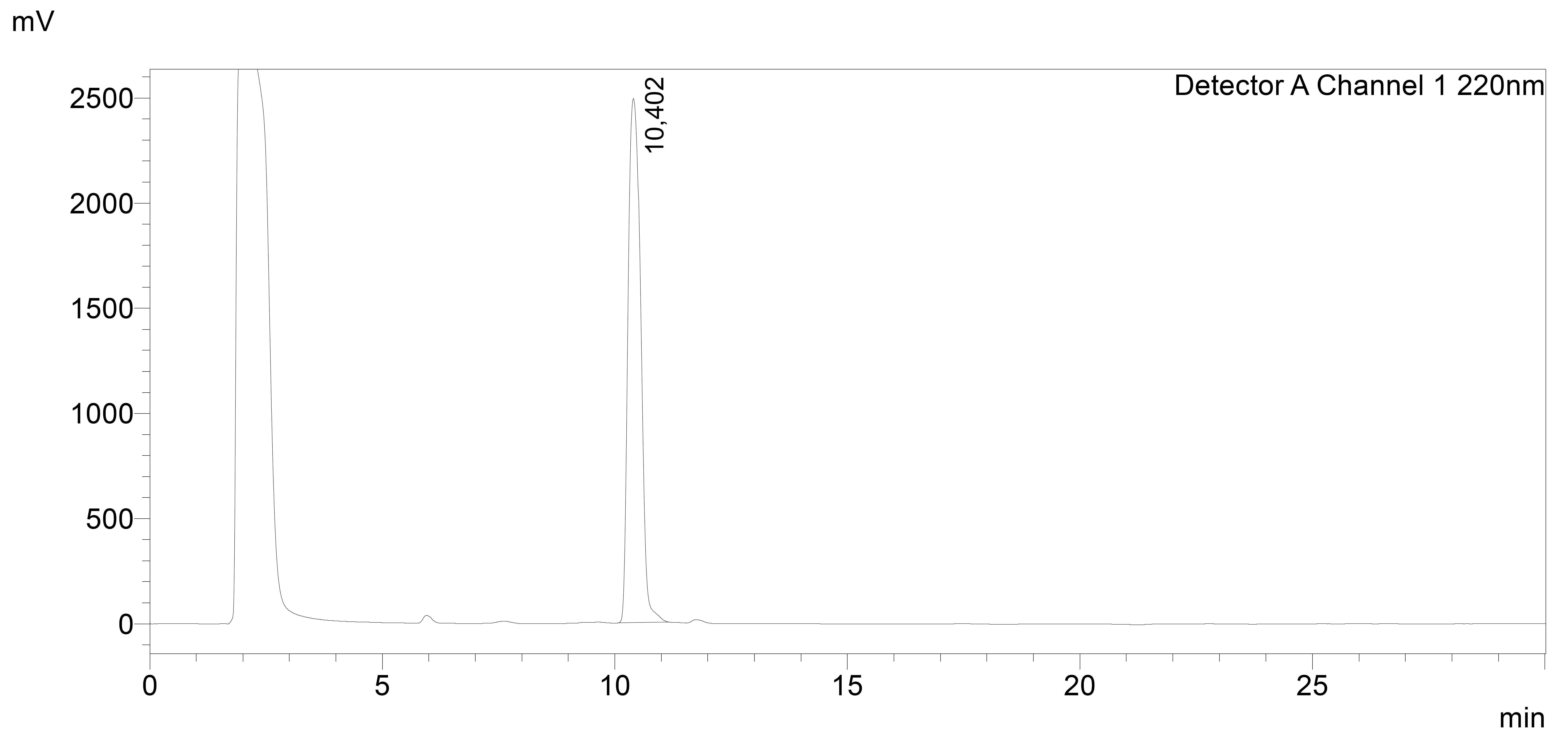


**Figure S12.** HPLC chromatogram (background-corrected) of ^nat^Lu-DOTA-CCK-66 (0.50 mM in DMSO/TP‑H_2_O = 1/1 and 2.50 eq. ^nat^LuCl_3_), 20-80% B in 20 min (Method A); Chemical purity: >99%.


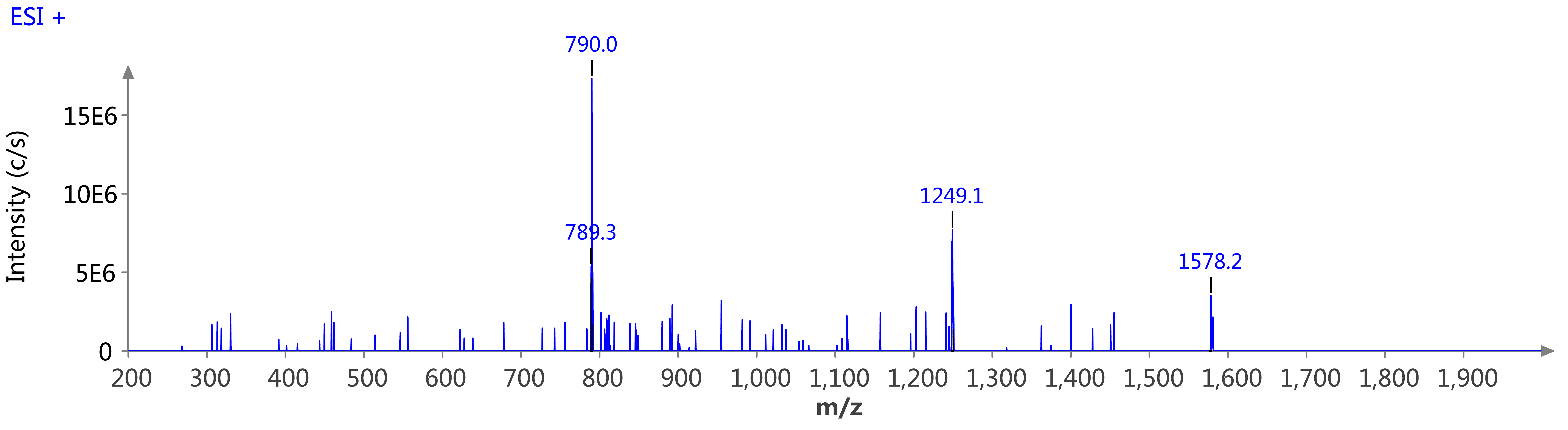


**Figure S13.** Mass spectrum of ^nat^Lu-DOTA-CCK-66 (fraction collected at 10.4 min) with m/z = 790.0 [M(^nat^Lu‑**4**)+2H^+^]^2+^, 1249.1 [M(Fragment 1 (^nat^Lu-DOTA-CCK-66))+H^+^]^+^ and 1578.2 [M(^nat^Lu‑**4**)+H^+^]^+^.

**N_4_-CCK-66 (11)**


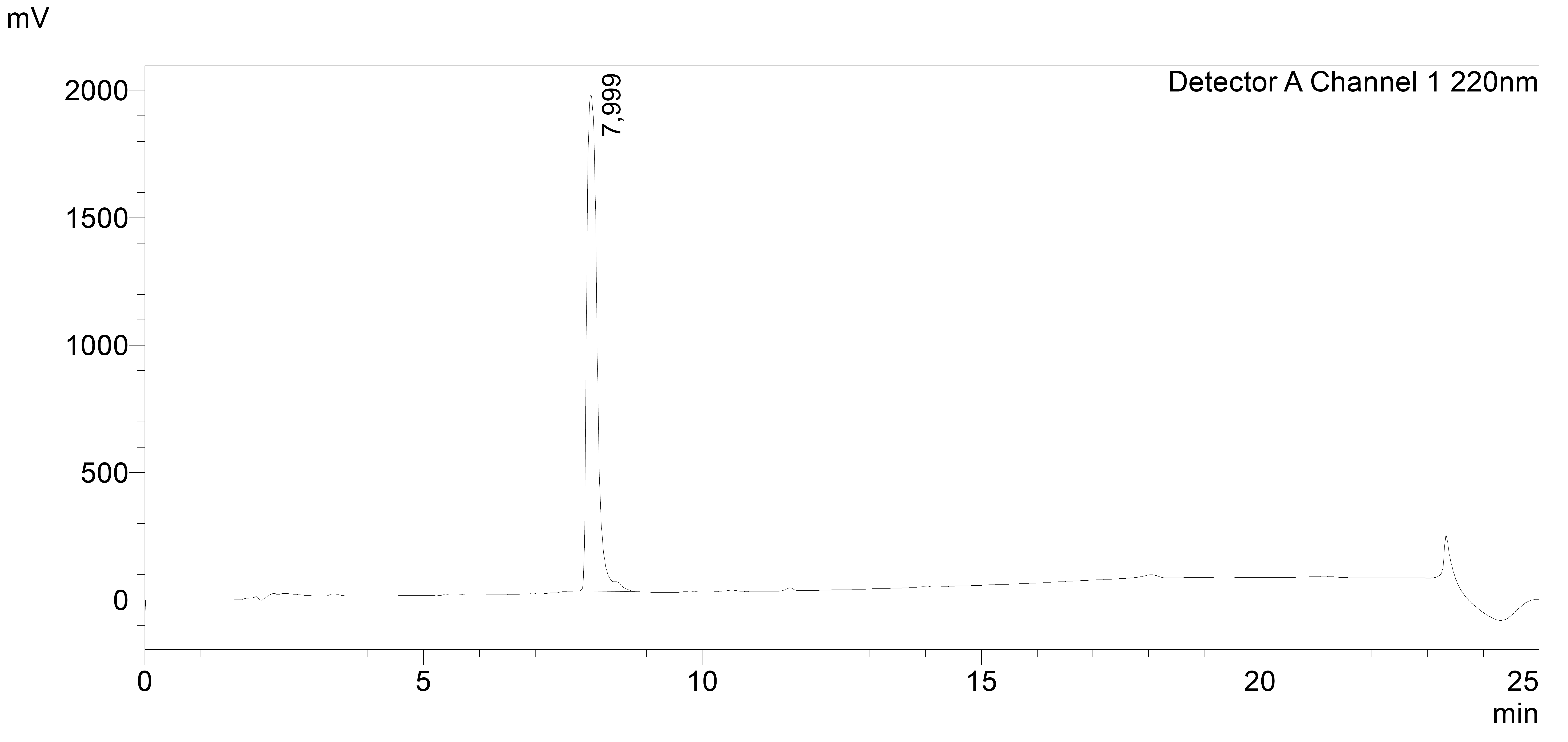


**Figure S14.** HPLC chromatogram of N_4_-CCK-66, 10-90% B in 15 min (Method A); Chemical purity >99%.


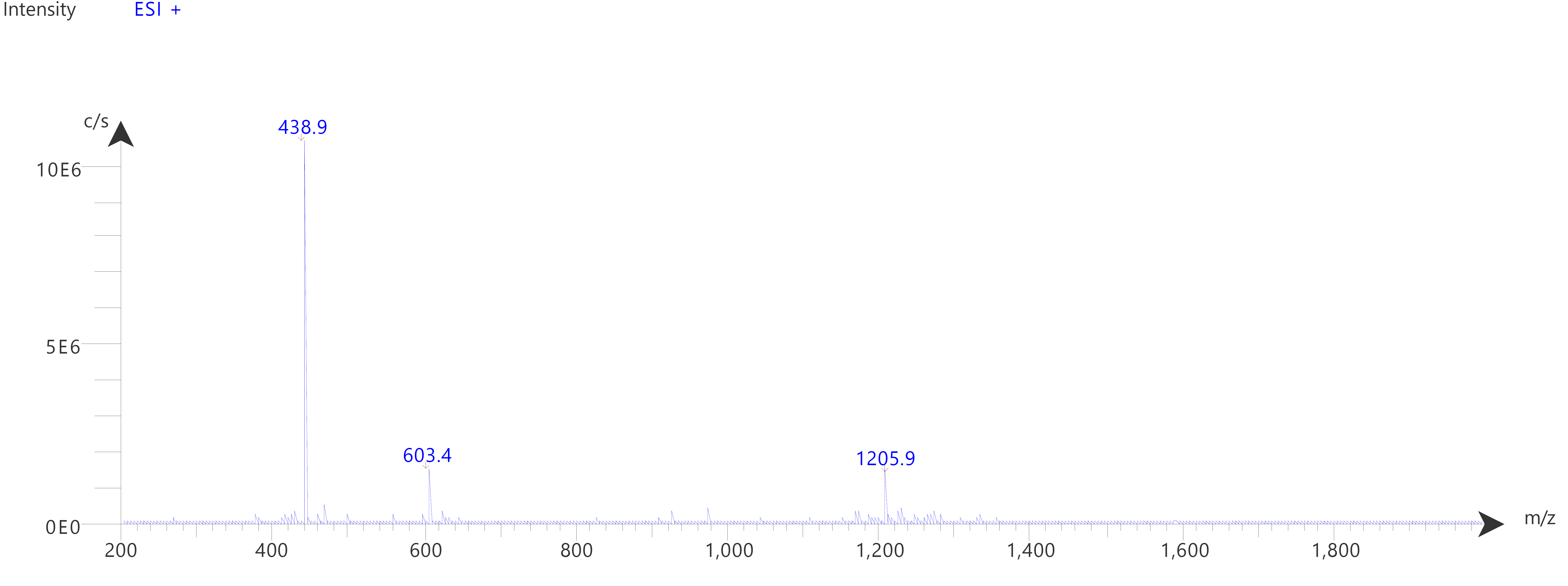


**Figure S15**. Mass spectrum of N_4_-CCK-66 (fraction collected at 8.0 min) with m/z = 438.9 [M(Fragment 1 (N_4_‑CCK‑66))+2H^+^]^2+^, 603.4 [M(**11**)+2H^+^]^2+^ and 1205.9 [M(**11**)+H^+^]^+^.

**N_4_-CCK-100 (12)**


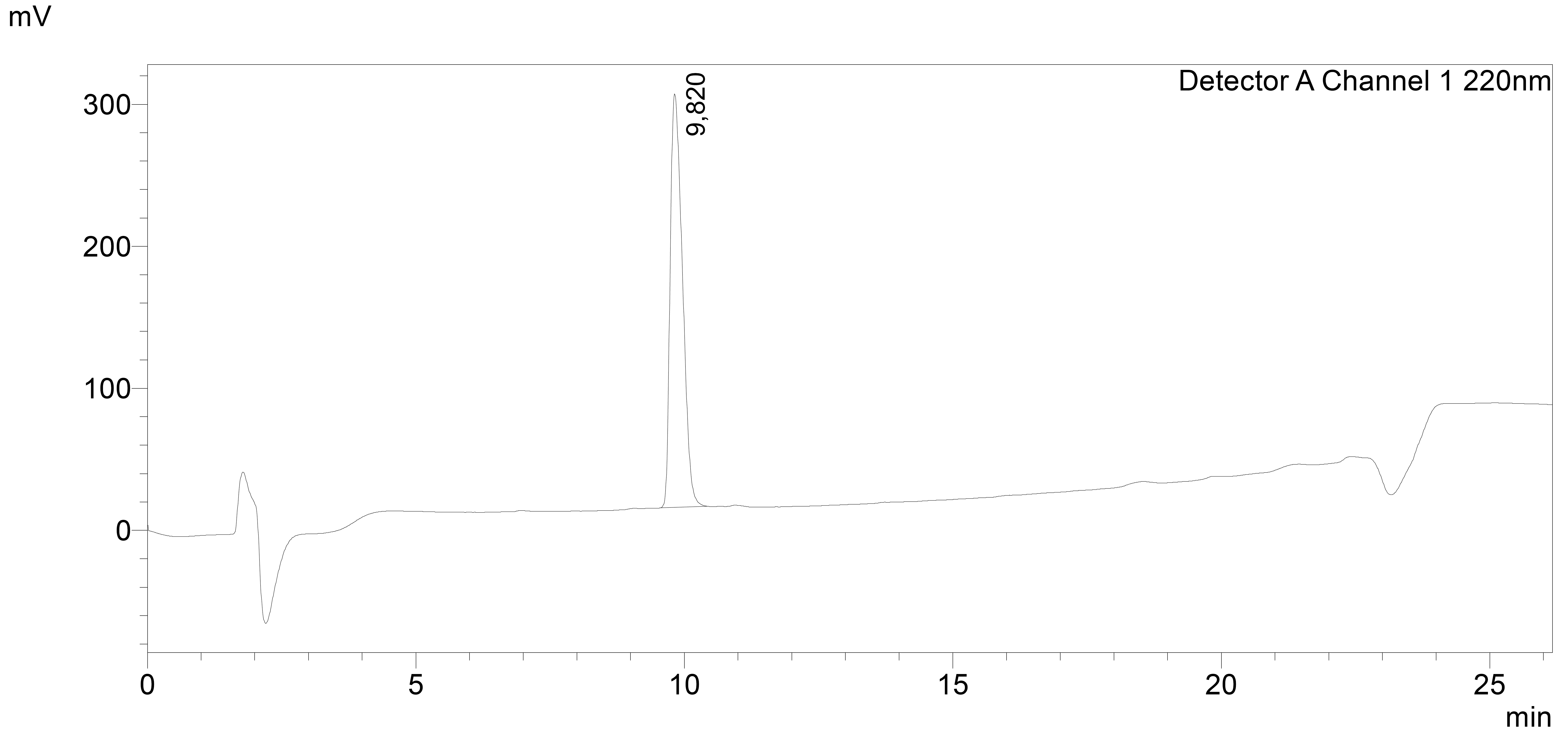


**Figure S16.** HPLC chromatogram of N_4_-CCK-100, 20-80% B in 20 min (Method A); Chemical purity >99%.


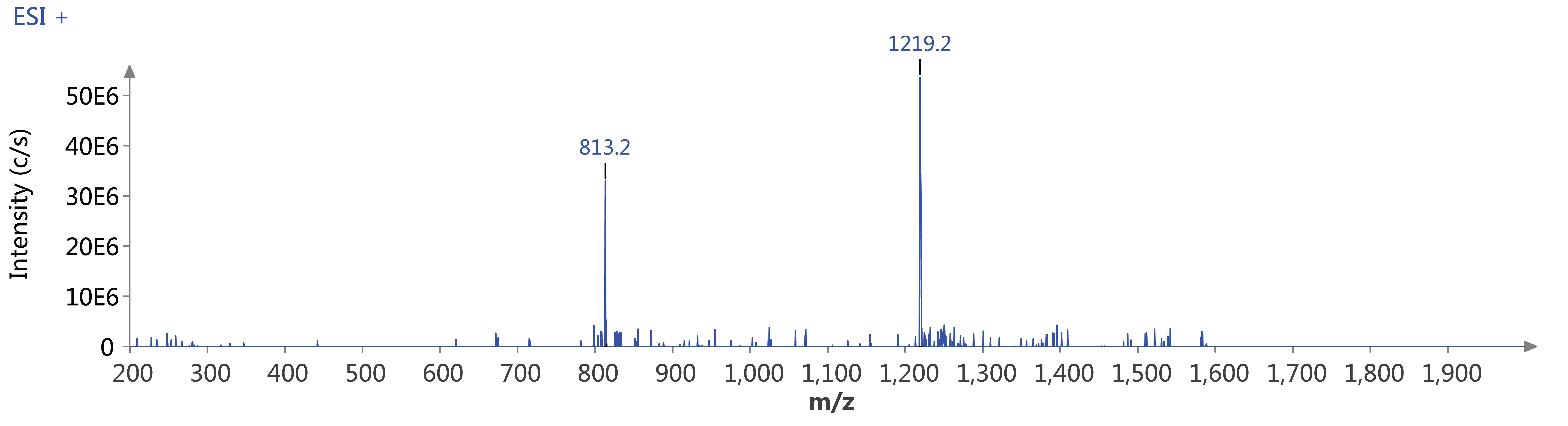


**Figure S17.** Mass spectrum of N_4_-CCK-100 (fraction collected at 9.8 min) with m/z = 813.2 [M(**12**)+3H^+^]^3+^ and 1219.2 [M(**12**)+2H^+^]^2+^.

**N_4_-CCK-101 (13)**


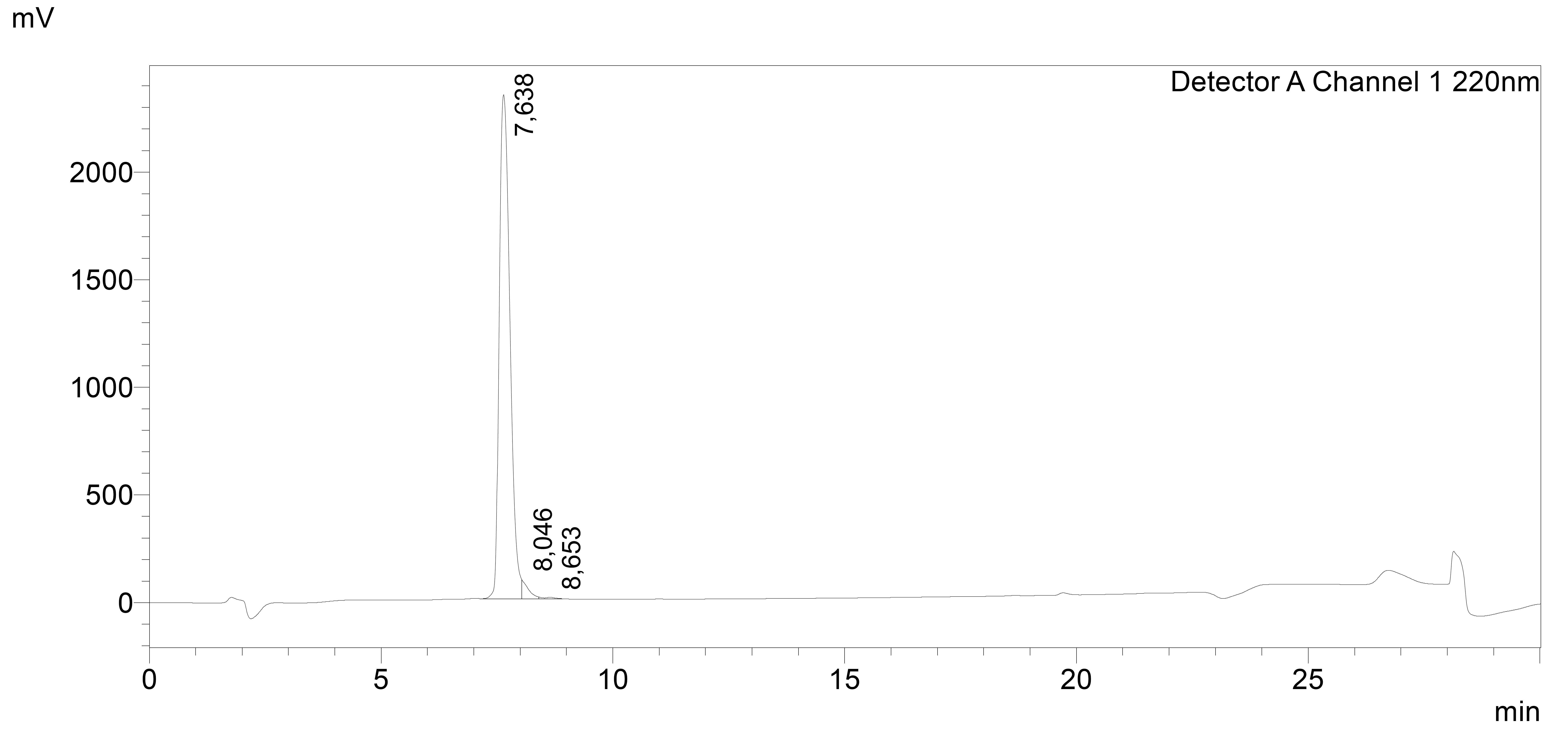


**Figure S18.** HPLC chromatogram of N_4_-CCK-101, 20-80% B in 20 min (Method A); Chemical purity: 97.5%.


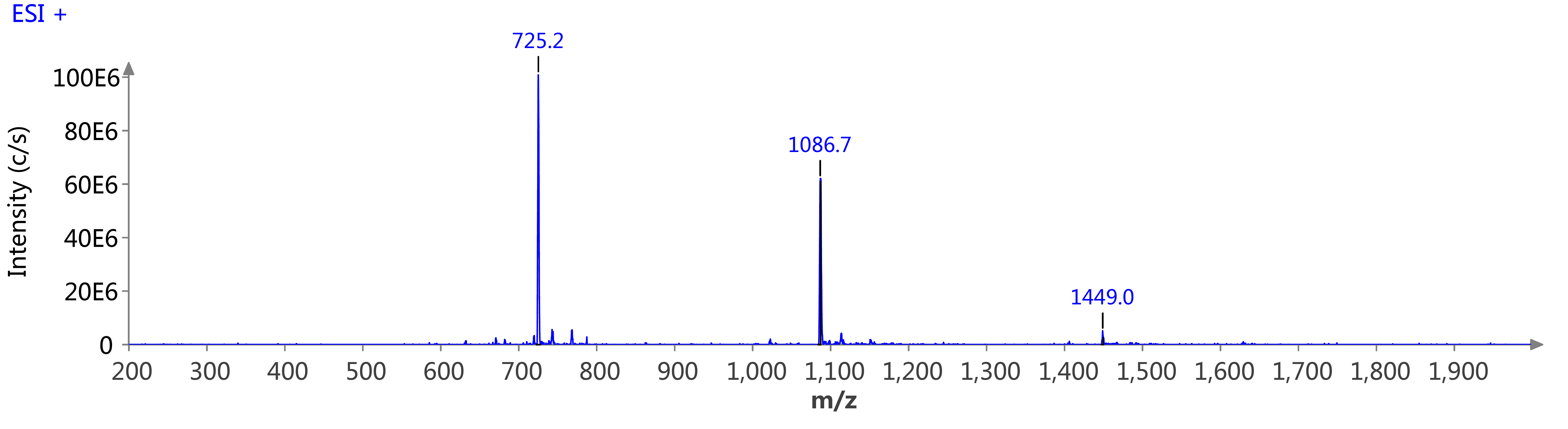


**Figure S19.** Mass spectrum of N_4_-CCK-101 (fraction collected at 7.6 min) with m/z = 725.2 [M(**13**)+3H^+^]^3+^, 1086.7 [M(**13**)+2H^+^]^2+^ and 1449.0 [M_2_(**13**)+3H^+^]^3+^.

**N_4_-CCK-102 (14)**


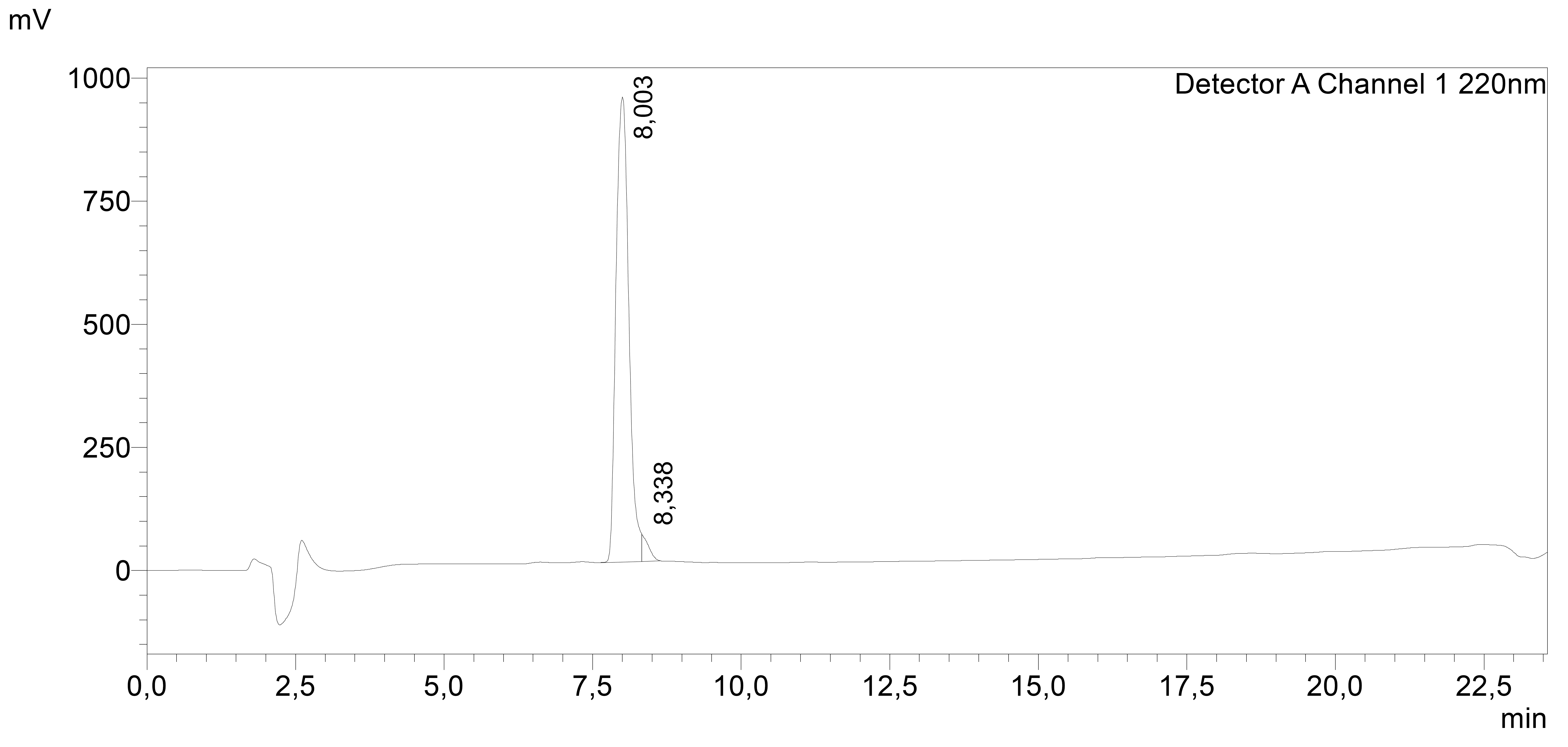


**Figure S20.** HPLC chromatogram of N_4_-CCK-102, 20-80% B in 20 min (Method A); Chemical purity: 97.1%.


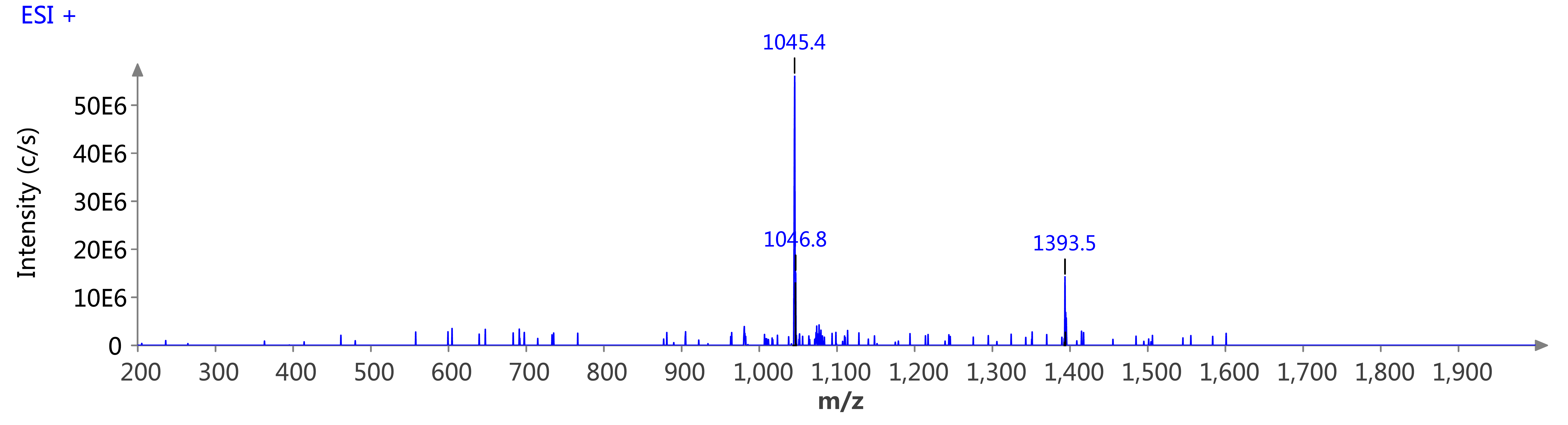


**Figure S21.** Mass spectrum of N_4_-CCK-102 (fraction collected at 8.0 min) with m/z = 1045.4 [M(**14**)+2H^+^]^2+^ and m/z = 1393.5 [M_2_(**14**)+3H^+^]^3+^.

**N_4_-CCK-103 (15)**


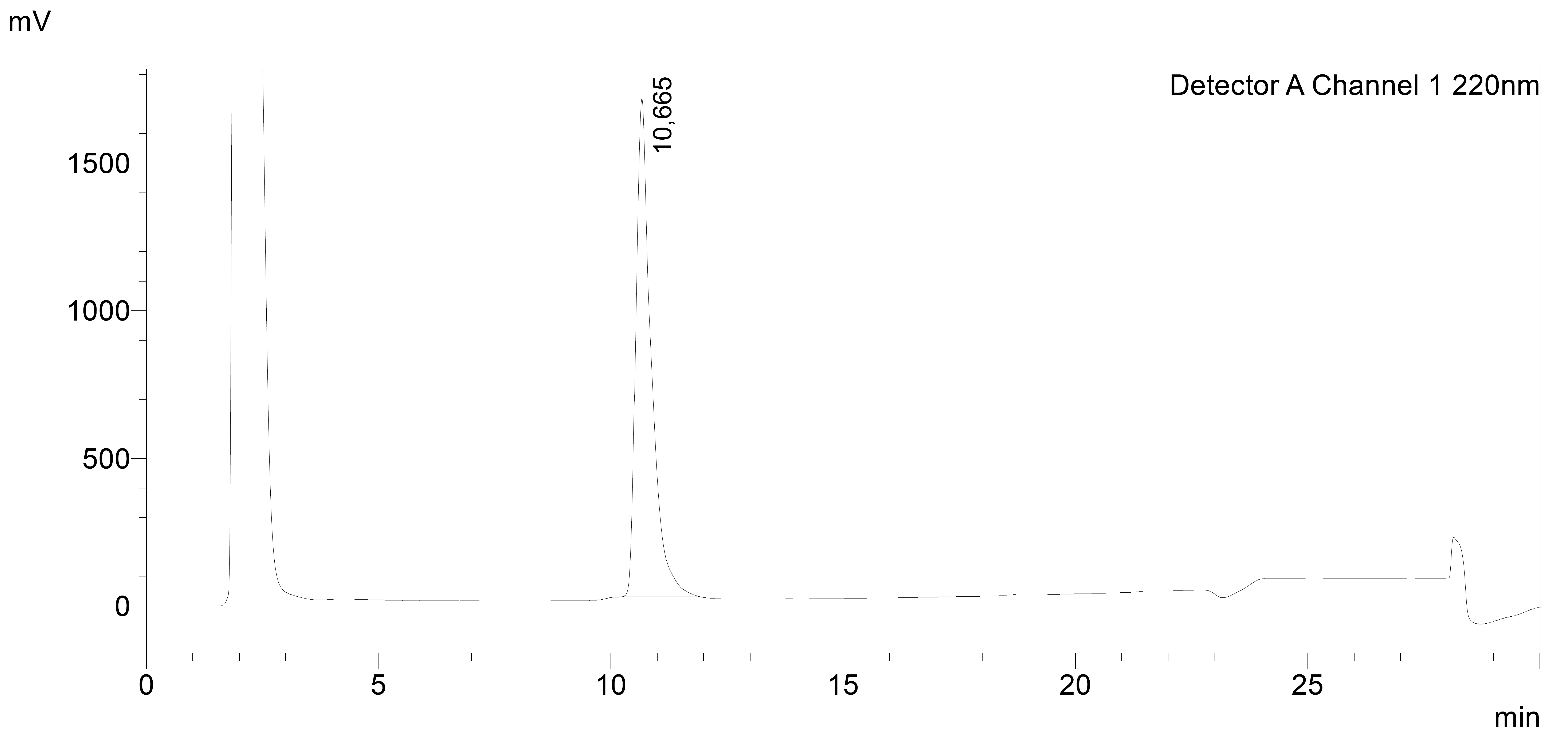


**Figure S22.** HPLC chromatogram of N_4_-CCK-103 (1.00 mM in DMSO), 20-80% B in 20 min (Method A); Chemical purity: >99%.


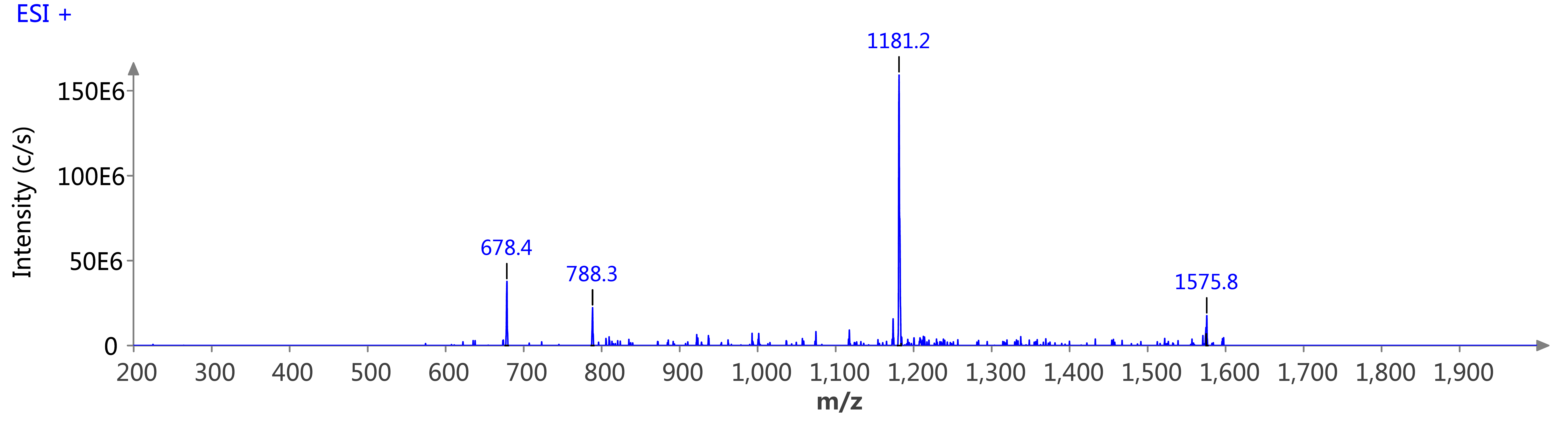


**Figure S23.** Mass spectrum of N_4_-CCK-103 (fraction collected at 10.7 min) with m/z = 678.4 [M(Fragment 1(N_4_‑CCK‑103)+3H^+^]^3+^, 788.3 [M(**15**)+3H^+^]^3+^, 1181.2 [M(**15**)+2H^+^]^2+^ and 1575.8 [M_2_(**15**)+3H^+^]^3+^.

**N_4_-CCK-104 (16)**


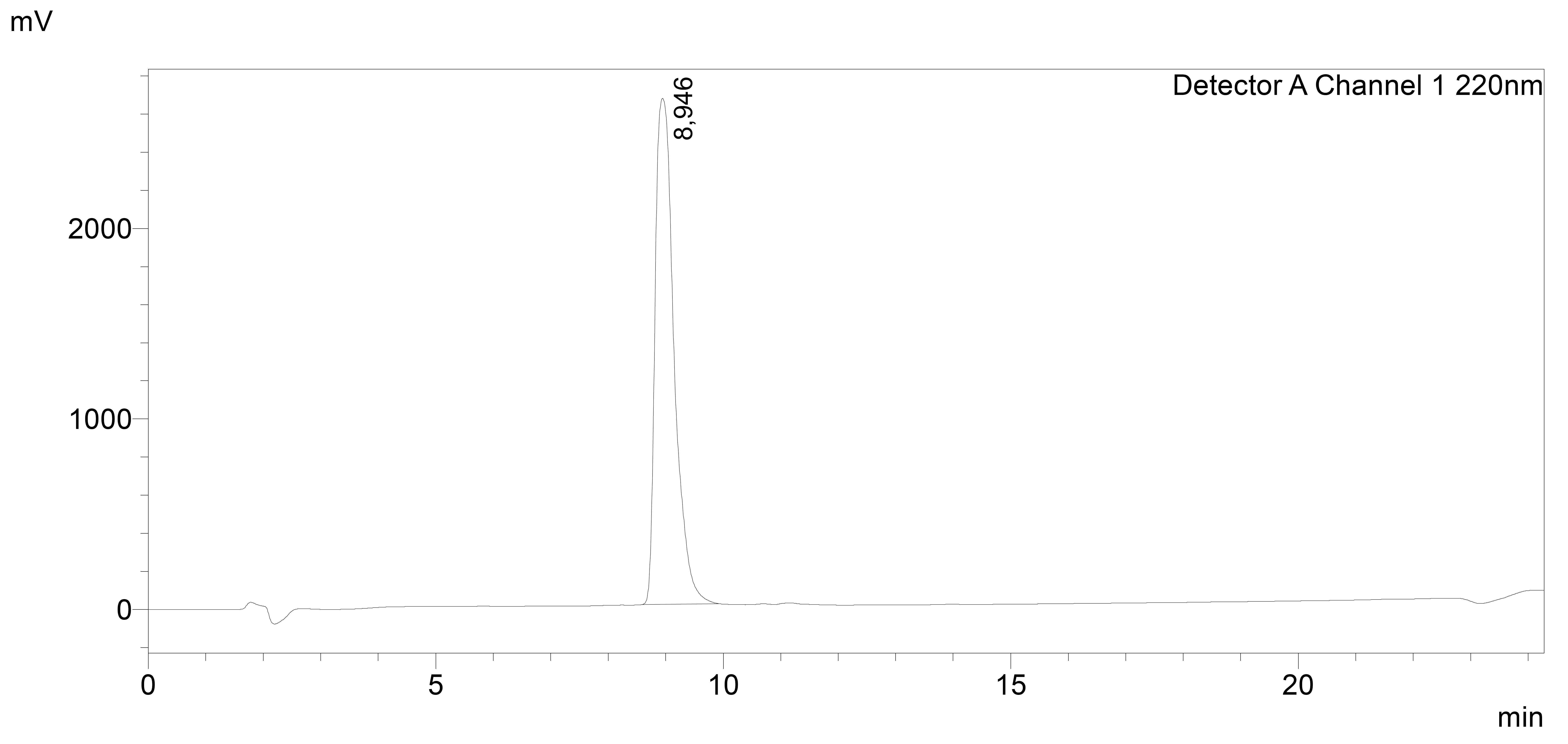


**Figure S24.** HPLC chromatogram of N_4_-CCK-104, 20-80% B in 20 min (Method A); Chemical purity: >99%.


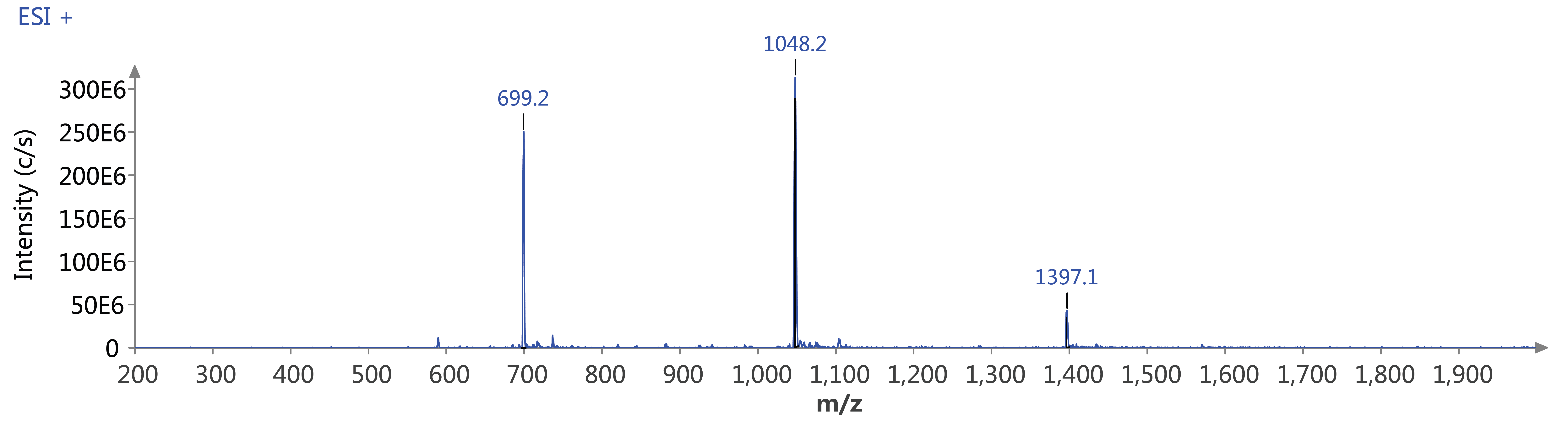


**Figure S25.** Mass spectrum of N_4_-CCK-104 (fraction collected at 8.9 min) with m/z = 699.2 [M(**16**)+3H^+^]^3+^, 1048.2 [M(**16**)+2H^+^]^2+^ and 1397.1 [M_2_(**16**)+3H^+^]^3+^.

**N_4_-CCK-105 (17)**


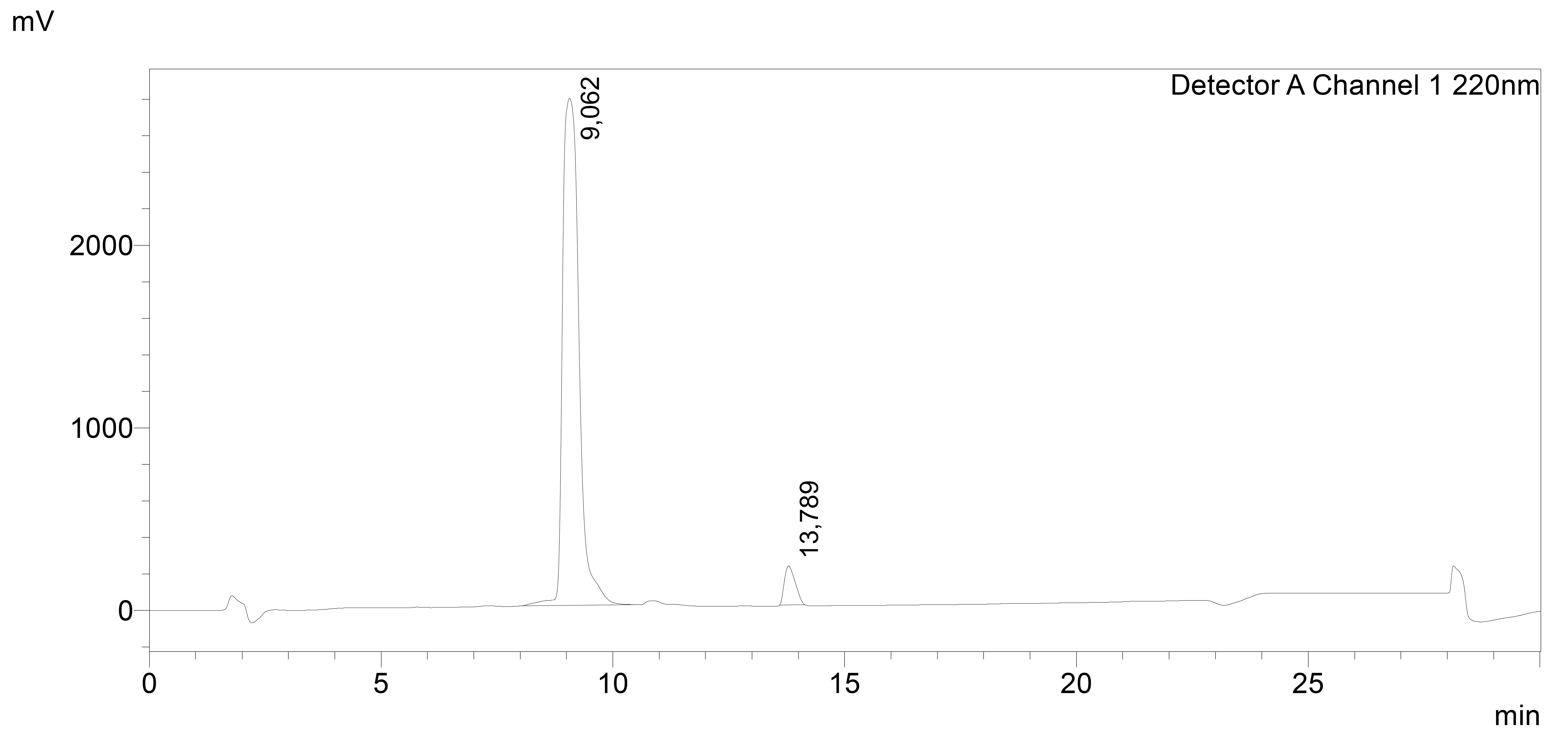


**Figure S26.** HPLC chromatogram of N_4_-CCK-105, 20-80% B in 20 min (Method A); Chemical purity: 95.1%.


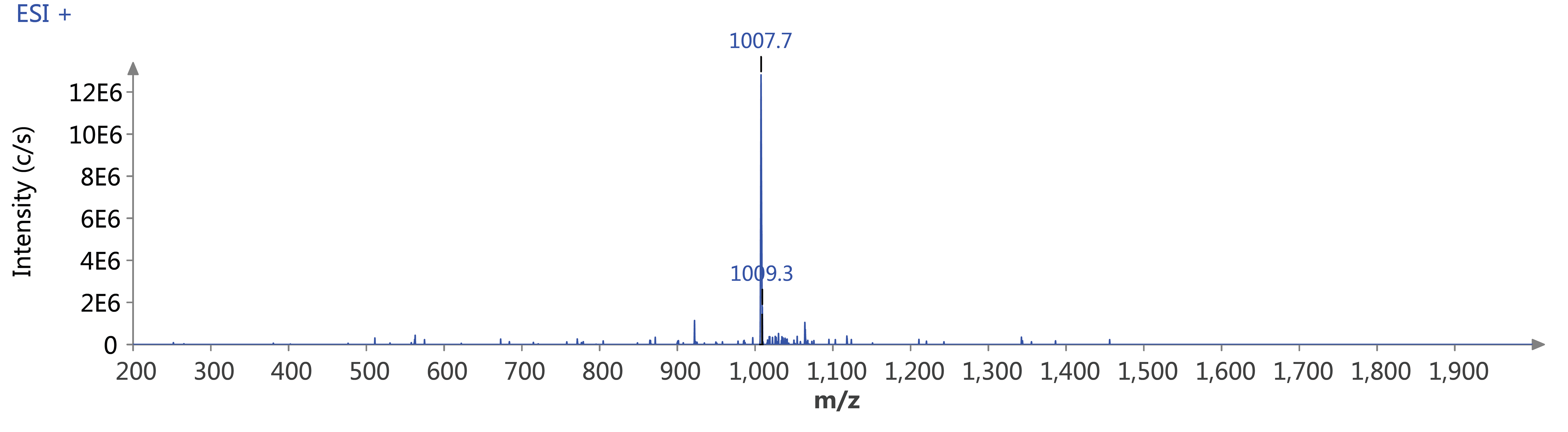


**Figure S27.** Mass spectrum of N_4_-CCK-105 (fraction collected at 9.1 min) with m/z = 1007.7 [M(**17**)+2H^+^]^2+^.

- 1. **Analytical data of radiolabeled compounds**

**[^177^Lu]Lu-DOTA-PP‑F11N ([^177^Lu]Lu‑2)**


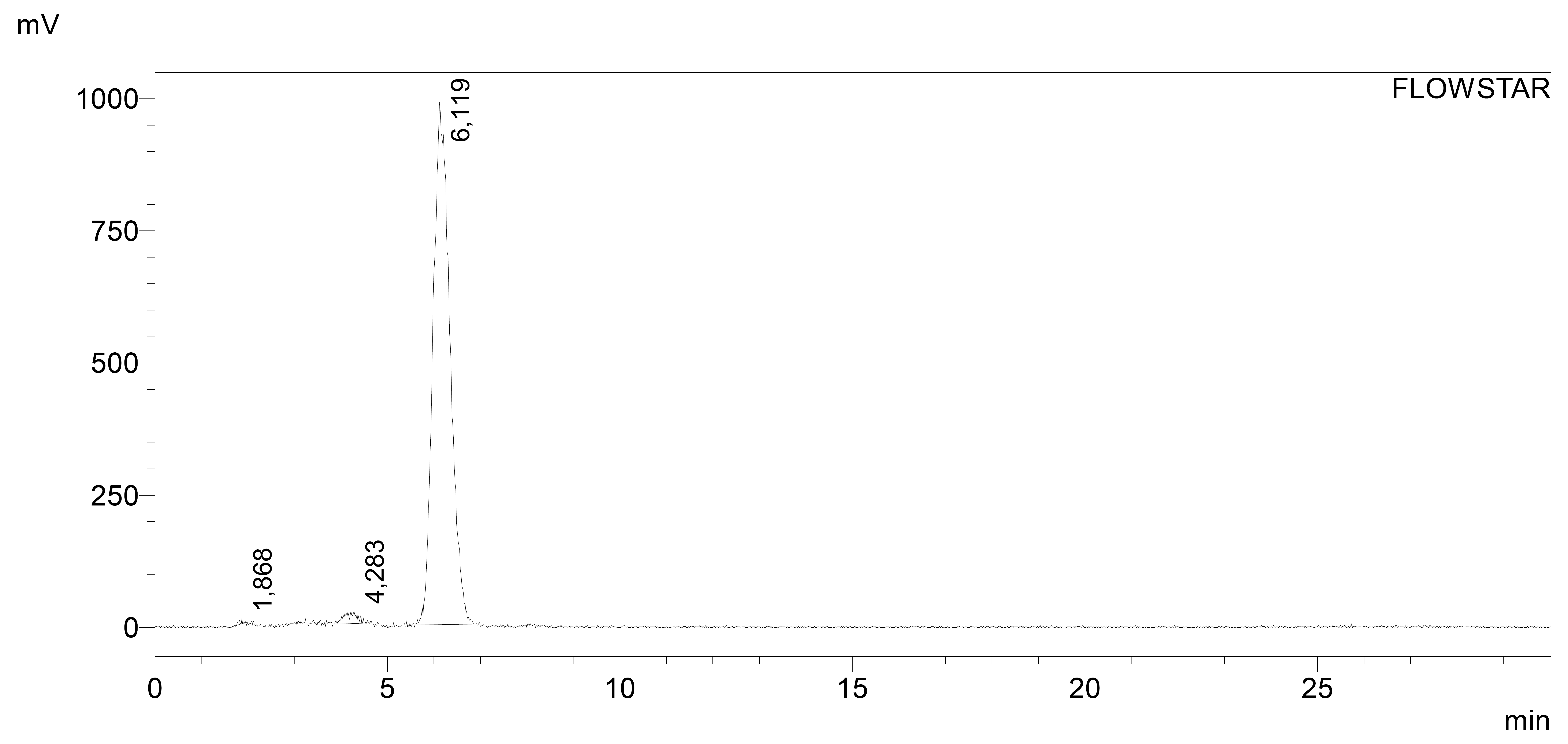


**Figure S28.** Radio‑HPLC chromatogram of [^177^Lu]Lu-DOTA-PP‑F11N, 30-50% B in 20 min (Method A); RCP: 98.2%.


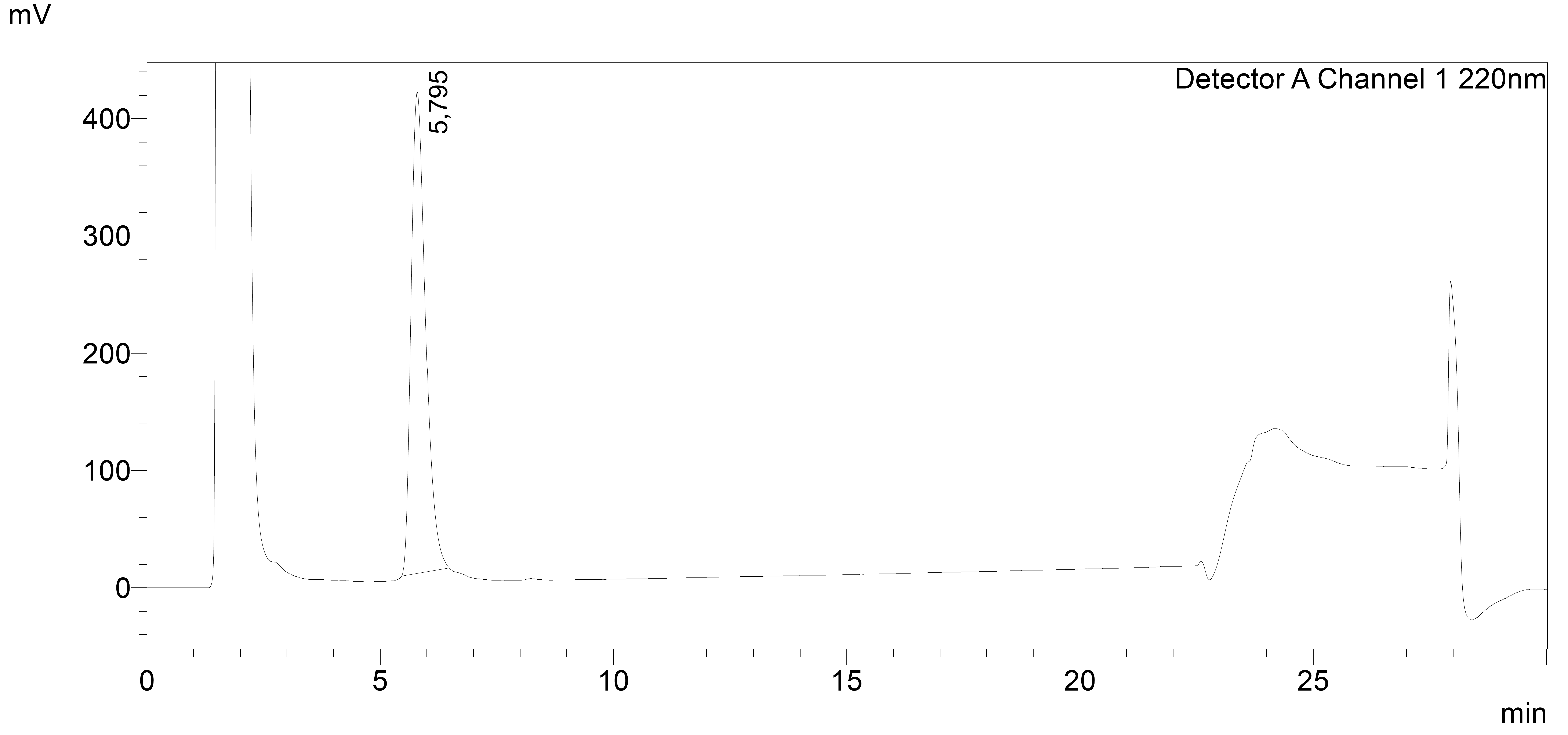

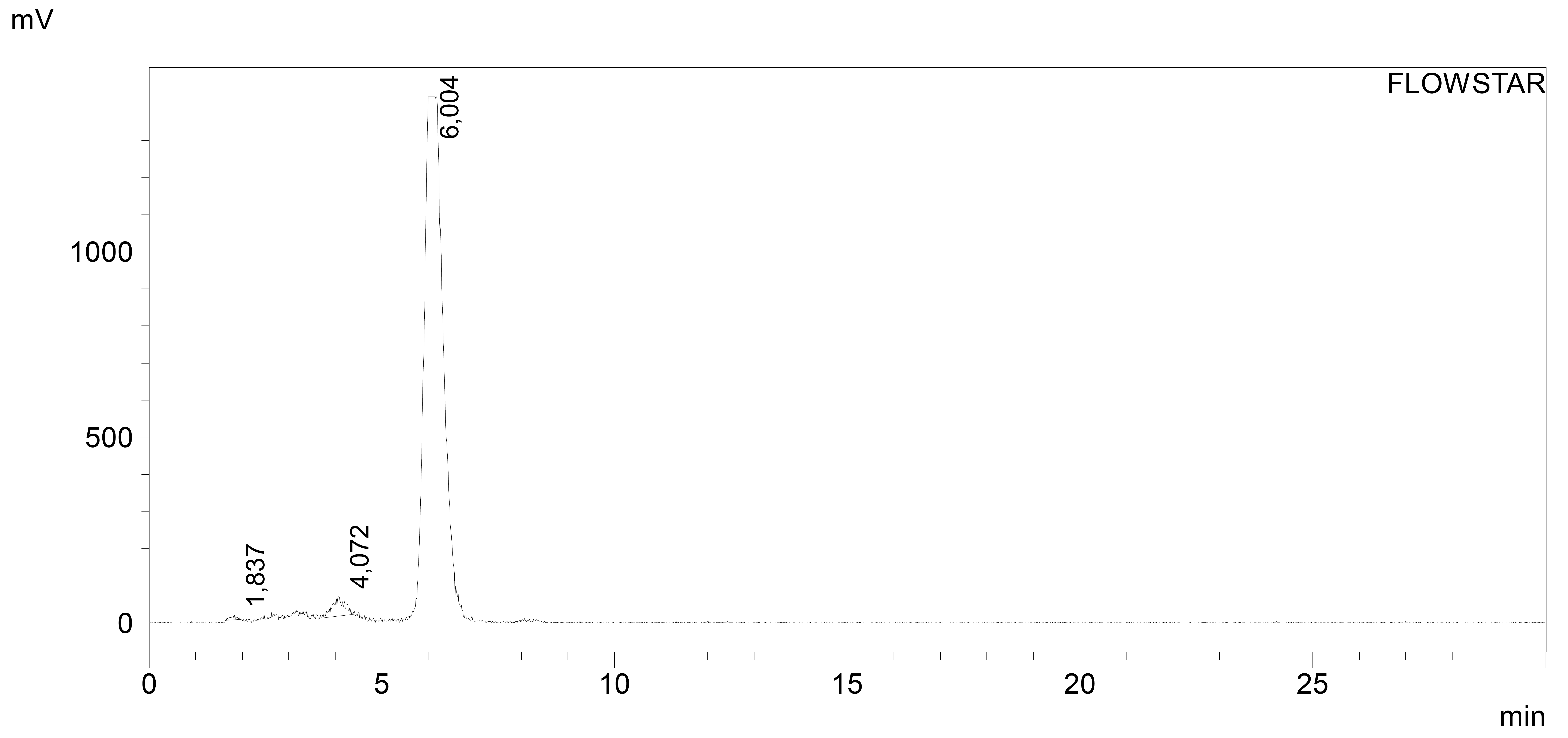


**Figure S29**. Confirmation of radiopeptide identity and purity by co-injection of ^nat^Lu-DOTA-PP‑F11N (left, t_R_ = 5.795 min) with [^177^Lu]Lu‑DOTA‑PP‑F11N (right, t_R_ = 6.004 min), 30-50% B in 20 min (Method A); RCP: 97.3%.


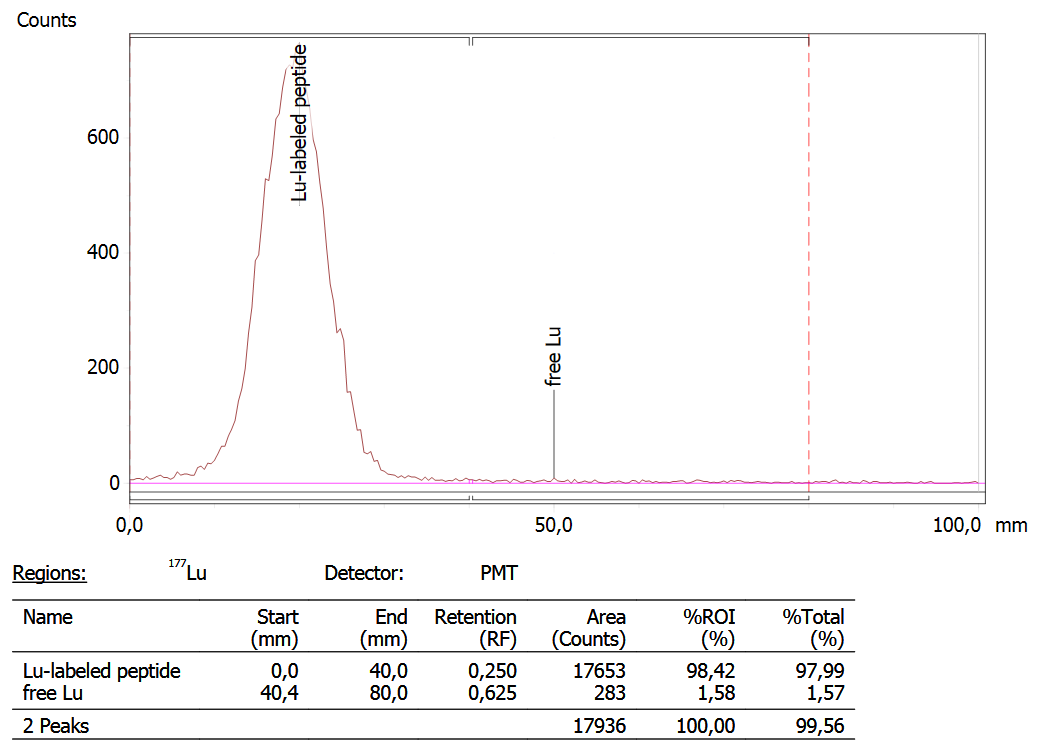

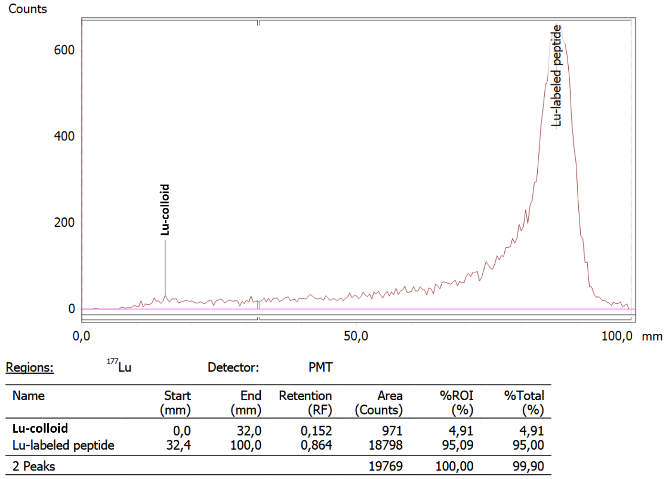


**Figure S30.** Radio‑TLC chromatograms of [^177^Lu]Lu-DOTA-PP‑F11N, left: disodium citrate sesquihydrate (0.1 M, aq.) on ITLC‑SG paper, RCP: 98.4%; right: MeCN/H_2_O (80/20, + 5% TFA) on Whatman 31 ET chromatography paper, RCP: 95.1%.

**[^177^Lu]Lu-DOTA-CCK-66 ([^177^Lu]Lu‑4)**


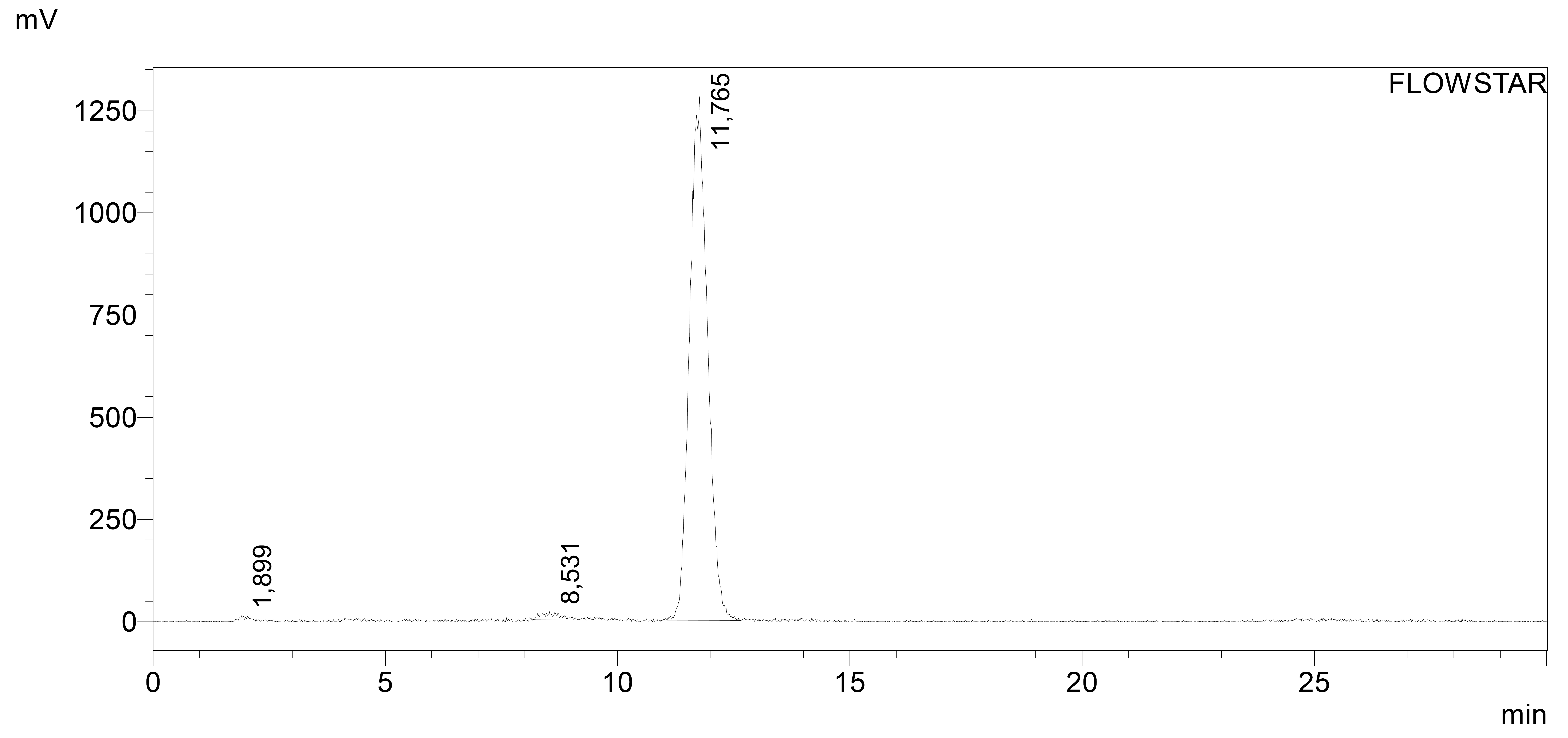


**Figure S31.** Radio‑HPLC chromatogram of [^177^Lu]Lu-DOTA-CCK‑66, 30-50% B in 20 min (Method A); RCP: 98.6%.


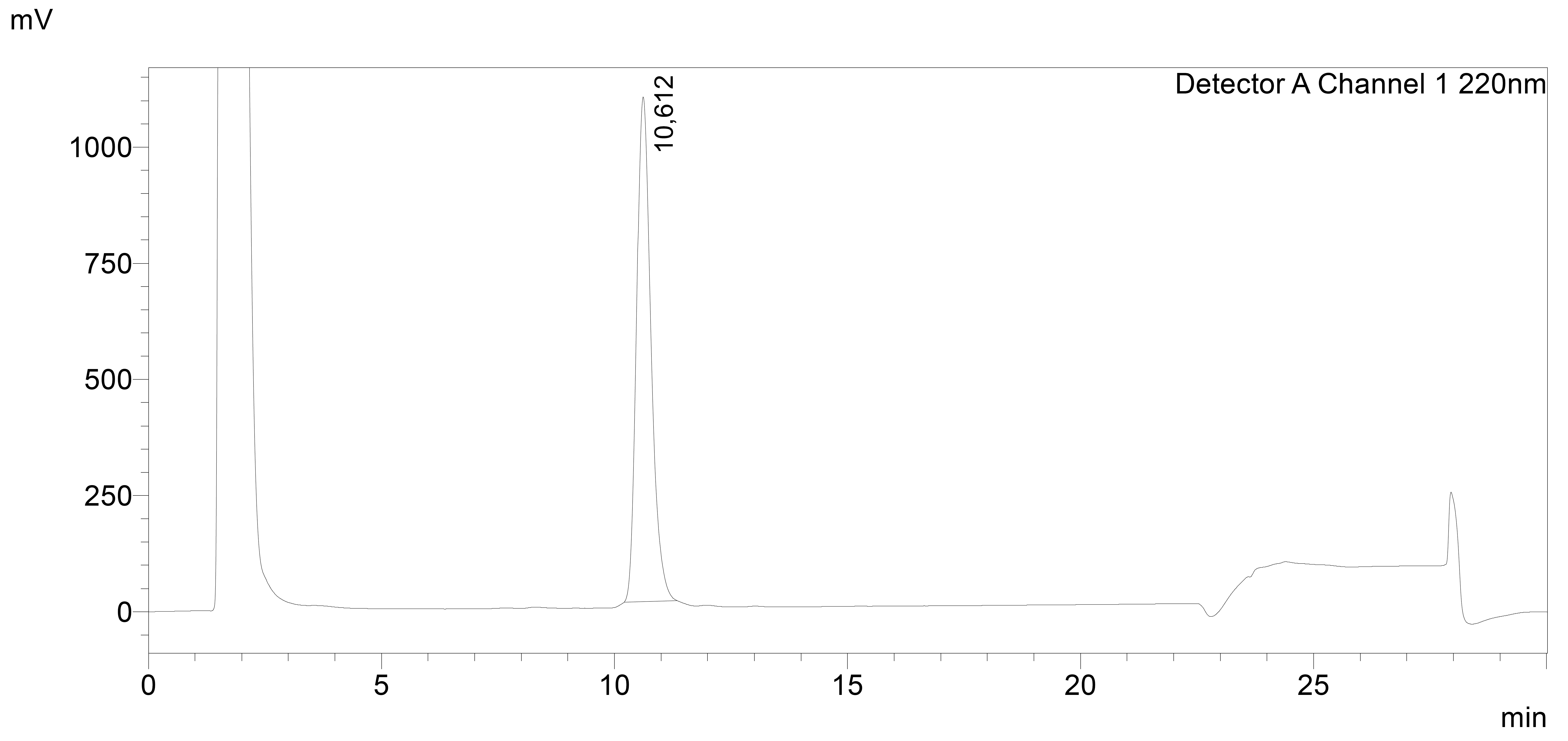

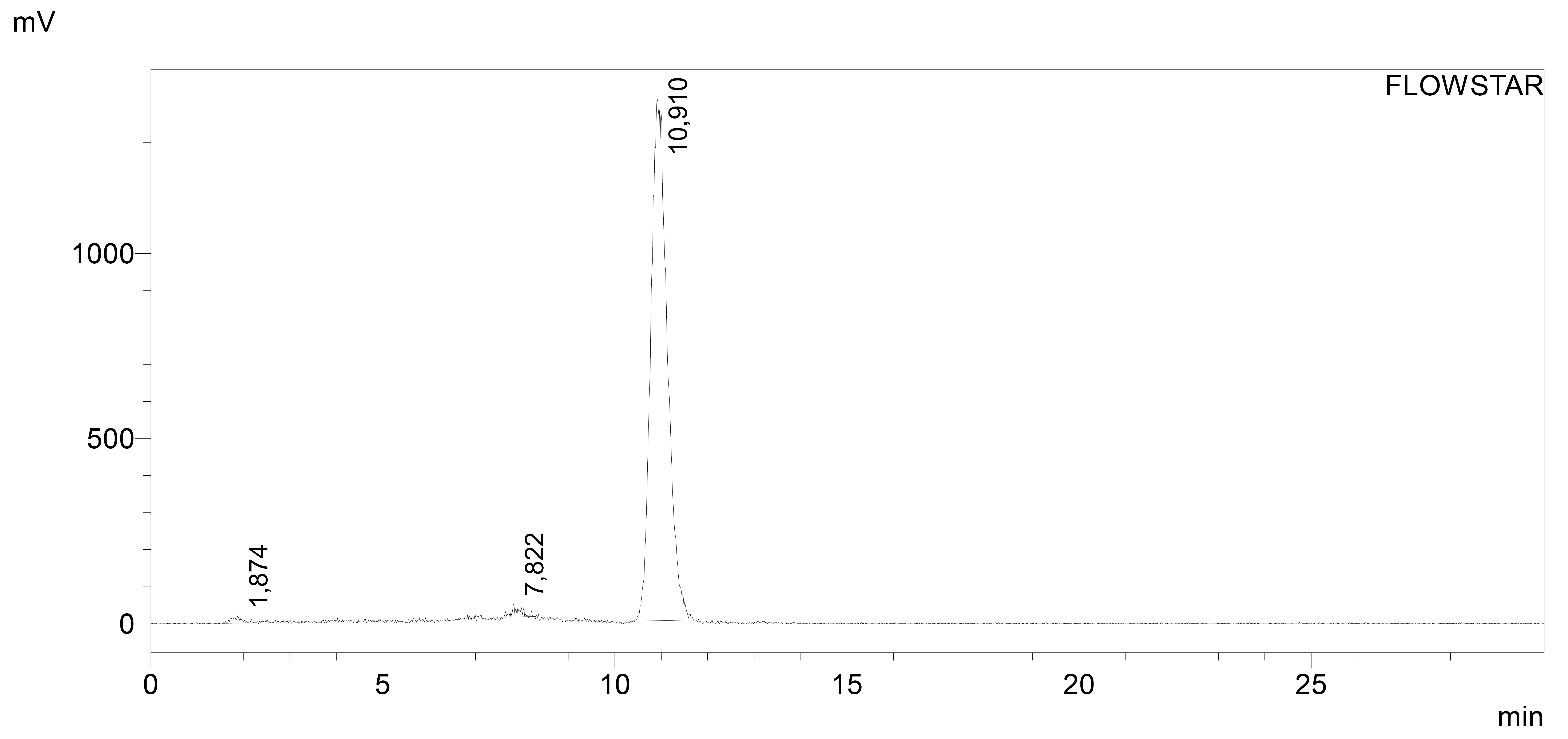


**Figure S32.** Confirmation of radiopeptide identity and purity by co-injection of ^nat^Lu-DOTA-CCK‑66 (left, t_R_ = 10.612 min) with [^177^Lu]Lu‑DOTA‑CCK‑66 (right, t_R_ = 10.910 min), 30-50% B in 20 min (Method A); RCP: 98.1%.


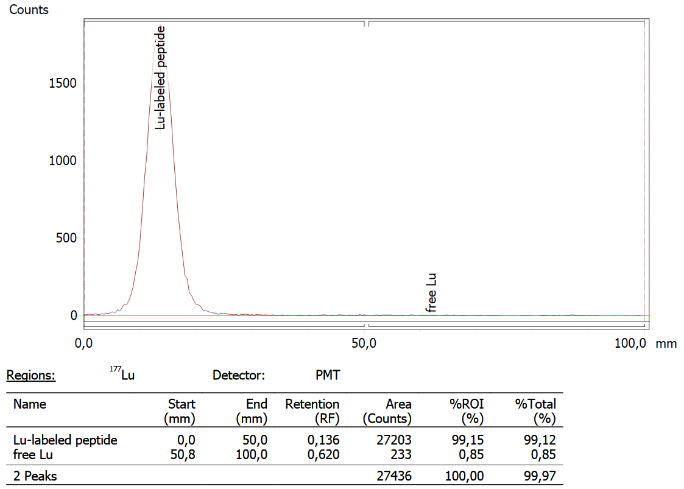

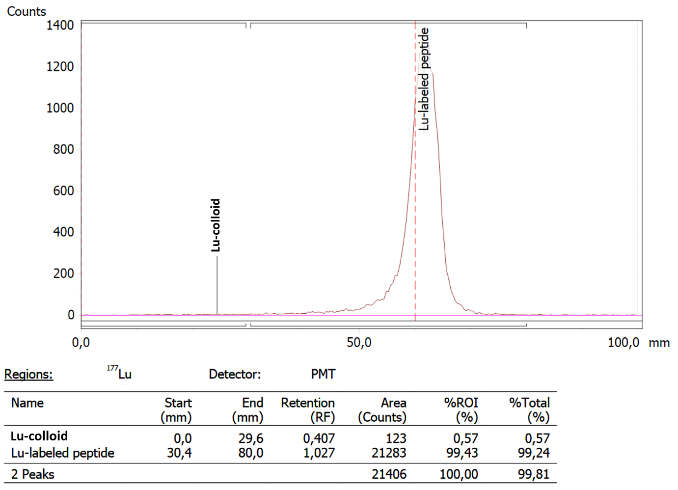


**Figure S33.** Radio‑TLC chromatograms of [^177^Lu]Lu-DOTA-CCK-66, left: disodium citrate sesquihydrate (0.1 M, aq.) on ITLC‑SG paper, RCP: 99.2%; right: MeCN/H_2_O (80/20, + 5% TFA) on Whatman 1 chromatography paper, RCP: 99.4%.


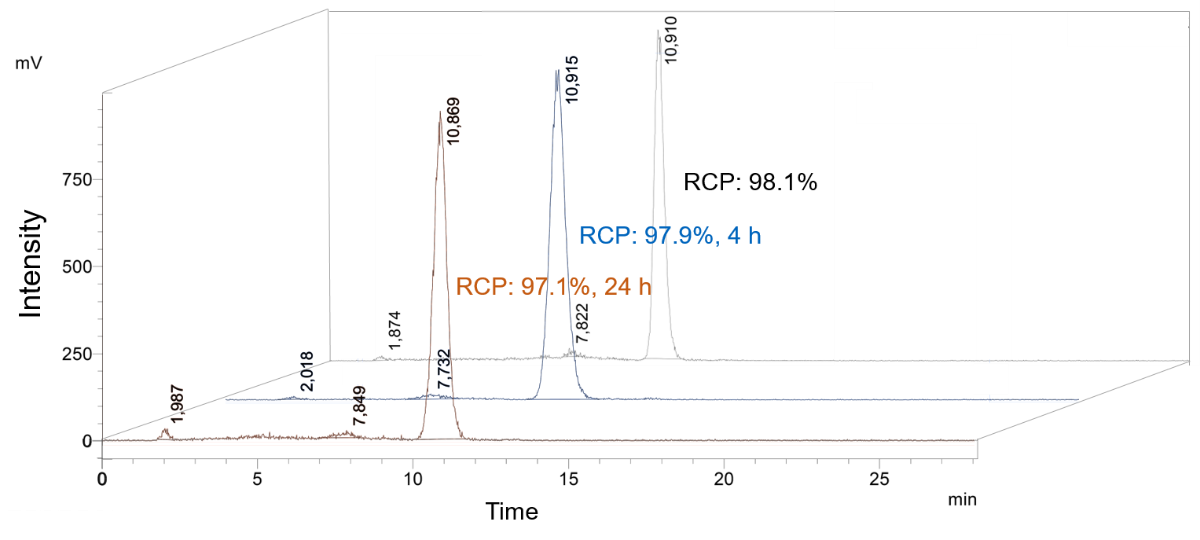


**Figure S34.** Stability of [^177^Lu]Lu-DOTA-CCK-66 in human serum (37 °C, 4 h and 24 h) as analyzed by radio‑HPLC (30 ‑ 50% B in 20 min, Method A). The quality control of the intact compound at end of synthesis (EOS) is depicted in black (rear chromatogram), while the HPLC run after 4 h incubation in human serum is depicted in blue (middle chromatogram) and the HPLC run after 24 h incubation in human serum is depicted in orange (anterior chromatogram).

**[^99m^Tc]Tc-N_4_-CCK-66 ([^99m^Tc]Tc‑11)**


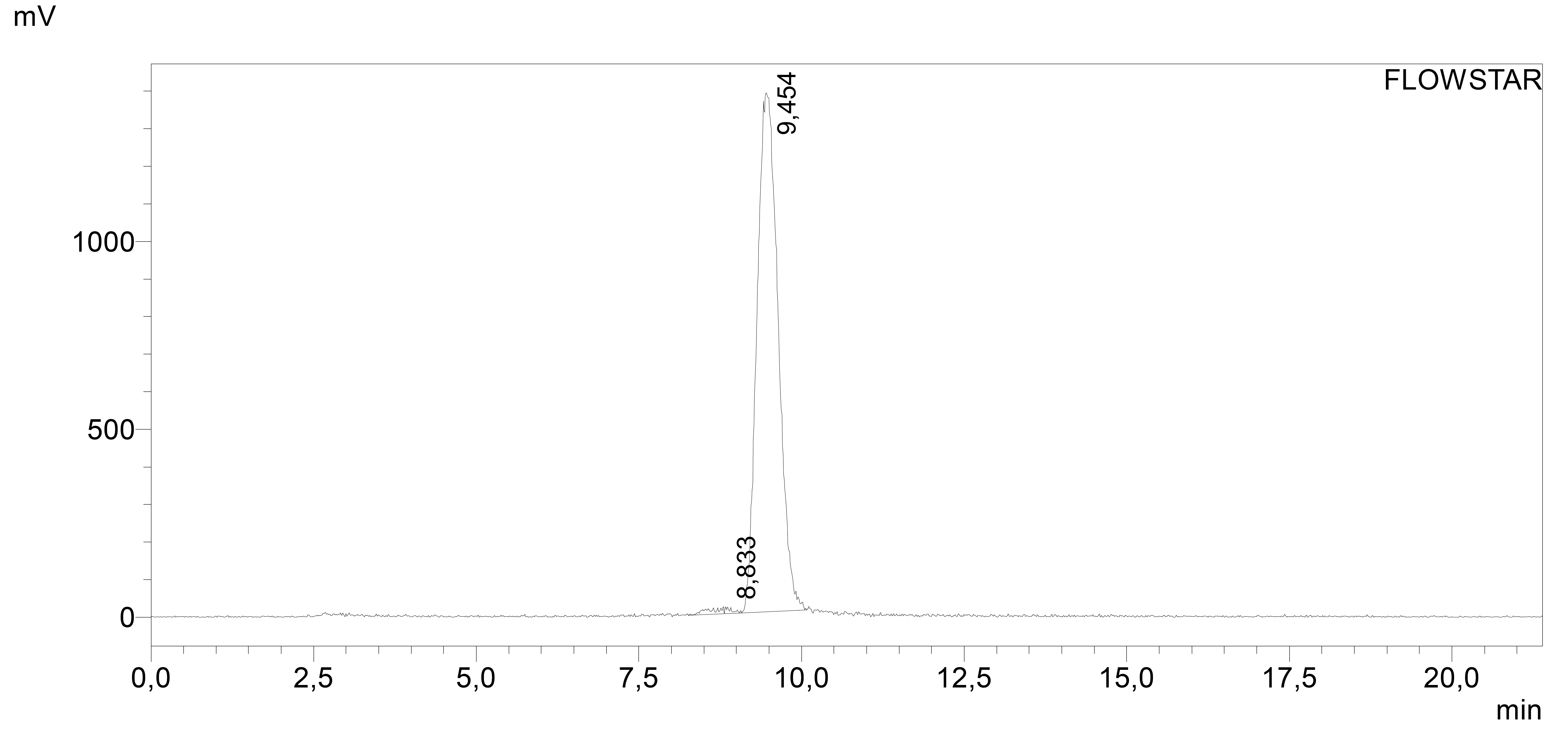


**Figure S35.** Radio‑HPLC chromatogram of [^99m^Tc]Tc-N_4_-CCK‑66, 20-80% B in 15 min (Method A); RCP: 98.7%.

**
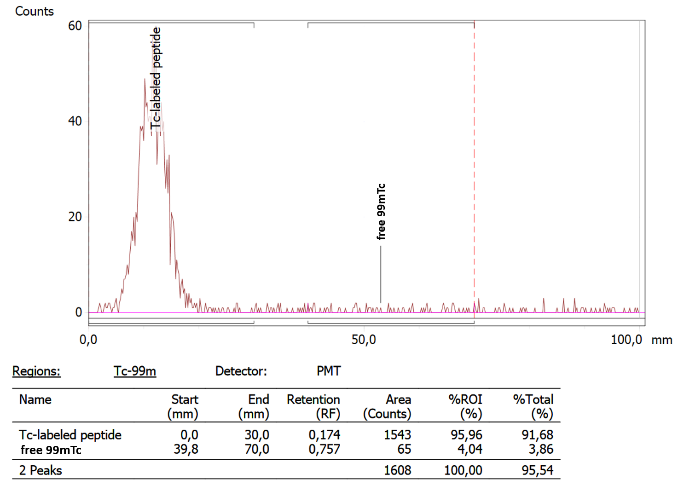

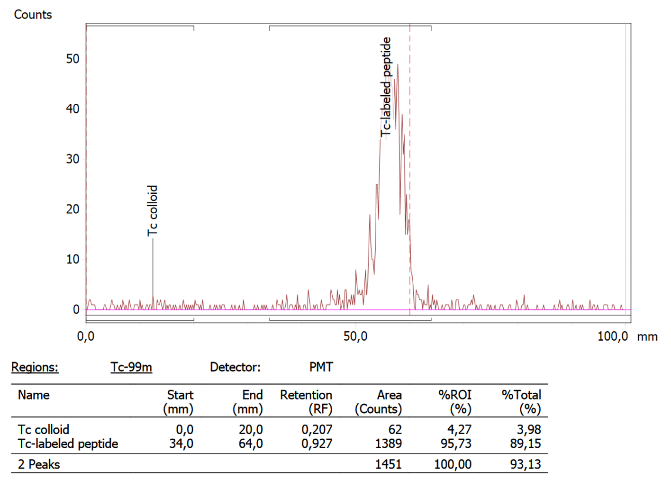
**

**Figure S36.** Radio‑TLC chromatograms of [^99m^Tc]Tc-N_4_-CCK-66, left: MEK on Whatman 1 chromatography paper, RCP: 96.0%; right: MeCN/H_2_O (80/20, + 5% TFA) on Whatman 1 chromatography paper, RCP: 95.7%.


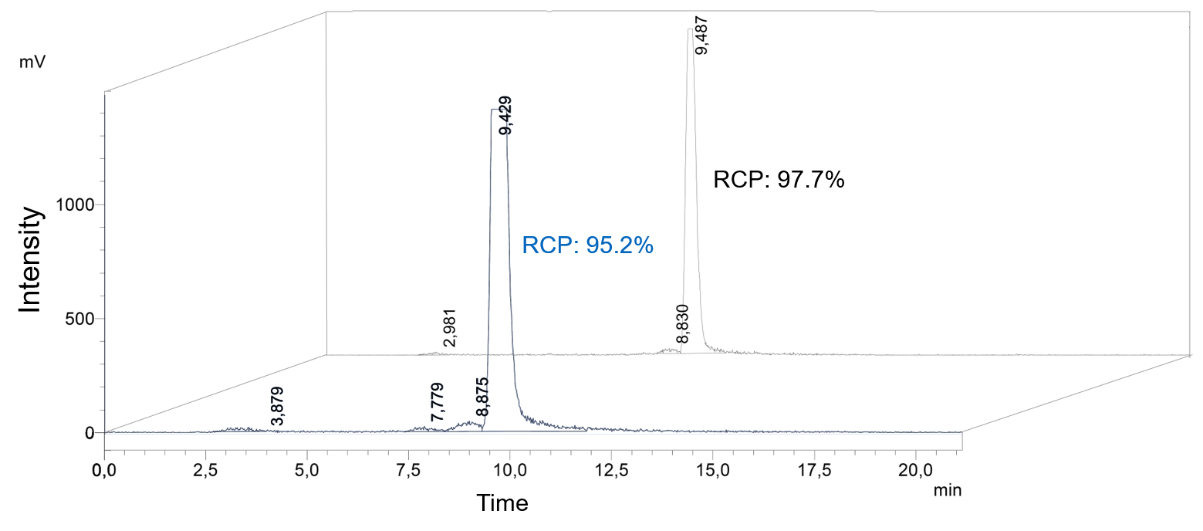


**Figure S37.** Stability of [^99m^Tc]Tc-N_4_-CCK-66 in human serum (37 °C, 4 h) as analyzed by radio‑HPLC (20-80% B in 15 min, Method A). The quality control of the intact compound at EOS is depicted in black (rear chromatogram), while the HPLC run after incubation in human serum is depicted in blue (anterior chromatogram).

**[^99m^Tc]Tc-N_4_-CCK-100 ([^99m^Tc]Tc‑12)**


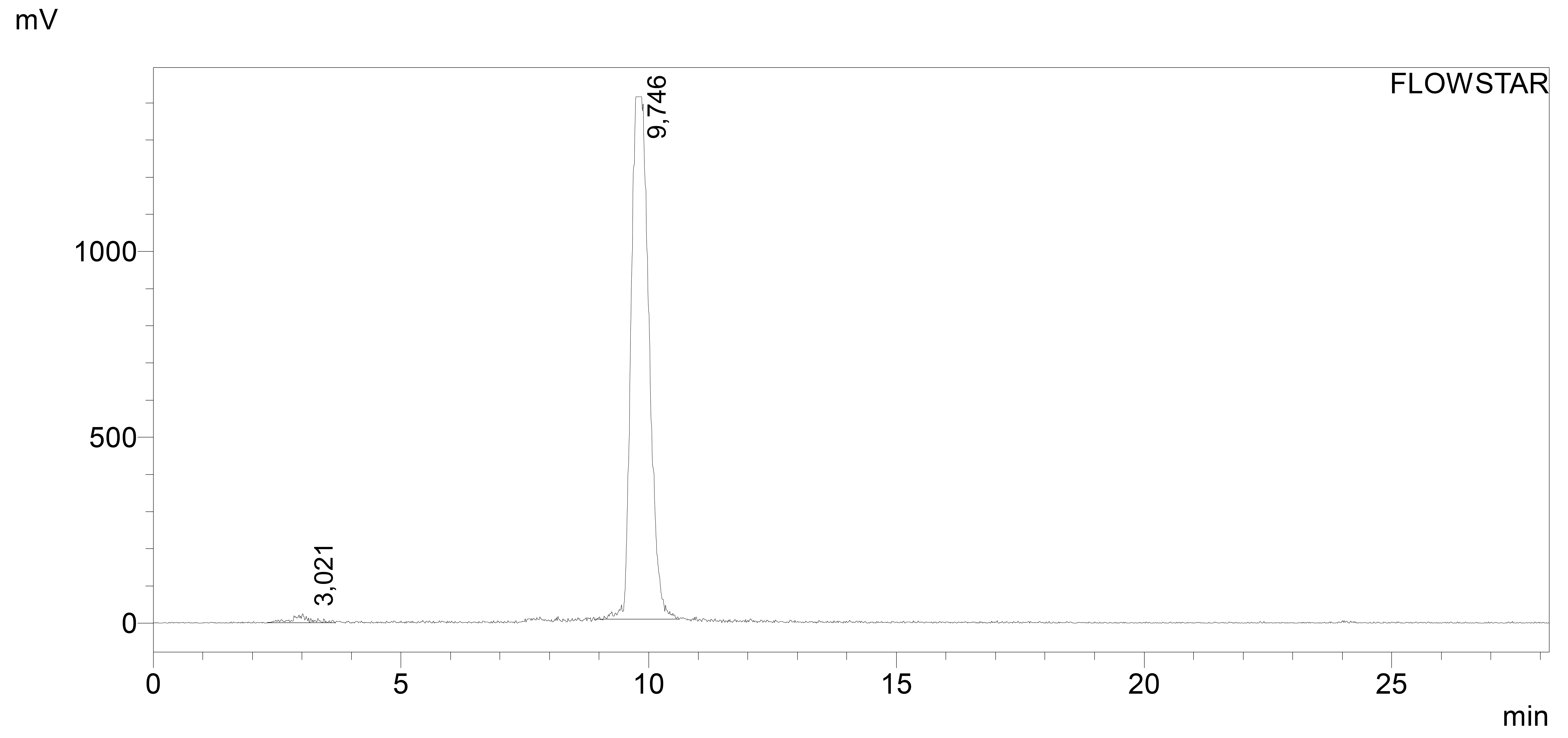


**Figure S38.** Radio‑HPLC chromatogram of [^99m^Tc]Tc-N_4_-CCK‑100, 20-80% B in 15 min (Method A); RCP: 98.4%.


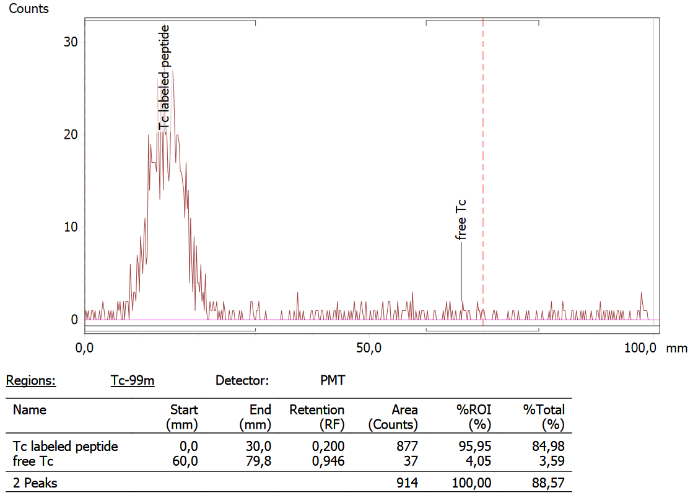

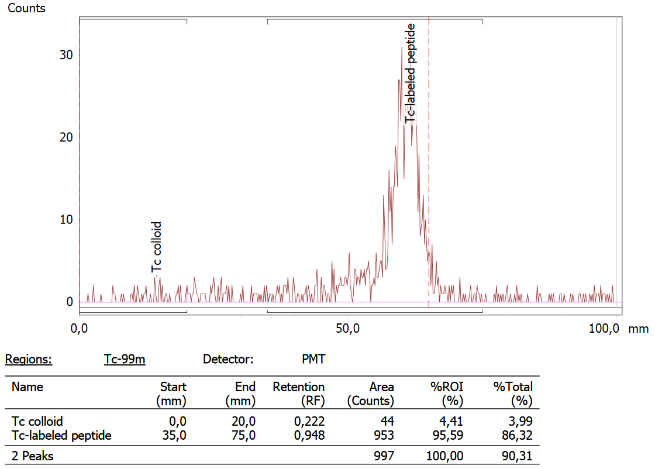


**Figure S39.** Radio‑TLC chromatograms of [^99m^Tc]Tc-N_4_-CCK‑100, left: MEK on Whatman 1 chromatography paper, RCP: 96.0%; right: MeCN/H_2_O (80/20, + 5% TFA) on Whatman 1 chromatography paper, RCP: 95.6%.


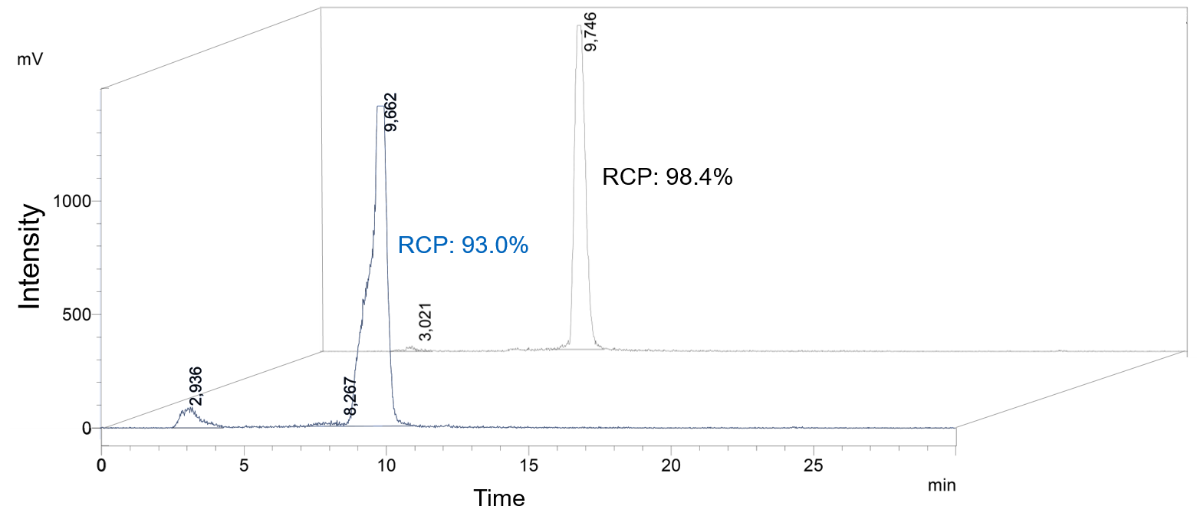


**Figure S40.** Stability of [^99m^Tc]Tc-N_4_-CCK-100 in human serum (37 °C, 4 h) as analyzed by radio‑HPLC (20-80% B in 15 min, Method A). The quality control of the intact compound at EOS is depicted in black (rear chromatogram), while the HPLC run after incubation in human serum is depicted in blue (anterior chromatogram).

**[^99m^Tc]Tc-N_4_-CCK-101 ([^99m^Tc]Tc‑13)**


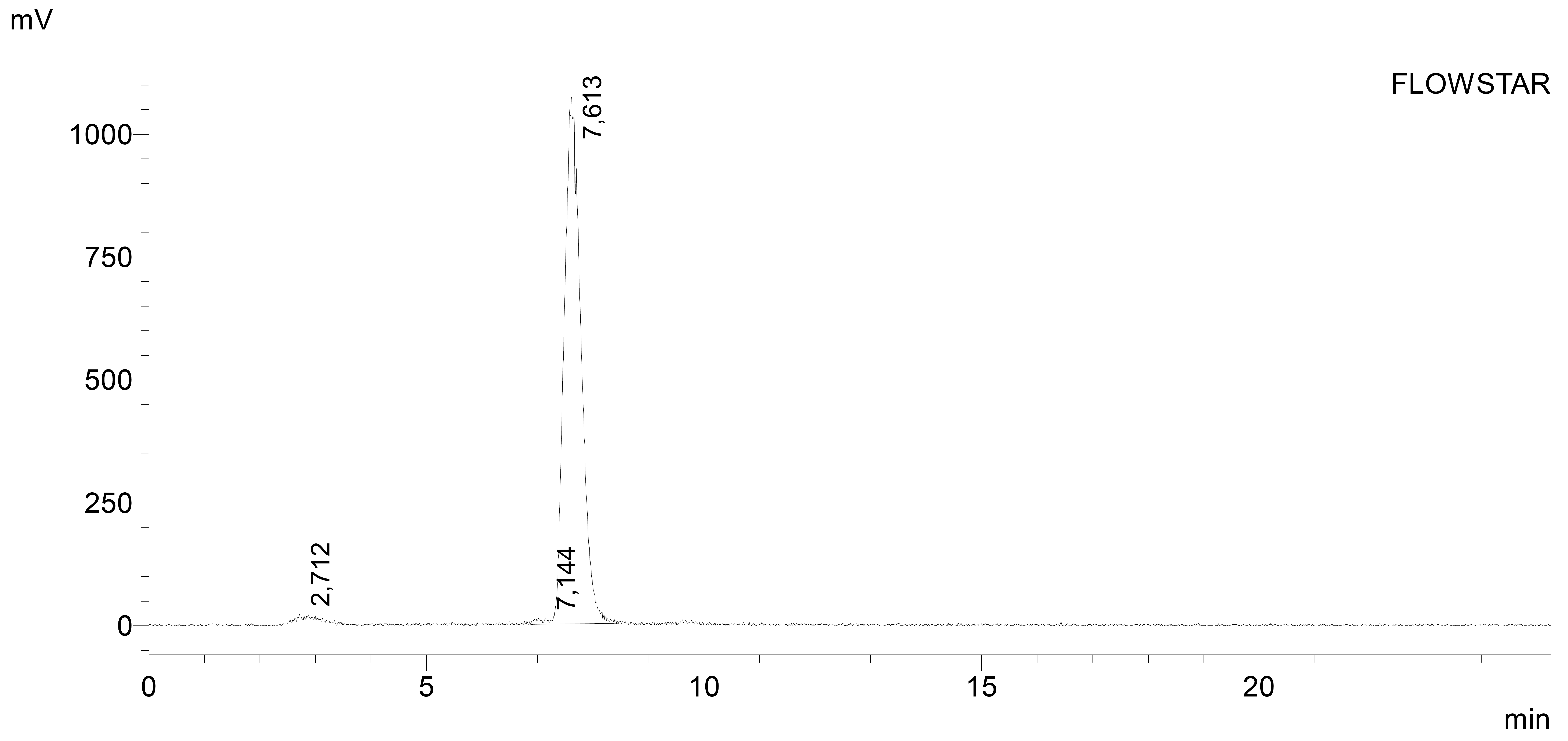


**Figure S41.** Radio‑HPLC chromatogram of [^99m^Tc]Tc-N_4_-CCK‑101, 20-80% B in 15 min (Method A); RCP: 97.3%.


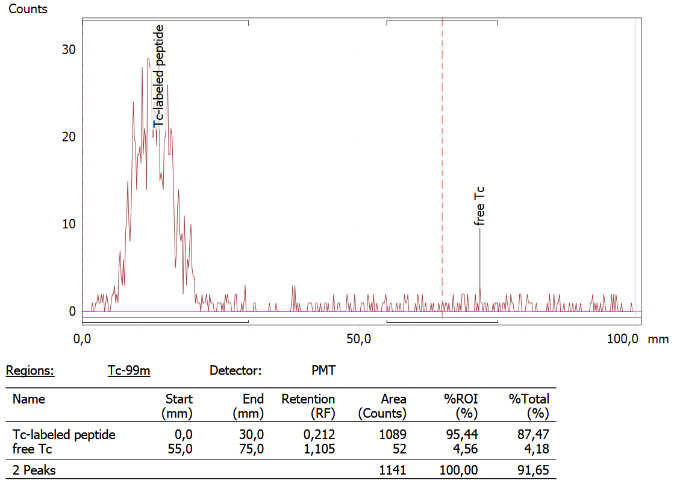

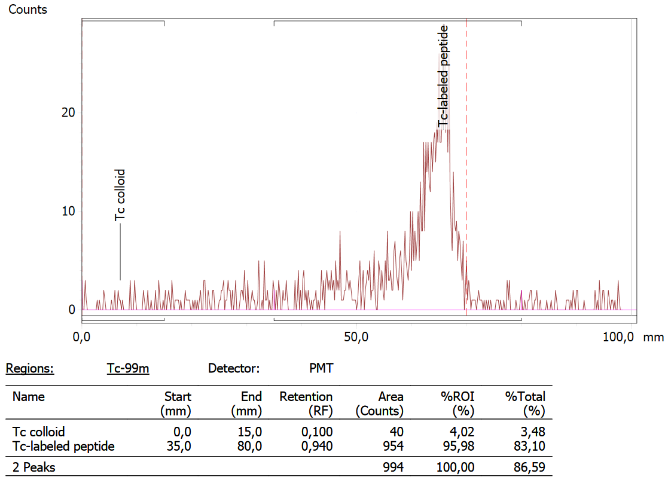


**Figure S42.** Radio‑TLC chromatograms of [^99m^Tc]Tc-N_4_-CCK‑101, left: MEK on Whatman 1 chromatography paper, RCP: 95.4%; right: MeCN/H_2_O (80/20, + 5% TFA) on Whatman 1 chromatography paper, RCP: 96.0%.


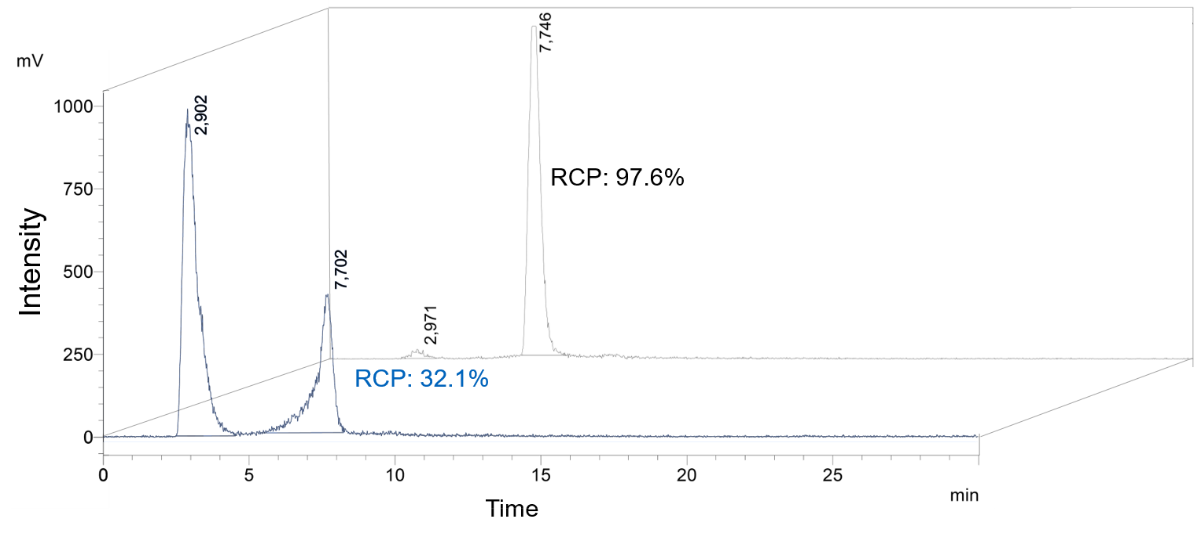


**Figure S43.** Stability of [^99m^Tc]Tc-N_4_-CCK-101 in human serum (37 °C, 4 h) as analyzed by radio‑HPLC (20-80% B in 15 min, Method A). The quality control of the intact compound at EOS is depicted in black (rear chromatogram), while the HPLC run after incubation in human serum is depicted in blue (anterior chromatogram).

**[^99m^Tc]Tc-N_4_-CCK-102 ([^99m^Tc]Tc‑14)**


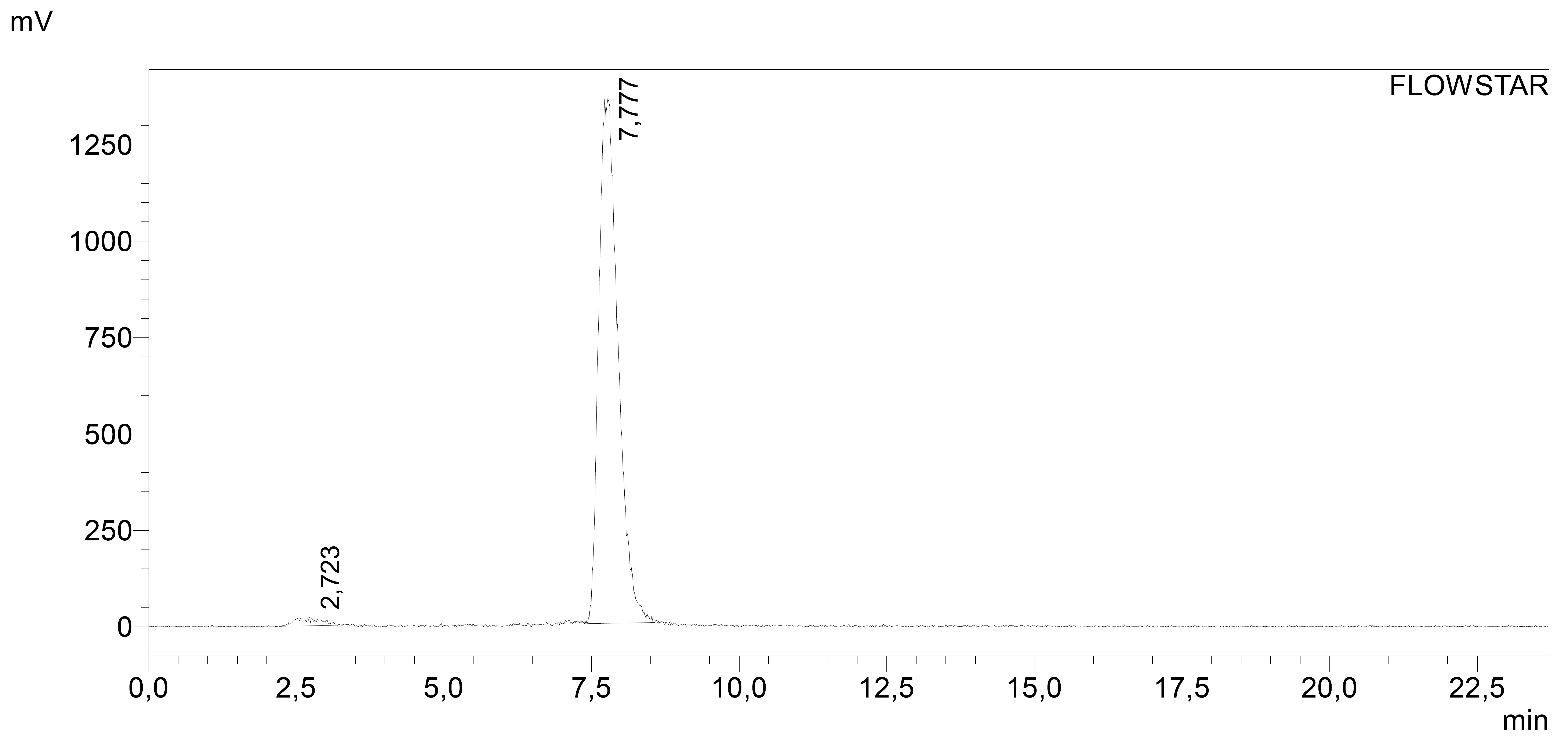


***Figure S44*.** Radio‑HPLC chromatogram of [^99m^Tc]Tc-N_4_-CCK‑102, 20-80% B in 15 min (Method A); RCP: 98.0%.

*
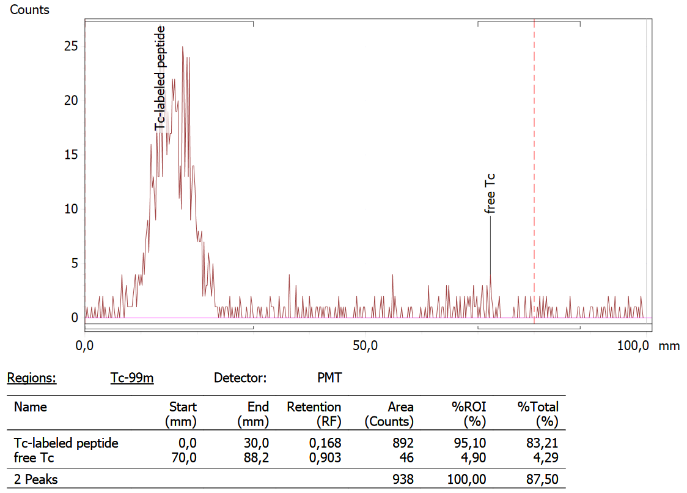

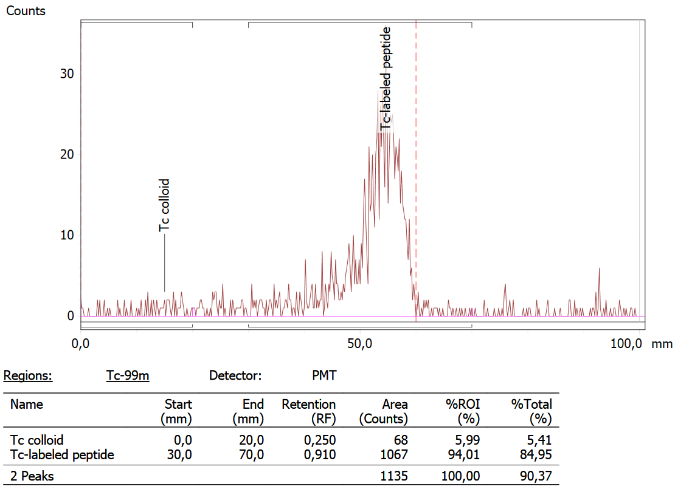
*

**Figure S45.** Radio‑TLC chromatograms of [^99m^Tc]Tc-N_4_-CCK‑102, left: MEK on Whatman 1 chromatography paper, RCP: 95.1%; right: MeCN/H_2_O (80/20, + 5% TFA) on Whatman 1 chromatography paper, RCP: 94.0%.


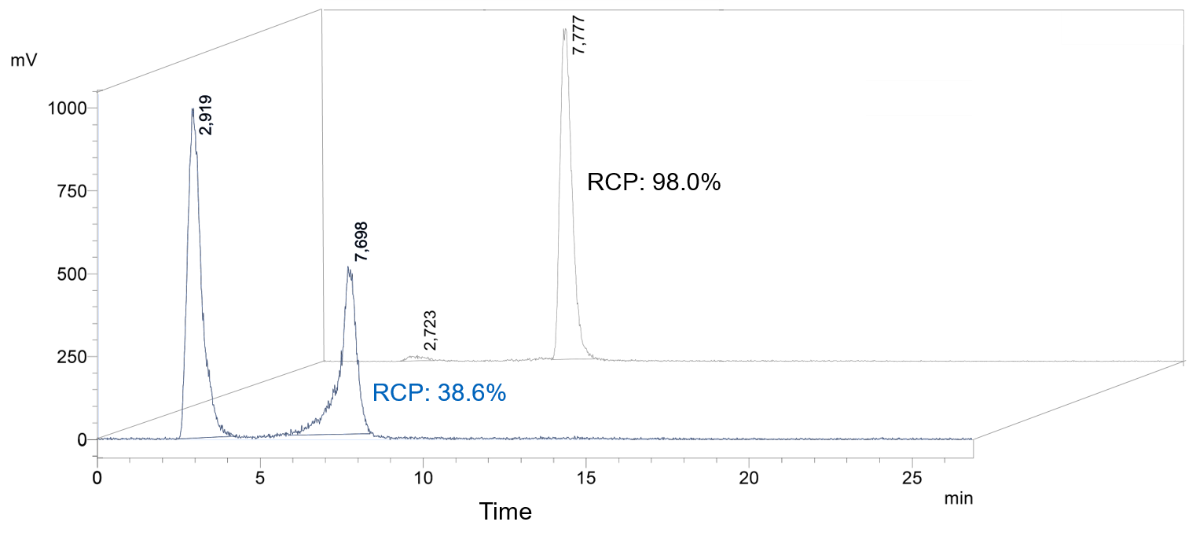


**Figure S46.** Stability of [^99m^Tc]Tc-N_4_-CCK-102 in human serum (37 °C, 4 h) as analyzed by radio‑HPLC (20-80% B in 15 min, Method A). The quality control of the intact compound at EOS is depicted in black (rear chromatogram), while the HPLC run after incubation in human serum is depicted in blue (anterior chromatogram).

**[^99m^Tc]Tc-N_4_-CCK-103 ([^99m^Tc]Tc‑15)**


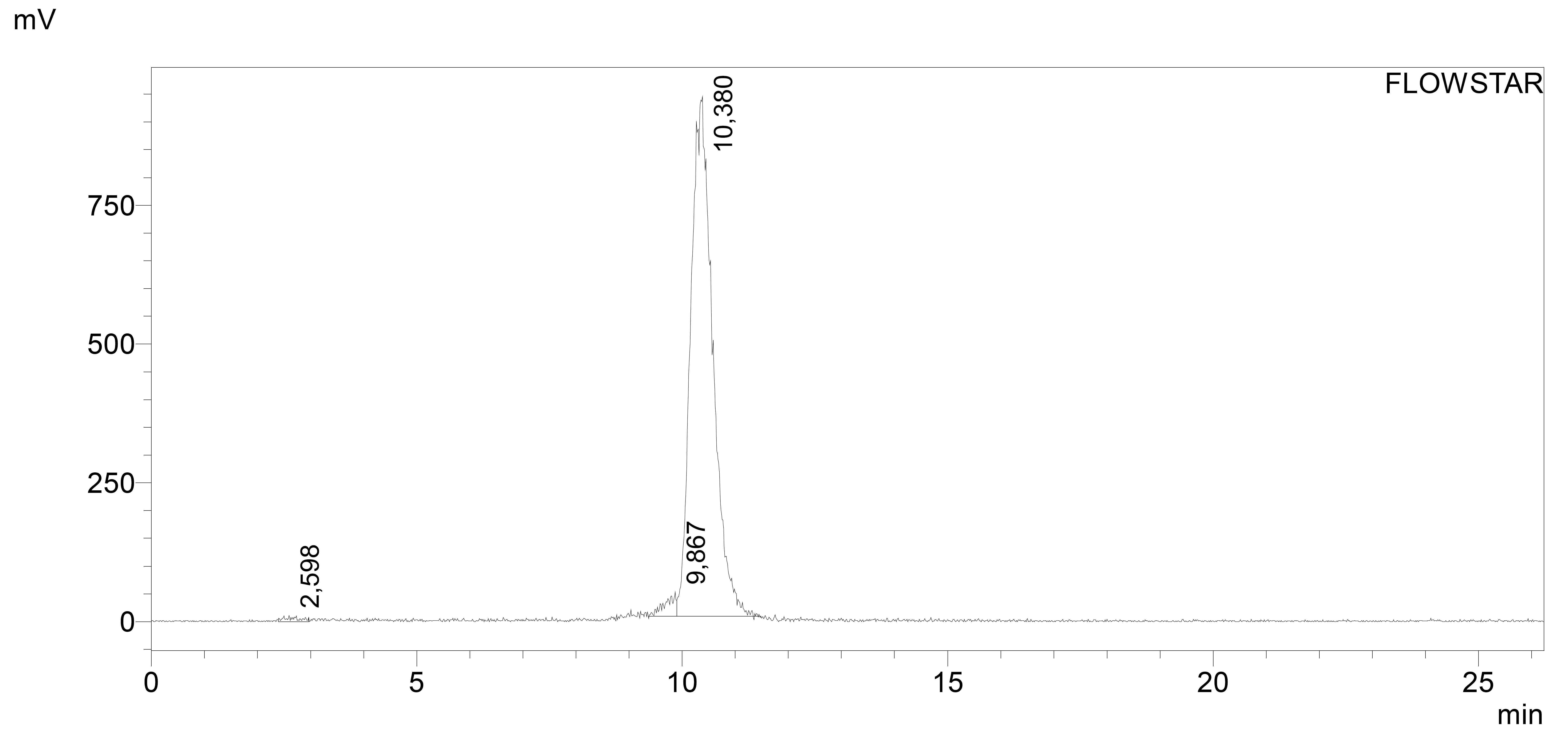


***Figure S47*.** Radio‑HPLC chromatogram of [^99m^Tc]Tc-N_4_-CCK‑103, 20-80% B in 15 min (Method A); RCP: 97.2%.

**
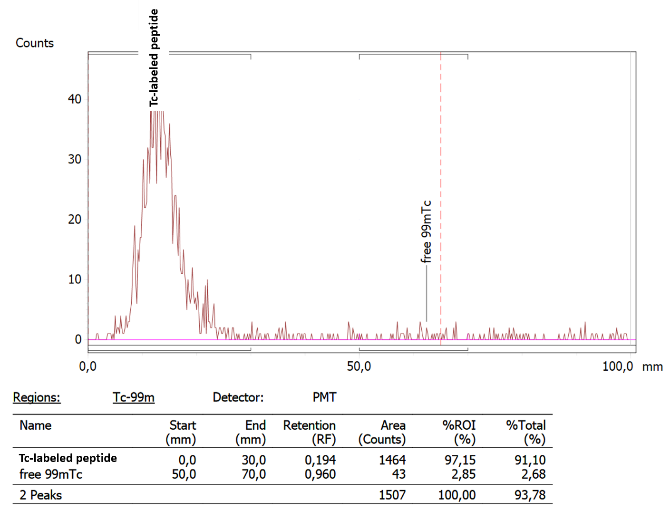

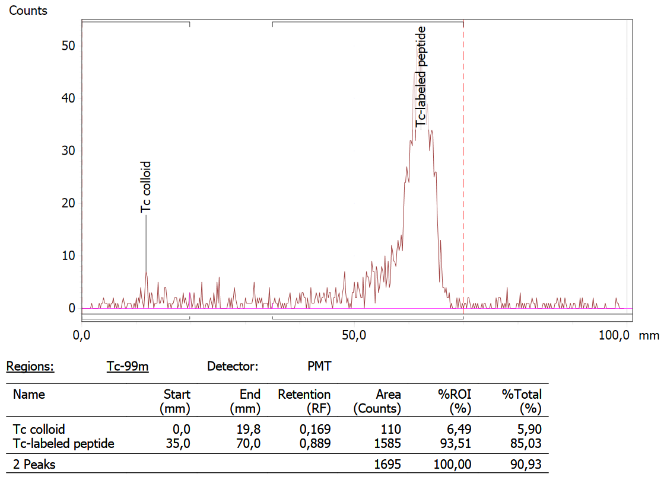
**

**Figure S48.** Radio‑TLC chromatograms of [^99m^Tc]Tc-N_4_-CCK‑103, left: MEK on Whatman 1 chromatography paper, RCP: 97.2%; right: MeCN/H_2_O (80/20, + 5% TFA) on Whatman 1 chromatography paper, RCP: 93.5%.


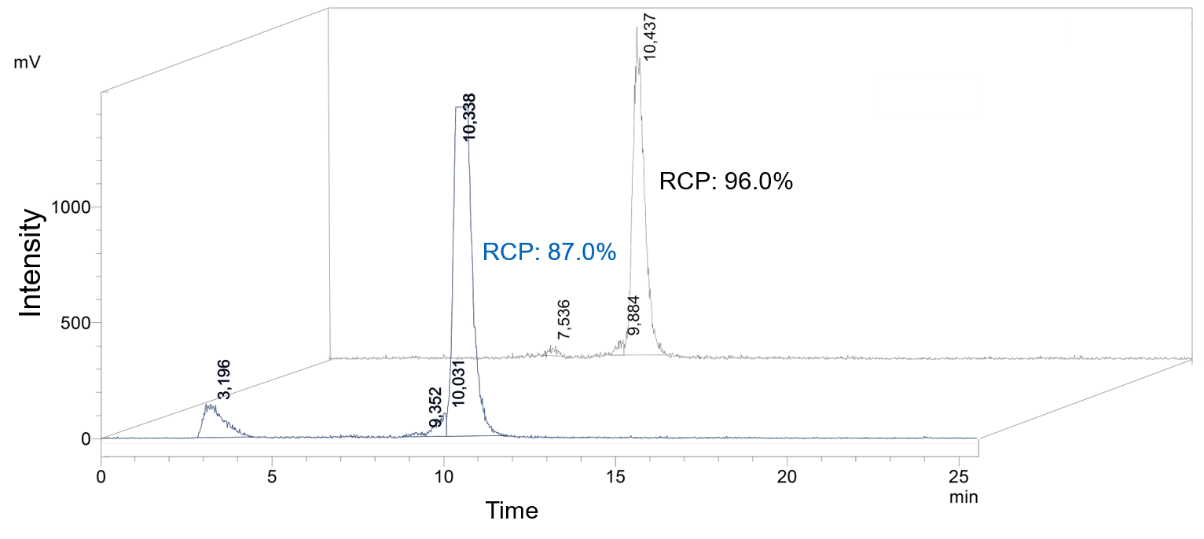


**Figure S49.** Stability of [^99m^Tc]Tc-N_4_-CCK-103 in human serum (37 °C, 4 h) as analyzed by radio‑HPLC (20-80% B in 15 min, Method A). The quality control of the intact compound at EOS is depicted in black (rear chromatogram), while the HPLC run after incubation in human serum is depicted in blue (anterior chromatogram).

**[^99m^Tc]Tc-N_4_-CCK-104 ([^99m^Tc]Tc‑16)**


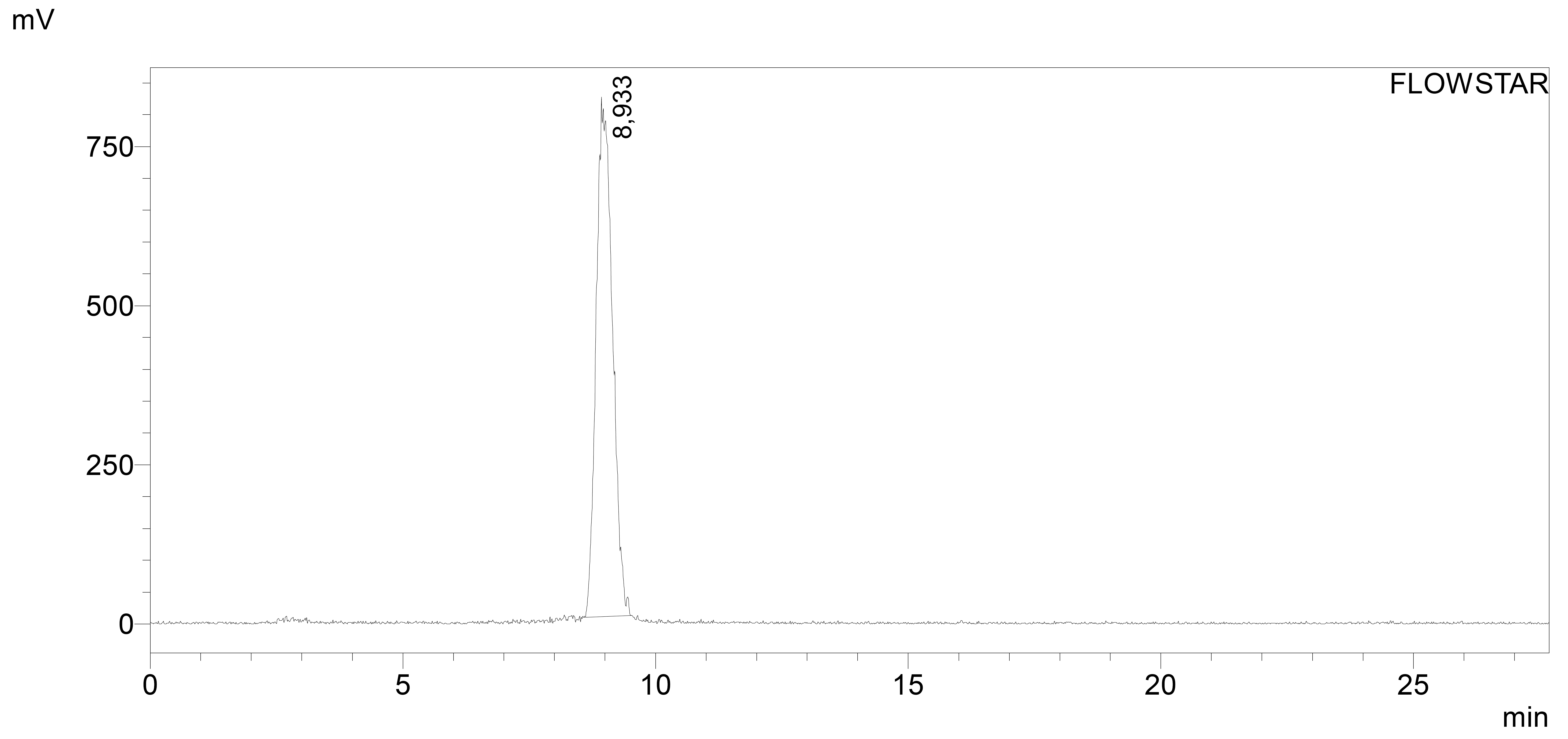


***Figure S50*.** Radio‑HPLC chromatogram of [^99m^Tc]Tc-N_4_-CCK‑104, 20-80% B in 15 min (Method A); RCP: >99%.


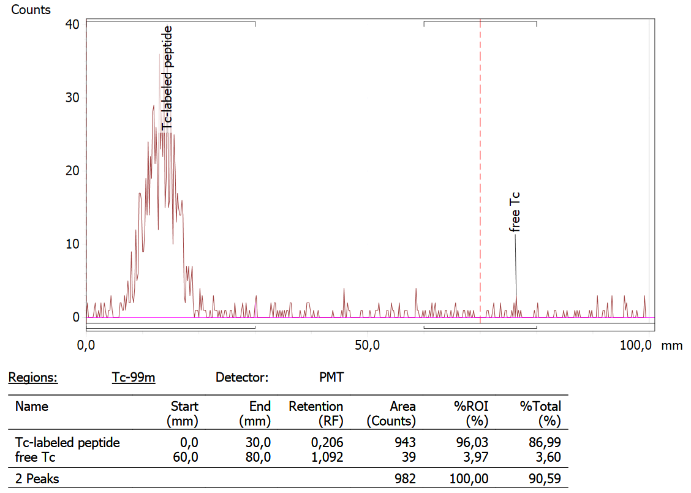

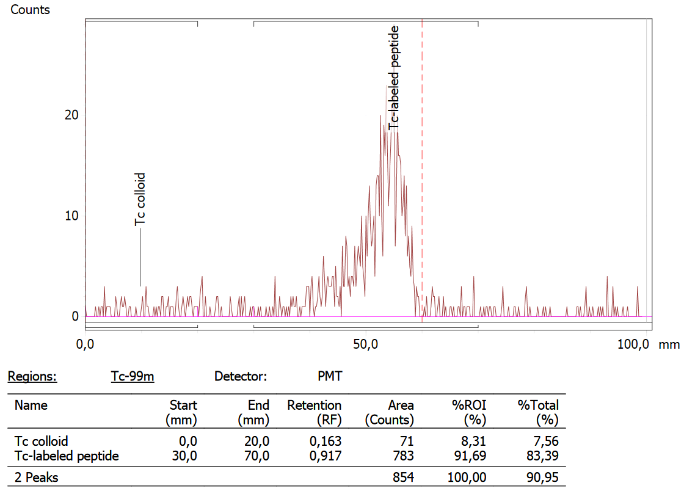


***Figure S51*.** Radio‑TLC chromatograms of [^99m^Tc]Tc-N_4_-CCK‑104, left: MEK on Whatman 1 chromatography paper, RCP: 96.0%; right: MeCN/H_2_O (80/20, + 5% TFA) on Whatman 1 chromatography paper, RCP: 91.7%.


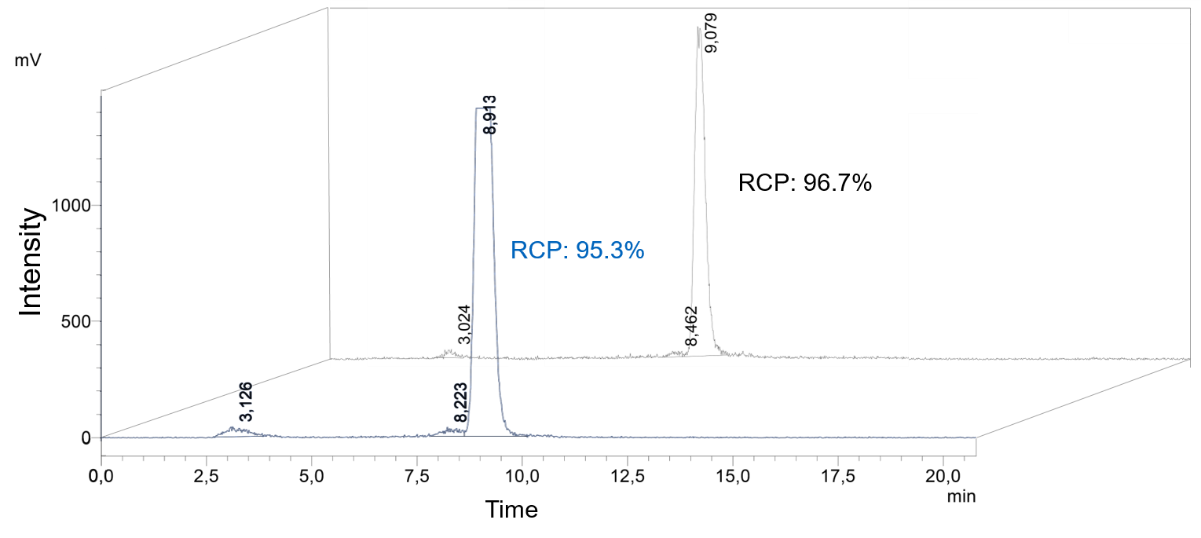


**Figure S52.** Stability of [^99m^Tc]Tc-N_4_-CCK-104 in human serum (37 °C, 4 h) as analyzed by radio‑HPLC (20-80% B in 15 min, Method A). The quality control of the intact compound at EOS is depicted in black (rear chromatogram), while the HPLC run after incubation in human serum is depicted in blue (anterior chromatogram).

**[^99m^Tc]Tc-N_4_-CCK-105 ([^99m^Tc]Tc‑17)**


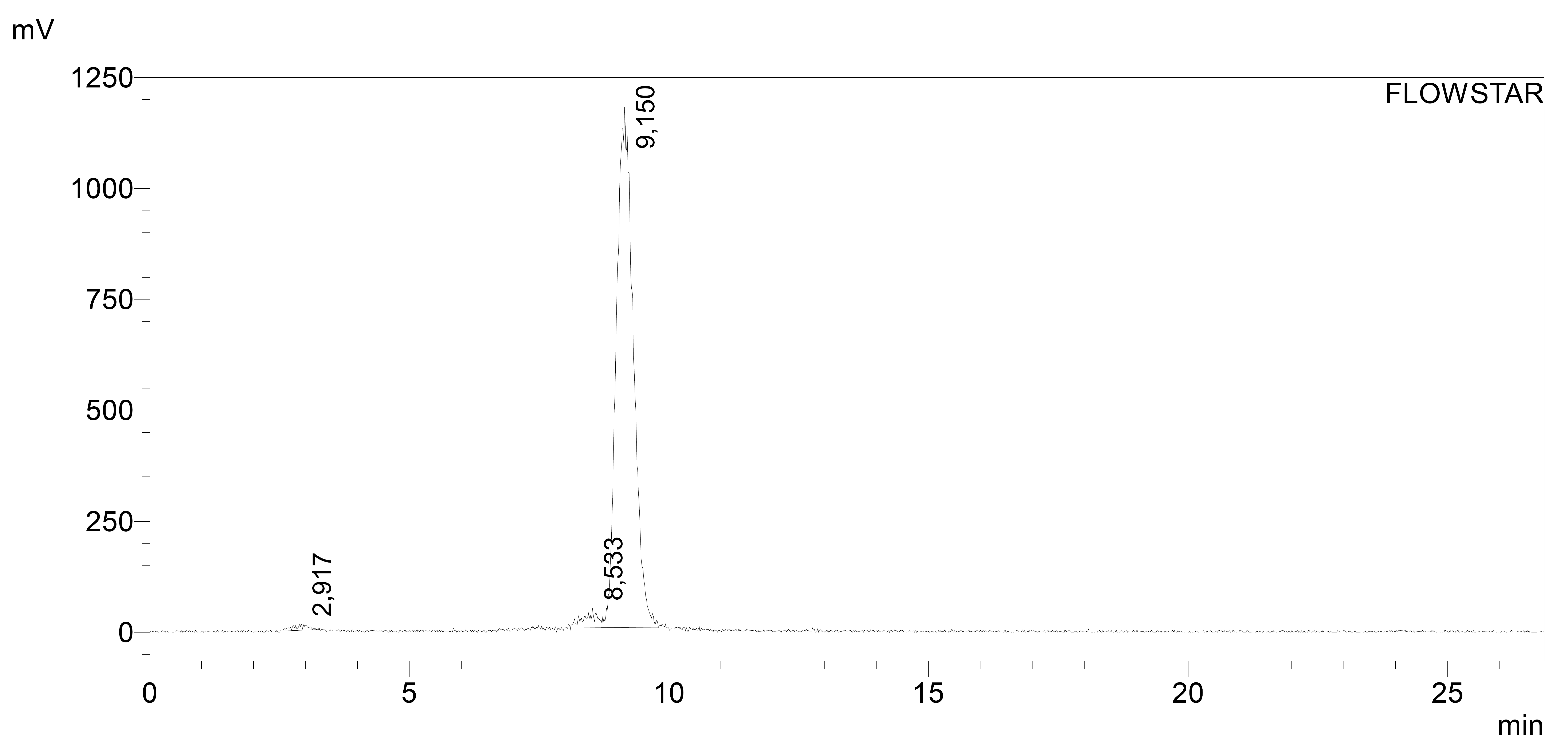


***Figure S53*.** Radio‑HPLC chromatogram of [^99m^Tc]Tc-N_4_-CCK‑105, 20-80% B in 15 min (Method A); RCP: 96.2%.


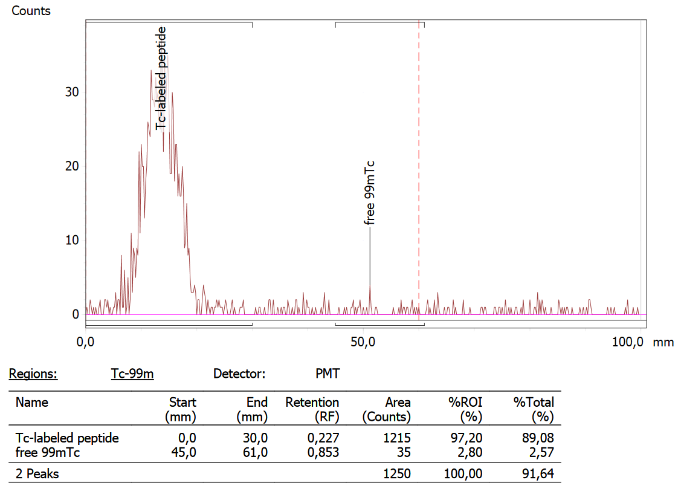

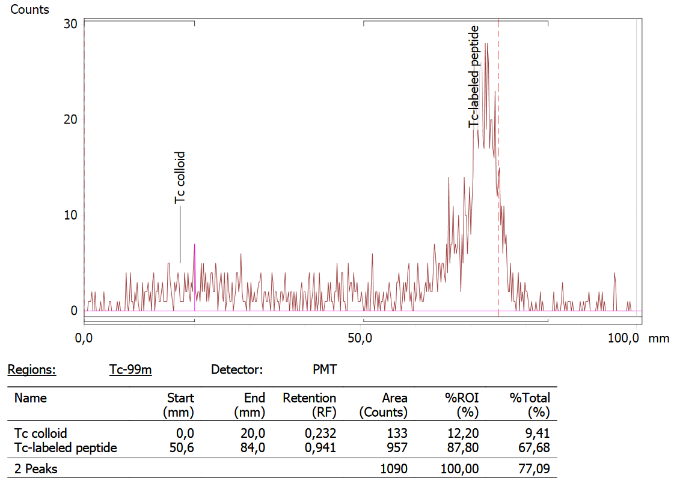


***Figure S54*.** Radio‑TLC chromatograms of [^99m^Tc]Tc-N_4_-CCK‑105, left: MEK on Whatman 1 chromatography paper, RCP: 97.2%; right: MeCN/H_2_O (80/20, + 5% TFA) on Whatman 1 chromatography paper, RCP: 87.8%.


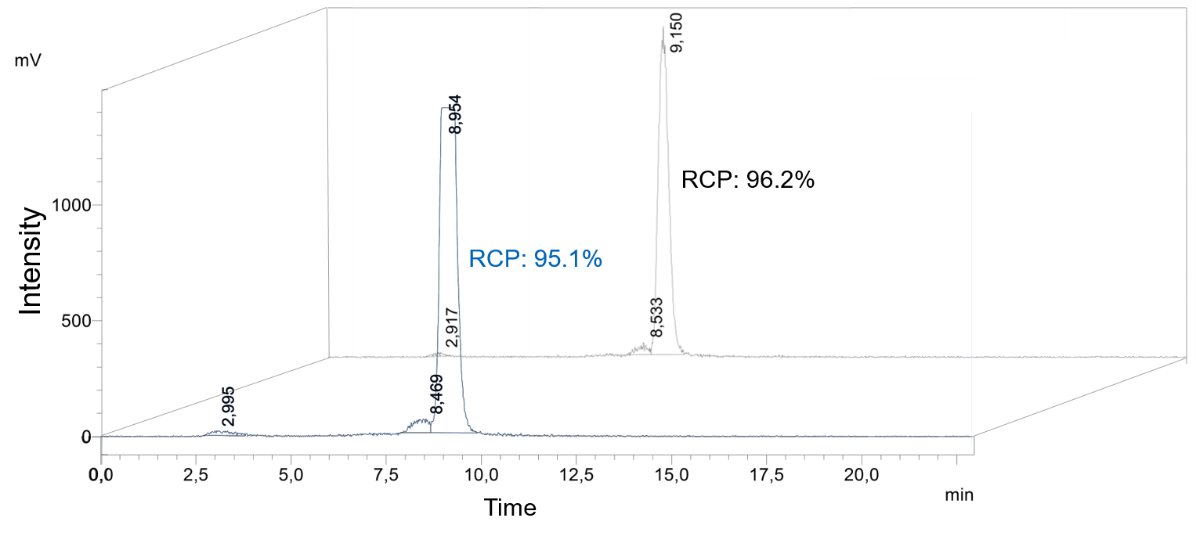


**Figure S55.** Stability of [^99m^Tc]Tc-N_4_-CCK-105 in human serum (37 °C, 4 h) as analyzed by radio‑HPLC (20-80% B in 15 min, Method A). The quality control of the intact compound at EOS is depicted in black (rear chromatogram), while the HPLC run after incubation in human serum is depicted in blue (anterior chromatogram).

- 1. **Results of radio‑TLC**

**Table S1**: radio-TLC experiments of all ^99m^Tc- and ^177^Lu-labelings, in particular to identify a suitable TLC method to properly identify ^99m^Tc-colloids. RCPs of radio‑TLC ≥95% are highlighted in green, RCPs ≥80% and <95% are highlighted in yellow, RCPs <80% are highlighted in red. ^a^TLC aluminum sheets, coated with silica gel 60 RP-18 F254s; ^b^Munktell 120 204 Grade 1288 filter paper; ^c^VWR grade 303 filter paper; ^d^TLC aluminum sheets, coated with silica gel 60; ^e^0.1 M sodium citrate*1.5 H_2_O (aq.); ^f^[^99m^Tc]TcO_4_^-^ from generator eluate; ^g^RCP measured at R_f_= 0.9-1.0.

| **No. of experi-ment** | **Compound** | **RCP [%] (radio-RP-HPLC)** | **Detection of free [^99m^Tc]TcO_4_^-^ (*R*_f_ = 0.9‑1.0) and radiolabeled peptide (*R*_f_ = 0.0-0.1)** | | **RCP [%] at**  ***R*_f_ = 0.0-0.1 (radio-TLC)** | **Detection of ^99m^Tc-colloid (*R*_f_ = 0.0) and radiolabeled peptide (*R*_f_ = 0.9-1.0)** | | **RCP [%] at**  ***R*_f_ = 0.9-1.0 (radio-TLC)** |
| --- | --- | --- | --- | --- | --- | --- | --- | --- |
|  |  |  | **Stationary phase** | **Mobile phase** |  | **Stationary phase** | **Mobile phase** |  |
| 1 | [^99m^Tc]Tc-**11** | 97.7 | RP^a^ | MEK | 96.3 | ITLC-SG | NaCl (0.9%, aq.) | 3.7 |
|  |  |  | - | - | - | ITLC-SG | NaCl (1 M, aq.) | 4.3 |
| 2 | [^99m^Tc]Tc-**11** | 98.5 | RP | MEK | 95.8 | ITLC-SG | NaCl (0.9%, aq.) | 3.5 |
|  |  |  | - | - | - | ITLC-SG | NaCl (1 M, aq.) | 4.6 |
| 3 | [^99m^Tc]Tc-**11** | 98.7 | Munktell paper^b^ | MEK | 95.8 | Munktell paper | NaCl (1 M, aq.) | 10.3 |
|  |  |  | VWR paper^c^ | MEK | 94.8 | VWR paper | NaCl (1 M, aq.) | 10.4 |
|  |  |  | ITLC-SG | MEK | 96.6 | ITLC-SG | NaCl (1 M, aq.) | 5.2 |
|  |  |  | RP | MEK | 95.7 | RP | NaCl (1 M, aq.) | 4.3 |
|  |  |  | NP^d^ | MEK | 96.4 | NP | NaCl (1 M, aq.) | 3.5 |
| 4 | [^99m^Tc]Tc-**11** | 96.5 | VWR paper | MEK | 95.0 | VWR paper | NH_4_OAc (1 M, aq.)/DMF (1/1) | 5.6 |
|  |  |  | ITLC-SG | MEK | 96.6 | ITLC-SG | NH_4_OAc (1 M, aq.)/DMF (1/1) | 5.6 |
|  |  |  | RP | MEK | 94.4 | RP | NH_4_OAc (1 M, aq.)/DMF (1/1) | 5.9 |
|  |  |  | NP | MEK | 96.0 | NP | NH_4_OAc (1 M, aq.)/DMF (1/1) | 5.6 |
|  |  |  | - | - | - | VWR paper | MeCN/H_2_O (9/1) + 10% 2 M NaOAc (aq.) + 1% TFA | 94.2 |
|  |  |  | - | - | - | ITLC-SG | MeCN/H_2_O (9/1) + 10% 2 M NaOAc (aq.) + 1% TFA | 90.8 |
|  |  |  | - | - | - | RP | MeCN/H_2_O (9/1) + 10% 2 M NaOAc (aq.) + 1% TFA | 5.0 |
|  |  |  | - | - | - | NP | MeCN/H_2_O (9/1) + 10% 2 M NaOAc (aq.) + 1% TFA | 4.4 |
|  |  |  | - | - | - | VWR paper | MeCN/H_2_O (9/1) | 29.5 |
|  |  |  | - | - | - | ITLC-SG | MeCN/H_2_O (9/1) | 8.9 |
|  |  |  | - | - | - | VWR paper | CHCl_3_/MeOH (3/1) | 50.3 |
|  |  |  | - | - | - | ITLC-SG | CHCl_3_/MeOH (3/1) | 12.1 |
|  |  |  | - | - | - | NP | CHCl_3_/MeOH (3/1) | 9.3 |
|  |  |  | - | - | - | RP | CHCl_3_/MeOH (3/1) | 9.3 |
|  |  |  | - | - | - | VWR paper | MeCN/H_2_O (9/1) + 1% TFA | 63.6 |
|  |  |  | - | - | - | ITLC-SG | MeCN/H_2_O (9/1) + 1% TFA | 18.8 |
|  |  |  | - | - | - | VWR paper | MeCN + 1 % TFA | 35.4 |
|  |  |  | - | - | - | ITLC-SG | MeCN + 1 % TFA | 11.7 |
| 5 | [^99m^Tc]Tc-**11** | 97.4 | Whatman 31ET | MEK | 85.5 | Whatman 31ET | MeCN/H_2_O (9/1) + 1% TFA | 60.3 |
|  |  |  | - | - | - | Whatman 31ET | NaCl (1 M, aq.) | 18.2 |
|  |  |  | - | - | - | Whatman 31ET | CHCl_3_/MeOH (3/1) | 40.0 |
| 6 | [^99m^Tc]Tc-**11** | 98.6 | - | - | - | Whatman 1 | NaCl (1 M, aq.) | 12.7 |
|  |  |  | - | - | - | Whatman 31ET | NaCl (1 M, aq.) | 13.7 |
|  |  |  | - | - | - | VWR paper | MeCN/H_2_O (9/1) + 10% 2 M NaOAc (aq.) + 1% TFA | 57.8 |
|  |  |  | - | - | - | Whatman 1 | MeCN/H_2_O (9/1) + 10% 2 M NaOAc (aq.) + 1% TFA | 37.4 |
|  |  |  | - | - | - | Whatman 31ET | MeCN/H_2_O (9/1) + 10% 2 M NaOAc (aq.) + 1% TFA | 59.1 |
|  |  |  | - | - | - | Whatman 31ET | CHCl_3_/MeOH (3/1) | 69.7 |
|  |  |  | - | - | - | Whatman 1 | CHCl_3_/MeOH (3/1) | 40.4 |
|  |  |  | - | - | - | VWR paper | MeCN/H_2_O (9/1) + 1% TFA | 74.0 |
|  |  |  | - | - | - | Whatman 31ET | MeCN/H_2_O (9/1) + 1% TFA | 72.6 |
|  |  |  | - | - | - | Whatman 1 | MeCN/H_2_O (9/1) + 1% TFA | 78.8 |
|  |  |  | - | - | - | Whatman 31ET | MeCN/H_2_O (9/1) + 5% TFA | 77.3 |
|  |  |  | - | - | - | Whatman 1 | MeCN/H_2_O (9/1) + 5% TFA | 80.1 |
|  |  |  | - | - | - | Whatman 31ET | MeCN/H_2_O (8/2) + 5% TFA | 93.2 |
|  |  |  | - | - | - | Whatman 1 | MeCN/H_2_O (8/2) + 5% TFA | 94.7 |
| 7 | [^99m^Tc]Tc-**11** | 97.4 | Whatman 1 | MEK | 95.8 | Whatman 1 | MeCN/H_2_O (8/2) + 1% TFA | 91.4 |
|  |  |  | Whatman 31ET | MEK | 86.5 | Whatman 31ET | MeCN/H_2_O (8/2) + 1% TFA | 93.1 |
|  |  |  | - | - | - | Whatman 1 | MeCN/H_2_O (8/2) + 5% TFA | 93.8 |
|  |  |  | - | - | - | Whatman 31ET | MeCN/H_2_O (8/2) + 5% TFA | 86.9 |
| 8 | [^99m^Tc]Tc-**12** | 94.1 | Whatman 1 | MEK | 95.2 | Whatman 1 | MeCN/H_2_O (8/2) + 1% TFA | 92.7 |
|  |  |  | Whatman 31ET | MEK | 88.7 | Whatman 31ET | MeCN/H_2_O (8/2) + 1% TFA | 90.6 |
|  |  |  | - | - | - | Whatman 1 | MeCN/H_2_O (8/2) + 5% TFA | 87.9 |
|  |  |  | - | - | - | Whatman 31ET | MeCN/H_2_O (8/2) + 5% TFA | 89.3 |
| 9 | [^99m^Tc]Tc-**15** | 95.2 | Whatman 1 | MEK | 91.9 | Whatman 1 | MeCN/H_2_O (8/2) + 1% TFA | 91.7 |
|  |  |  | Whatman 31ET | MEK | 87.4 | Whatman 31ET | MeCN/H_2_O (8/2) + 1% TFA | 89.9 |
|  |  |  | - | - | - | Whatman 1 | MeCN/H_2_O (8/2) + 5% TFA | 92.1 |
|  |  |  | - | - | - | Whatman 31ET | MeCN/H_2_O (8/2) + 5% TFA | 90.4 |
| 10 | [^99m^Tc]Tc-**11** | 98.7 | Whatman 1 | MEK | 96.0 | Whatman 1 | MeCN/H_2_O (8/2) + 5% TFA | 95.7 |
| 11 | [^99m^Tc]Tc-**16** | >99 | Whatman 1 | MEK | 95.1 | Whatman 1 | MeCN/H_2_O (8/2) + 5% TFA | 12.2 |
|  |  |  | Whatman 1 | MEK | 95.1 | Whatman 1 | MeCN/H_2_O (8/2) + 5% TFA | 82.3 |
|  |  |  | - | - | - | Whatman 1 | MeCN/H_2_O (8/2) + 1% TFA | 52.8 |
|  |  |  | - | - | - | Whatman 31ET | MeCN/H_2_O (8/2) + 5% TFA | 74.7 |
|  |  |  | - | - | - | Whatman 31ET | MeCN/H_2_O (8/2) + 1% TFA | 60.0 |
| 12 | [^99m^Tc]Tc-**17** | 97.5 | Whatman 1 | MEK | 97.2 | Whatman 1 | MeCN/H_2_O (8/2) + 5% TFA | 87.8 |
| 13 | [^99m^Tc]Tc-**15** | 97.2 | Whatman 1 | MEK | 97.2 | Whatman 1 | MeCN/H_2_O (8/2) + 5% TFA | 93.5 |
| 14 | [^99m^Tc]Tc-**16** | 97.5 | Whatman 1 | MEK | 95.0 | Whatman 1 | MeCN/H_2_O (8/2) + 5% TFA | 85.5 |
| 15 | [^99m^Tc]Tc-**17** | 98.0 | Whatman 1 | MEK | 95.6 | Whatman 1 | MeCN/H_2_O (8/2) + 5% TFA | 78.8 |
| 16 | [^99m^Tc]Tc-**15** | 96.6 | Whatman 1 | MEK | 95.8 | Whatman 1 | MeCN/H_2_O (8/2) + 5% TFA | 91.5 |
| 17 | [^99m^Tc]Tc-**16** | 97.1 | Whatman 1 | MEK | 96.2 | Whatman 1 | MeCN/H_2_O (8/2) + 5% TFA | 82.5 |
| 18 | [^99m^Tc]Tc-**17** | 98.2 | Whatman 1 | MEK | 96.0 | Whatman 1 | MeCN/H_2_O (8/2) + 5% TFA | 80.4 |
| 19 | [^99m^Tc]Tc-**15** | 95.1 | Whatman 1 | MEK | 96.1 | Whatman 1 | MeCN/H_2_O (8/2) + 5% TFA | 92.1 |
| 20 | [^99m^Tc]Tc-**13** | 95.4 | Whatman 1 | MEK | 96.4 | Whatman 1 | MeCN/H_2_O (8/2) + 5% TFA | 79.0 |
| 21 | [^99m^Tc]Tc-**12** | 98.6 | Whatman 1 | MEK | 97.3 | Whatman 1 | MeCN/H_2_O (8/2) + 5% TFA | 92.3 |
| 22 | [^99m^Tc]Tc-**12** | 98.2 | Whatman 1 | MEK | 96.9 | Whatman 1 | MeCN/H_2_O (8/2) + 5% TFA | 94.1 |
| 23 | [^99m^Tc]Tc-**13** | 97.3 | Whatman 1 | MEK | 98.0 | Whatman 1 | MeCN/H_2_O (8/2) + 5% TFA | 69.2 |
| 24 | [^99m^Tc]Tc-**12** | 95.4 | Whatman 1 | MEK | 95.0 | Whatman 1 | MeCN/H_2_O (8/2) + 5% TFA | 94.7 |
| 25 | [^99m^Tc]Tc-**13** | 96.0 | Whatman 1 | MEK | 95.0 | Whatman 1 | MeCN/H_2_O (8/2) + 5% TFA | 80.8 |
| 26 | [^99m^Tc]Tc-**11** | 96.0 | Whatman 1 | MEK | 97.1 | Whatman 1 | MeCN/H_2_O (8/2) + 5% TFA | 93.2 |
| 27 | [^99m^Tc]Tc-**15** | 95.1 | Whatman 1 | MEK | 96.6 | Whatman 1 | MeCN/H_2_O (8/2) + 5% TFA | 92.4 |
| 28 | [^99m^Tc]Tc-**16** | 96.6 | Whatman 1 | MEK | 95.0 | Whatman 1 | MeCN/H_2_O (8/2) + 5% TFA | 80.4 |
| 29 | [^99m^Tc]Tc-**17** | 95.3 | Whatman 1 | MEK | 95.0 | Whatman 1 | MeCN/H_2_O (8/2) + 5% TFA | 83.1 |
| 30 | [^177^Lu]Lu-**4** | 98.6 | ITLC-SG | Citrate^e^ | 99.3 | Whatman 1 | MeCN/H_2_O (8/2) + 5% TFA | 99.7 |
| 31 | [^177^Lu]Lu-**2** | 98.2 | ITLC-SG | Citrate | 98.4 | Whatman 31ET | MeCN/H_2_O (8/2) + 5% TFA | 95.4 |
| 32 | [^177^Lu]Lu-**4** | 98.3 | ITLC-SG | Citrate | 98.9 | RP | NH_4_OAc (1 M, aq.)/DMF (1/1) | 0.8 |
|  |  |  | - | - | - | Whatman 1 | MeCN/H_2_O (8/2) + 5% TFA | 97.9 |
|  |  |  | - | - | - | Whatman 31ET | MeCN/H_2_O (8/2) + 5% TFA | 95.5 |
| 33 | [^177^Lu]Lu-**2** | 97.4 | ITLC-SG | Citrate | 98.1 | RP | NH_4_OAc (1 M, aq.)/DMF (1/1) | 1.2 |
|  |  |  | - | - | - | Whatman 1 | MeCN/H_2_O (8/2) + 5% TFA | 66.6 |
|  |  |  | - | - | - | Whatman 31ET | MeCN/H_2_O (8/2) + 5% TFA | 89.5 |
| 34 | [^177^Lu]Lu-**4** | 97.7 | ITLC-SG | Citrate | 99.1 | Whatman 1 | MeCN/H_2_O (8/2) + 5% TFA | 99.8 |
| 35 | [^177^Lu]Lu-**2** | 97.1 | ITLC-SG | Citrate | 98.6 | Whatman 31ET | MeCN/H_2_O (8/2) + 5% TFA | 92.4 |
| 36 | [^177^Lu]Lu-**4** | 97.4 | ITLC-SG | Citrate | 99.2 | Whatman 1 | MeCN/H_2_O (8/2) + 5% TFA | 99.7 |
| 37 | [^177^Lu]Lu-**2** | 96.3 | ITLC-SG | Citrate | 98.3 | Whatman 31ET | MeCN/H_2_O (8/2) + 5% TFA | 87.9 |
| 38 | [^99m^Tc]Tc-**11** | 96.5 | Whatman 1 | MEK | 95.1 | Whatman 1 | MeCN/H_2_O (8/2) + 5% TFA | 94.5 |
| 39 | [^99m^Tc]Tc-**12** | 95.8 | Whatman 1 | MEK | 96.0 | Whatman 1 | MeCN/H_2_O (8/2) + 5% TFA | 95.6 |
| 40 | [^99m^Tc]Tc-**13** | 97.7 | Whatman 1 | MEK | 95.4 | Whatman 1 | MeCN/H_2_O (8/2) + 5% TFA | 96.0 |
| 41 | [^99m^Tc]Tc-**14** | 95.4 | Whatman 1 | MEK | 95.1 | Whatman 1 | MeCN/H_2_O (8/2) + 5% TFA | 94.0 |
| 42 | [^99m^Tc]Tc-**15** | 97.6 | Whatman 1 | MEK | 96.2 | Whatman 1 | MeCN/H_2_O (8/2) + 5% TFA | 92.8 |
| 43 | [^99m^Tc]Tc-**16** | 98.3 | Whatman 1 | MEK | 96.0 | Whatman 1 | MeCN/H_2_O (8/2) + 5% TFA | 91.7 |
| 44 | [^99m^Tc]Tc-**17** | 95.2 | Whatman 1 | MEK | 95.7 | Whatman 1 | MeCN/H_2_O (8/2) + 5% TFA | 81.2 |
| 45 | [^177^Lu]Lu-**4** | 96.1 | ITLC-SG | Citrate | 99.2 | Whatman 1 | MeCN/H_2_O (8/2) + 5% TFA | 99.4 |
| 46 | [^177^Lu]Lu-**2** | 97.2 | ITLC-SG | Citrate | 96.8 | Whatman 31ET | MeCN/H_2_O (8/2) + 5% TFA | 95.1 |
| 47 | [^99m^Tc]Tc-**11** | 95.8 | Whatman 1 | MEK | 96.8 | Whatman 1 | MeCN/H_2_O (8/2) + 5% TFA | 94.2 |
| 48 | [^99m^Tc]Tc-**14** | 95.2 | Whatman 1 | MEK | 95.6 | Whatman 1 | MeCN/H_2_O (8/2) + 5% TFA | 90.3 |
| 49 | [^99m^Tc]TcO_4_^- f^ | - | Munktell paper | MEK | 96.5^g^ | - | - | - |
|  |  | - | VWR paper | MEK | 95.3^g^ | VWR paper | NaCl (1 M, aq.) | 30.2 |
|  |  | - | ITLC‑SG | MEK | 96.1^g^ | ITLC‑SG | NaCl (1 M, aq.) | 95.5 |
|  |  | - | RP | MEK | 29.4^g^ | RP | NaCl (1 M, aq.) | 95.2 |
|  |  |  | NP | MEK | 94.9^g^ | NP | NaCl (1 M, aq.) | 96.0 |
| 50 | [^99m^Tc]TcO_4_^- f^ | - | Whatman 1 | MEK | 98.7^g^ | Whatman 31ET | MeCN/H_2_O (8/2) + 1% TFA | 97.1 |
|  |  | - | Whatman 31ET | MEK | 96.2^g^ | Whatman 1 | MeCN/H_2_O (8/2) + 1% TFA | 93.9 |
|  |  | - | - | - | - | Whatman 31ET | MeCN/H_2_O (8/2) + 5% TFA | 97.9 |
|  |  | - | - | - | - | Whatman 1 | MeCN/H_2_O (8/2) + 5% TFA | 98.1 |

- 1. ***In vitro* data**

**Table S2:** CCK-2R-binding affinities (IC_50,inverse_), internalization (%), lipophilicity (logD_7.4_), HSA binding (%), stability in human serum (%), RCP of radioligand in stock solution after distinct time points (%) and radio-RP-HPLC retention times (t_R_) of the investigated compounds.

| CCK-2R ligand | IC_50,inverse_ [nM]^a^ | Internali-zation [%]^b^ | logD_7.4_ | HSA binding [%] | Stability in human serum [% intact]^c^ | RCP of radioligand in labeling solution [%] (time after EOS, storage temperature) | t*_R_* [HPLC]^d^ |
| --- | --- | --- | --- | --- | --- | --- | --- |
| [^177^Lu]Lu‑2 | 28.2 ± 7.9 | 12.6 ± 0.4 [% of applied dose] = 100% | ‑3.80 ± 0.33 | 60.8 ± 2.8 | n.d. | 97.2 (2 d, 4 °C) | 6.12 |
| [^177^Lu]Lu‑4 | 40.3 ± 7.1 | 152.1 ± 0.2 | -2.66 ± 0.16 | 59.9 ± 1.2 | 97.9  97.1 (24 h) | 96.1 (2 d, 4 °C) | 11.8 |
| [^99m^Tc]Tc‑11 | 27.9 ± 5.7 | 223.8 ± 0.1 | -2.09 ± 0.03 | 53.2 ± 1.8 | 95.2 | 99.0 (7 h, r.t.) | 9.45 |
| [^99m^Tc]Tc‑12 | 16.9 ± 3.9 | 145.0 ± 0.1 | -1.89 ± 0.14 | 89.1 ± 1.4 | 93.0 | 97.1 (5 h, r.t.) | 9.75 |
| [^99m^Tc]Tc‑13 | 19.8 ± 1.3 | 66.8 ± 0.0 | -2.65 ± 0.06 | 51.9 ± 2.2 | 32.1 | n.d. | 7.61 |
| [^99m^Tc]Tc‑14 | 21.3 ± 8.1 | 107.3 ± 0.2 | -1.68 ± 0.07 | 52.3 ± 1.7 | 38.6 | 95.4 (6 h, r.t.) | 7.78 |
| [^99m^Tc]Tc‑15 | 31.0 ± 4.3 | 219.5 ± 0.1 | -0.95 ± 0.12 | 78.1 ± 0.7 | 87.0 | 96.8 (6 h, r.t.) | 10.4 |
| [^99m^Tc]Tc‑16 | 33.5 ± 3.3 | 145.2 ± 0.1 | -2.42 ± 0.11 | 47.5 ± 1.7 | 95.3 | 98.4 (6 h, r.t.) | 8.93 |
| [^99m^Tc]Tc‑17 | 28.6 ± 1.7 | 102.4 ± 0.1 | -2.56 ± 0.11 | 51.4 ± 0.8 | 95.1 | 98.2 (5.5 h, r.t.) | 9.15 |

^a^AR42J cells (2 × 10^5^ cells/well), ^nat^Lu‑DOTA‑PP‑F11N as standard competitor in increasing concentrations (10^‑11^ - 10^‑5^ M/well), radioligands of interest added in a concentration of 1.2 nM/well, incubation at 37 °C for 3 h. ^b^Internalization values corrected for unspecific binding and normalized to the external reference [^177^Lu]Lu-DOTA-PP‑F11N (12.6 ± 0.4% of the applied activity as CCK‑2 receptor‑mediated internalization at 1 h, 37 °C, 1.2 nM/well, 3 × 10^5^ cells/well, PLL‑coated plates). ^c^Incubation at 37 °C for 4 h. ^d^30-50% MeCN (2% H_2_O, 0.1% TFA) in H_2_O (0.1% TFA) in 20 min, MultoKrom 100-5 C18 column for ^177^Lu-labeled peptides; 20‑80% MeCN (2% H_2_O, 0.1% TFA) in H_2_O (0.1% TFA) in 15 min, MultoHigh Bio 300-5 C4 column for ^99m^Tc-labeled peptides. Data for binding (IC_50,inverse_) and internalization (n = 3) as well as for logD_7.4_ and HSA binding (n = 6) are expressed as mean ± standard deviation unless otherwise stated.

1. **LIST OF ABBREVIATIONS**

BSA Bovine serum albumin

CCK-2R Cholecystokinin 2 receptor

Dap 2,3-Diaminopropionic acid

DCM Dichloromethane

Dde *N*-1-(4,4-dimethyl-2,6-dioxocyclohex-1-ylidene)-3-ethyl

DIC *N,N'*-Diisopropylcarbodiimide

DIPEA *N,N*-Diisopropylethylamine

DMF *N,N*-Dimethylformamide

DMSO Dimethyl sulfoxide

DOTA 1,4,7,10-Tetraazacyclododecane-1,4,7,10-tetraacetic acid

EDTA Ethylenediaminetetraacetic acid

EOS end of synthesis

EtOH Ethanol

FBS Fetal bovine serum

Fmoc 9-Fluorenylmethyloxycarbonyl

GP General procedure

HBSS Hank’s buffered salt solution

HOAt 1-Hydroxy-7-azabenzotriazole

IC_50, inverse_ Inverse half-maximal inhibitory concentration

ITLC-SG instant thin‑layer chromatography paper impregnated with silica gel

MeCN Acetonitrile

MEK methyl ethyl ketone

MeOH methanol

N_4_Boc_4_ *N,N',N'',N'''*-Tetrakis(*tert*-butyloxycarbonyl)-6-carboxy-1,4,8,11-tetraazaundecane

NHS *N*-Hydroxysuccinimide

NMP *N*-Methyl-2-pyrrolidon

NMR Nuclear magnetic resonance

NP normal phase

PBS Phosphate‑buffered saline

PLL Poly‑L‑lysine

RCP Radiochemical purity

*R*_f_ retention factor

RP reversed phase

RP‑HPLC Reversed‑phase high‑performance liquid chromatography

r.t. room temperature

SD standard deviation

SiFA-BA Silicon-based fluoride acceptor bound to benzoic acid;

in this study SiFA‑BA refers to 4-(Di-*tert*-butylfluorosilyl)benzoic acid

SiOH‑BA 4-(Di-*tert*-butyl(hydroxy)silyl)benzoic acid

SPPS Solid‑phase peptide synthesis

TBTU *O*-(Benzotriazol-1-yl)-*N,N,N',N'*-tetramethyluronium tetrafluoroborate

*t*Bu *tert*‑Butyl

TFA Trifluoroacetic acid

THF Tetrahydrofuran

TIPS Triisopropylsilane

TP-H_2_O Tracepur^®^-H_2_O

1. **REFERENCES**

1. Iovkova L, Wängler B, Schirrmacher E, Schirrmacher R, Quandt G, Boening G, et al. para-Functionalized Aryl-di-tert-butylfluorosilanes as Potential Labeling Synthons for 18F Radiopharmaceuticals. Chemistry – A European Journal. 2009;15(9):2140-7.

2. Wurzer AJ, Wester H-J, Eiber MJ, inventors; Technische Universitaet Muenchen

Technische Universitaet Muenchen - Klinikum Rechts der Isar, assignee. PSMA binding dual mode radiotracer and therapeutic patent WO2020157177. 2020.

3. Günther T, Konrad M, Stopper L, Kunert JP, Fischer S, Beck R, et al. Optimization of the Pharmacokinetic Profile of [(99m)Tc]Tc-N(4)-Bombesin Derivatives by Modification of the Pharmacophoric Gln-Trp Sequence. Pharmaceuticals (Basel). 2022;15(9).

4. Günther T, Holzleitner N, Di Carlo D, Urtz-Urban N, Lapa C, Wester H-J. Development of the First 18F-Labeled Radiohybrid-Based Minigastrin Derivative with High Target Affinity and Tumor Accumulation by Substitution of the Chelating Moiety. Pharmaceutics. 2023;15(3):826.
